# Mapping Regioisomer-Dependent Buchwald–Hartwig C-N Coupling: Bromoimidazo[1,5-a]pyridines as a Model Electrophile Series

**DOI:** 10.3390/molecules31132339

**Published:** 2026-07-03

**Authors:** Svitlana O. Sotnik, Svitlana V. Stetsenko, Illia M. Pavliei, Oleksii A. Brusylovets, Oleksandr A. Pokholenko, Galyna P. Grabchuk, Olexandr Ye. Pashenko, Dmytro M. Volochnyuk, Serhiy V. Ryabukhin

**Affiliations:** 1 Enamine Ltd., 78 Winston Churchill Street., 02094 Kyiv, Ukraines.stetsenko@enamine.net (S.V.S.); ilia.pavley@gmail.com (I.M.P.); o.brusylovets@enamine.net (O.A.B.); o.pokholenko@enamine.net (O.A.P.); alev.pashenko@gmail.com (O.Y.P.); 2 L.V. Pisarzhevskii Institute of Physical Chemistry, National Academy of Sciences of Ukraine, Prospekt Nauki 31, 03028 Kyiv, Ukraine; 3 Institute of High Technologies, Taras Shevchenko National University of Kyiv, 60 Volodymyrska Street, 01033 Kyiv, Ukraine; grabchyk@knu.ua; 4 Enamine Scientific Research Institute, 78 Winston Churchill Street, 02094 Kyiv, Ukraine; 5 Institute of Organic Chemistry, National Academy of Sciences of Ukraine, 5 Academik Kukhar Street, 02094 Kyiv, Ukraine

**Keywords:** Buchwald–Hartwig reaction, C-N cross-coupling, high-throughput screening, heteroaryl bromides, imidazo[1,5-a]pyridine, palladium catalysis, ligand/base screening

## Abstract

Buchwald–Hartwig C-N coupling is a central method for constructing (hetero)aryl–nitrogen bonds. Yet condition translatability is often problematic from one substrate to another, even among closely related substrates, especially for heteroaryl halides. In this work, we demonstrate an approach to solving this task using the six available bromoimidazo[1,5-a]pyridine regioisomers and a representative nucleophile panel comprising benzamide, aniline, morpholine, and benzylamine as a model study. A limited-scale HTE campaign was conducted with an in-house ligand set, a fixed palladium source, and two bases. Reaction performance was assessed by LCMS with internal standard calibration, and targeted hits were verified by preparative re-runs and NMR-confirmed product assignment. The resulting conversion and product yield maps reveal strong dependence on bromide position, nucleophile class, and ligand/base selection. The 6- and 8-bromo isomers show the broadest productive reactivity profiles, whereas the 5- and 7-isomers are active in narrow condition windows. The imidazole–ring 1- and 3-bromo isomers react readily but do not provide isolable, structurally confirmed target products. Methodologically, this work demonstrates that a compact HTE workflow can rapidly define useful ligand/base combinations for heteroaryl bromides while preventing misleading conclusions from conversion-only analysis. The same approach can be applied as an early-stage reactivity screen for other heteroaryl halide series.

## 1. Introduction

Palladium-catalyzed Buchwald–Hartwig C(sp^2^)-N coupling, encompassing both the amination and amidation of (hetero)aryl halides, is among the most widely used transformations in modern organic and medicinal chemistry [1,2,3,4,5,6]. Nitrogen-containing heteroaromatics are widely represented in pharmaceuticals, agrochemicals, and functional materials, and direct N-arylation often offers the shortest and most practical route to N-substituted heterocycles [7,8]. Among aryl and heteroaryl halides, bromides offer a reasonable balance of availability, stability, and reactivity in Buchwald–Hartwig C-N coupling. However, in heteroaryl bromides, this synthetic convenience is limited by the strong dependence of reactivity on the nature of the heterocyclic core and on the position of the C-Br bond. The possible coordination of a heteroatom lone pair to palladium, undesired catalyst poisoning, and slowed reductive elimination repeatedly shut down catalytic cycles that proceed smoothly with simple aryl bromides [9,10,11]. Consequently, different heteroaryl electrophile classes frequently require specifically optimized ligand and base combinations and reaction conditions screening. Such substrate-specific behavior has been reported for 2-chloropyrimidines [12], five-membered azoles [13,14,15], base-sensitive heterocycles coupled with aliphatic amines [16], and primary amides on (hetero)aryl chlorides [17,18]. The ligand-free “naked nickel” protocol [19] and the design of aqueous, biphasic, or solvent-free conditions [20,21,22] have further broadened the existing toolbox. Still, they have not eliminated the fundamental need for electrophile-dependent tuning of reactions.

A practical consequence of this complexity is that no versatile structure–reactivity rules apply across the heteroaromatic halide series. For nearly every new scaffold, the optimal combination of palladium source, supporting ligand, base, and solvent must be re-established empirically [23,24]. High-throughput experimentation (HTE), in which dozens to hundreds of catalytic reactions are run in parallel in 24- or 96-well plates, is currently the most efficient approach to this problem [25,26,27]. In addition to accelerating optimization, HTE generates structured datasets suitable for statistical and machine learning analyses, which begin to reveal underlying reactivity patterns and provide predictive condition substrate maps [28,29,30,31]. In particular, the accessible 96-well plate protocol [25] has become a standardized template for the rapid scanning of ligand/base space in Buchwald–Hartwig amination and can be implemented without dedicated robotic platforms. Mechanistic and kinetic studies of five-membered heteroaryl halides further show that off-cycle and catalyst decomposition pathways are strongly substrate-dependent. These findings support systematic, data-driven screening over isolated, case-by-case optimization [28,31].

For this purpose, imidazo[1,5-a]pyridine provides a compact platform with six available bromination sites, enabling the study of position-dependent reactivity in Buchwald–Hartwig C-N couplings within a single heteroaromatic core. Its use here is primarily methodological, but the scaffold also has practical relevance: imidazo[1,5-a]pyridines appear in fluorescent dyes and luminescent metal complexes [32,33], provide direct access to N-heterocyclic carbene precursors and their axially chiral derivatives [34,35], and are represented in medicinal chemistry programs, including 5-HT_4_ receptor partial agonists [36], tubulin/PI3K/Akt modulators [37], GSK-3β inhibitors [38], GCN2 modulators, and METTL3 inhibitors [39,40]. Despite this relevance, reports of Pd-catalyzed C-N coupling on the imidazo[1,5-a]pyridine scaffold are absent from the peer-reviewed literature. In the patent literature, we found only one precedent describing the selective Buchwald–Hartwig-type amination of a 6-bromo-1-iodo derivative at the iodinated position, preserving the bromide for subsequent Suzuki coupling [39]. However, this example provides no information about the reactivity of the imidazo[1,5-a]pyridine bromides in catalytic C-N couplings, leaving this field entirely unexplored.

**Figure 1:**
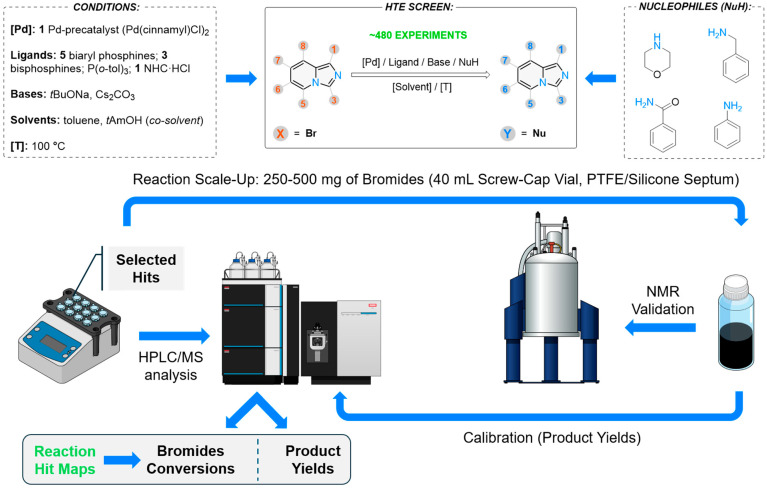
Workflow for the HTE Buchwald–Hartwig screen. Primary reactions were performed in 800 µL crimp-top vials housed in a Para-dox^®^ 24-position aluminum block, while selected hits were scaled in 40 mL septum-sealed borosilicate vials. LCMS/HPLC hit maps were first generated from bromide conversion, followed by product peak assignment and product yield calibration using isolated, NMR-confirmed products.

In this work, we aimed to evaluate position-dependent reactivity within a single heteroaromatic scaffold. We examined the six available bromoimidazo[1,5-a]pyridine regioisomers in Buchwald–Hartwig C-N coupling using a representative nucleophile panel comprising benzamide, aniline, morpholine, and benzylamine, adapted from a reported 96-well screening protocol for reaction optimization [25]. The study design is summarized in Figure 1 (see the Supplementary Materials for details on the ligands). The resulting 24 substrate/nucleophile combinations were evaluated in our dedicated HTE catalyst screening laboratory using [Pd(cinnamyl)Cl]_2_ as the precatalyst [25], with in-house-designed sets of ligands and bases. Reaction performance was monitored by LCMS/HPLC using internal standard-normalized analysis, which provided initial conversion and crude product distribution data based on calibration with the starting material. Selected hits were then isolated preparatively and characterized by NMR, which allowed us to unambiguously assign the corresponding LCMS peaks and to verify the initial screening results using confirmed product samples. The dataset reveals a strong dependence of reaction performance on both the bromide position and the nucleophile type. In particular, the imidazole–ring bromides give more complex outcomes than the pyridine–ring bromides, which is consistent with the documented challenges associated with Buchwald–Hartwig coupling of azolyl halides and related five-membered heteroaryl electrophiles [10,16,41]. Overall, our study provides a comprehensive experimental map of bromine regioposition-dependent reactivity for the imidazo[1,5-a]pyridine core in Buchwald–Hartwig C-N couplings. In a broader context, it illustrates a practical, readily transferable screening workflow for evaluating other heterocyclic bromide series.

## 2. Results and Discussion

**Scheme 1:**
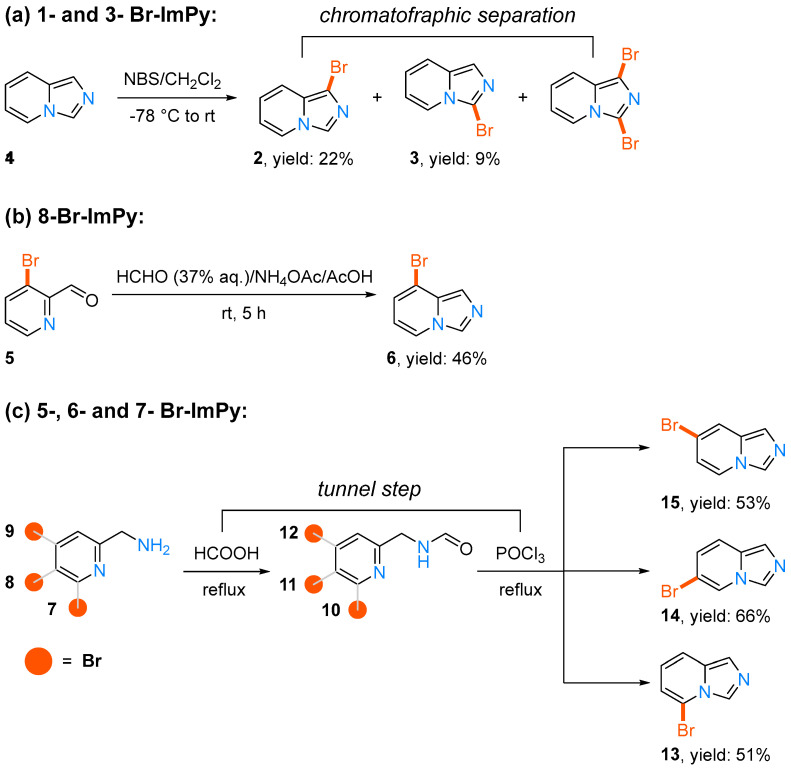
Synthesis of the six bromoimidazo[1,5-a]pyridine regioisomers (**2**, **3**, **6**, **13**–**15**) used in this study. (**a**) NBS, CH_2_Cl_2_, −78 °C to rt, affording 1- and 3-bromo isomers **2** and **3**; (**b**) HCHO (37% aq.), NH_4_OAc, AcOH, rt, 5 h, affording the 8-bromo isomer **6** from 3-bromopyridine-2-carbaldehyde **5**; (**c**) HCOOH at reflux, followed by reflux in POCl_3_, yielding 5-, 6-, and 7-bromides (**13**–**15**) from the corresponding (bromopyridin-2-yl)methanamines (**7**–**9**).

The six bromoimidazo[1,5-a]pyridine regioisomers used in this study were prepared following previously reported literature protocols, as summarized in Scheme 1. The 1- and 3-bromo regioisomers (**2** and **3**, Scheme 1a) were obtained from the parent imidazo[1,5-a]pyridine by N-bromosuccinimide (NBS) bromination, which afforded a mixture of the C-1- and C-3-brominated products and the 1,3-dibromide, which were separated chromatographically with 22 and 9% yields, respectively [42]. The 8-bromo isomer **6** was obtained in 46% yield from 3-bromopyridine-2-carbaldehyde **5** via a cyclocondensation with formaldehyde in acetic acid in the presence of ammonium acetate, following the procedure reported in the patent literature (Scheme 1b) [40]. The 5-, 6-, and 7-bromo regioisomers (compounds **13**–**15**, Scheme 1c) were prepared in 51, 66, and 53% yields, respectively, via a two-step tunnel reaction sequence (a modified literature route to imidazo[1,5-a]pyridines brominated in the pyridine ring) that involved *N*-formylation of the corresponding (6-, 5-, and 4-bromopyridin-2-yl)methanamines (**7**–**9**) in refluxing formic acid, followed by cyclodehydration of the formyl derivatives **10**–**12** in POCl_3_ [35,39,43]. All six substrates were purified to ≥95% purity (HPLC), and their structures were confirmed by NMR and HRMS prior to the screening campaign (see details in the Supplementary Materials).

**Figure 2:**
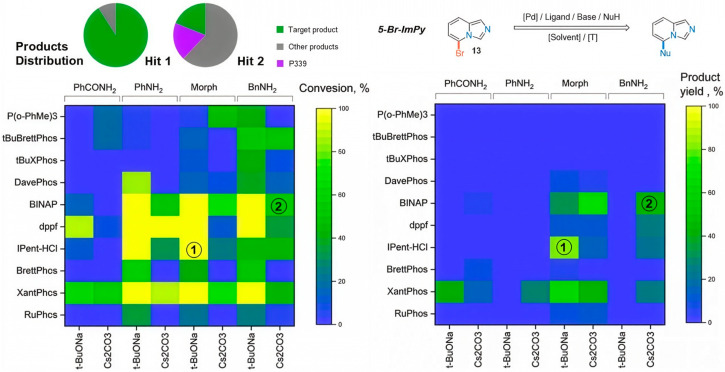
Buchwald–Hartwig C-N coupling screen of 5-bromoimidazo[1,5-a]pyridine (**5-Br-ImPy**, **13**). Conversion heatmap, chromatographic product yield heatmap, and product distribution charts are shown for the selected condition matrix. Numbered cells correspond to highlighted entries (**Hits 1** and **2**) in the pie charts; minor components below the 10% threshold are grouped as “Other products”. P339 denotes an unidentified major LCMS peak at *m*/*z* = 339.

**Figure 3:**
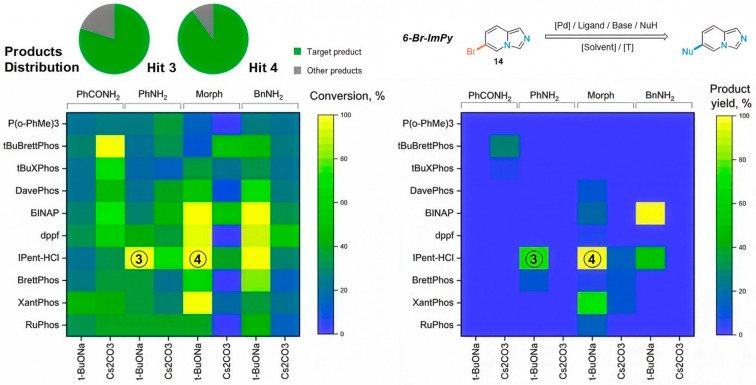
Buchwald–Hartwig C-N coupling screen of 6-bromoimidazo[1,5-a]pyridine (**6-Br-ImPy**, **14**). Conversion heatmap, chromatographic product yield heatmap, and product distribution charts are shown as in Figure 2. Numbered cells correspond to highlighted entries (**Hits 3** and **4**) in the pie charts; minor components below the 10% threshold are grouped as “Other products”.

**Figure 4:**
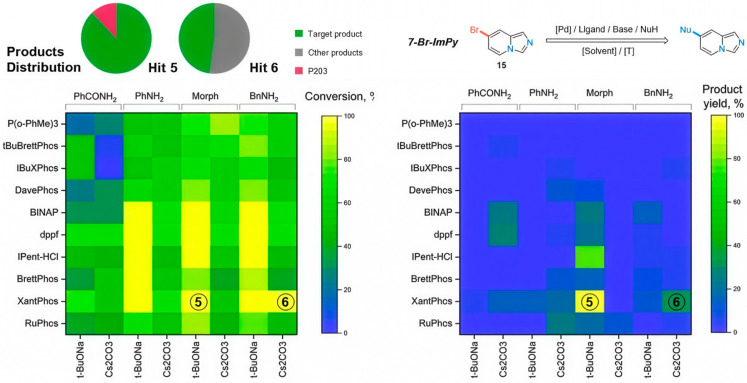
Buchwald–Hartwig CN coupling screen of 7-bromoimidazo[1,5-a]pyridine (**7-Br-ImPy**, **15**). Conversion heatmap, chromatographic product yield heatmap, and product distribution charts are shown as in Figure 2. Numbered cells correspond to highlighted entries (**Hits 5** and **6**) in the pie charts; minor components below the 10% threshold are grouped as “Other products”. P203 denotes an unidentified LCMS peak at *m*/*z* = 203.

**Figure 5:**
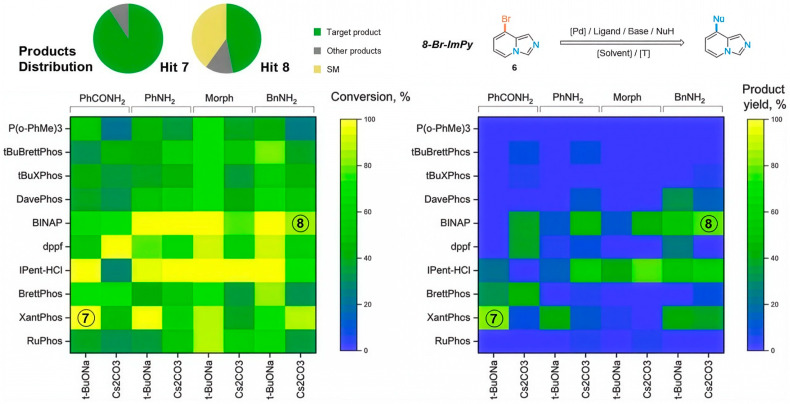
Buchwald–Hartwig C-N coupling screen of 8-bromoimidazo[1,5-a]pyridine (**8-Br-ImPy**, **6**). Conversion heatmap, chromatographic product yield heatmap, and product distribution charts are shown as in Figure 2. Numbered cells correspond to highlighted entries (**Hits 7** and **8**) in the pie charts; minor components below the 10% threshold are grouped as “Other products”. The “SM” slice (SM = Starting Material) in **Hit 8** corresponds to unreacted starting bromide **6**.

**Figure 6:**
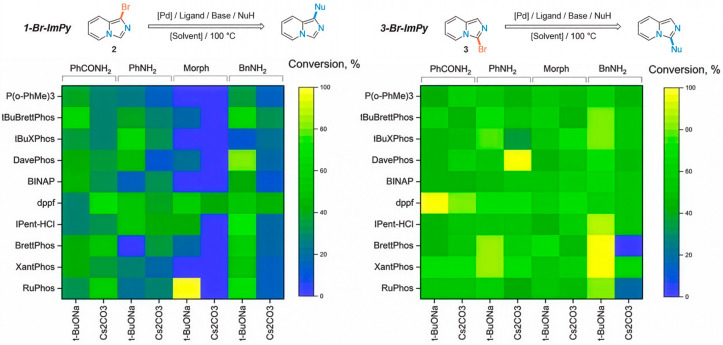
Buchwald–Hartwig C-N coupling screen of the imidazole–ring bromides 1-bromoimidazo[1,5-a]pyridine (**1-Br-ImPy**, **2**) and 3-bromoimidazo[1,5-a]pyridine (**3-Br-ImPy**, **3**). Conversion heatmaps are shown for the selected condition matrix. Product yield maps are not included because no target C-N coupling products were isolated and structurally confirmed under the screened conditions.

With the six bromoimidazo[1,5-a]pyridines (**2**, **3**, **6**, **13**–**15**) in hand (Scheme 1), the screening campaign was carried out as described in Section 3. The results are divided across the bromide regioisomers, with the pyridine–ring isomers (5-, 6-, 7-, and 8-Br) treated first and the imidazole–ring pair (1- and 3-Br) considered separately. For each substrate, the screen results are summarized in a single composite figure (Figure 2, Figure 3, Figure 4, Figure 5 and Figure 6) consisting of three elements: a conversion heatmap (bottom left), generated from the starting bromide calibration and normalized to the naphthalene internal standard added after reaction completion; a product yield heatmap (bottom right), calibrated against NMR-confirmed target products after preparative re-runs of selected entries on a 250–500 mg scale; and a set of LCMS-based product distribution pie charts (top left) for the highlighted hits (numbered **Hits 1**–**8**). In the pie charts, only species accounting for ≥10% of the post-reaction mixture are shown as separate slices; minor species are grouped as “other products”. This threshold was applied to keep the analysis focused on useful condition windows for each bromide, because reliable assignment of minor byproducts would require separate isolation and structural characterization. The complete set of pie charts for all 24 substrate/nucleophile combinations, together with the numeric conversion and yield tables, is provided in the Supporting Information (Figures S1–S8 and Tables S1 and S2). The equation in the upper-right corner of each figure shows the bromide regioisomer to which the corresponding panel refers.

The 5-bromide (**5-Br-ImPy**, **13**) is the alpha-isomer relative to the bridgehead nitrogen, which places the C-Br bond closest to the pyridine-type nitrogen lone pair and, therefore, in the environment most likely to perturb palladium speciation during the catalytic cycle [10,11]. Its conversion map (Figure 2, bottom left) is the most polarized among the pyridine–ring substrates. Bisphosphines, such as XantPhos, dppf, and BINAP, as well as the NHC precursor IPent·HCl, promote at least 80% conversion with morpholine, aniline, and benzylamine when NaO*t*Bu is used as the base. In contrast, the biaryl monophosphines BrettPhos, tBuXPhos, tBuBrettPhos, DavePhos, and RuPhos are largely inactive across the same matrix, with many entries showing no detectable conversion. Benzamide is the least reactive nucleophile in this series, with notable conversion observed only for XantPhos/NaO*t*Bu (65%) and dppf/NaO*t*Bu (89%) pairs.

After product peak assignment and calibration against NMR-confirmed target compounds, the product yield map (Figure 2, bottom right) shows that many high-conversion entries do not translate into efficient formation of the desired product. The productive (here and below, “productive” refers to formation of the assigned target C-N coupling product, not to bromide conversion alone) window narrows mainly to morpholine: IPent·HCl/NaO*t*Bu gives the morpholine adduct in 82% chromatographic product yield (**Hit 1**; pie chart shows the target product as the major component), while XantPhos/NaO*t*Bu, BINAP/Cs_2_CO_3_, and XantPhos/Cs_2_CO_3_ give 65%, 68%, and 45%, respectively. Benzylamine shows lower reactivity: BINAP/Cs_2_CO_3_ gives a 44% product yield (**Hit 2**), although the corresponding product distribution chart shows a substantial fraction of other products, including an unidentified component at *m*/*z* 339 that exceeds the 10% threshold; dppf/Cs_2_CO_3_ gives the target product in 26% yield. Benzamide is productive only under XantPhos/NaO*t*Bu conditions, giving 42% product yield. Aniline is the least effective nucleophile for **13**: despite high conversion with XantPhos, dppf, BINAP, and IPent·HCl in the presence of NaO*t*Bu, no corresponding entry provides detectable target product formation, and only the XantPhos/Cs_2_CO_3_ combination gives a moderate product yield of 29%. Overall, morpholine is clearly the most productive nucleophile for **13**, followed by benzylamine and benzamide, whereas aniline is less effective. The pronounced gap between bromide conversion and target product yield, especially for aniline and benzylamine, is consistent with the literature reports that Buchwald–Hartwig C-N coupling of heteroaryl electrophiles can be strongly affected by off-cycle catalyst speciation and decomposition [10,11,31].

6-bromide (**6-Br-ImPy**, **14**), in which the C–Br bond is in the beta position to the bridgehead nitrogen, shows a less divergent screening profile than 5-bromide **13**, particularly for amine nucleophiles, although its behavior remains strongly ligand- and nucleophile-dependent. In the conversion map (Figure 3, bottom left), both morpholine and benzylamine give around 90% bromide consumption with IPent·HCl, dppf, BrettPhos, and BINAP when NaO*t*Bu is used as the base. For aniline, only IPent·HCl gives efficient bromide consumption; both bases are viable with this ligand, but NaO*t*Bu gives the higher conversion. For benzamide, bromide consumption is observed only with certain ligand/base combinations, specifically *t*BuBrettPhos/Cs_2_CO_3_, which allows complete conversion, and BINAP/Cs_2_CO_3_ and *t*BuXPhos/Cs_2_CO_3_, which give 76% and 72%, respectively.

The product yield map for **14** (Figure 3, bottom right) retains several high-conversion amine entries, but the comparison with the conversion map shows that bromide consumption alone does not reliably indicate formation of the assigned target product. The two selected productive entries are IPent·HCl-based: IPent·HCl/NaO*t*Bu gives the aniline coupling product in 75% product yield (**Hit 3**), while the corresponding morpholine reaction gives the product in essentially quantitative yield (**Hit 4**). In both cases, the product distribution charts show the target product as the major component. BINAP/NaO*t*Bu also provides the benzylamine adduct in essentially quantitative yield, and XantPhos/NaO*t*Bu gives the morpholine product in 74%. Benzamide remains the most difficult nucleophile: only *t*BuBrettPhos/Cs_2_CO_3_ gives a productive entry, reaching 27% product yield, whereas the other ligand/base combinations provide only trace to low target product formation despite moderate conversion. Therefore, for **14**, morpholine and benzylamine are the most productive nucleophiles, followed by aniline, while benzamide is markedly less efficient. The preferred ligand/base combination remains nucleophile-dependent: IPent·HCl/NaO*t*Bu is optimal for aniline and morpholine, BINAP/NaO*t*Bu for benzylamine, and *t*BuBrettPhos/Cs_2_CO_3_ for benzamide.

7-bromide (**7-Br-ImPy**, **15**), in which the C-Br bond is in the gamma position to the bridgehead nitrogen, gives the broadest bromide consumption profile among substrates that are substituted in the pyridine ring. In the conversion map (Figure 4, bottom left), aniline, morpholine, and benzylamine show high conversion across many ligand/base combinations, with XantPhos, dppf, BrettPhos, IPent·HCl, and BINAP frequently giving at least 80% conversion and several entries reaching complete bromide consumption. Benzamide gives a narrower conversion profile, although XantPhos/NaO*t*Bu and dppf/NaO*t*Bu still give promising conversion in the 70–74% range.

Comparison with the product yield map (Figure 4, bottom right) shows that the broad conversion profile translates into target product formation only for selected entries. The clearest productive region is the morpholine/NaO*t*Bu column, where XantPhos/NaO*t*Bu gives the morpholine adduct in quantitative chromatographic product yield (**Hit 5**), and IPent·HCl/NaO*t*Bu gives 79%; dppf, BINAP, and RuPhos with NaO*t*Bu give lower yields of 23%, 26%, and 17%, respectively. The remaining nucleophiles provide only scattered productive entries. For benzylamine, XantPhos/Cs_2_CO_3_ gives 31% product yield (**Hit 6**), and the corresponding pie chart shows a substantial fraction of other products and an unidentified component at *m*/*z* 203 above the 10% threshold. In the benzamide series, dppf/Cs_2_CO_3_ and BINAP/Cs_2_CO_3_ give the best product yields, 29% and 24%, respectively, while RuPhos/Cs_2_CO_3_ gives the best result for aniline at 24%. Despite **15** readily reacting under many conditions, the formation of the assigned target products is limited to a narrower set of ligand/base combinations and nucleophiles, with XantPhos/NaO*t*Bu and morpholine being the most effective.

8-bromide (**8-Br-ImPy**, **6**), in which the C-Br bond is in the delta position to the bridgehead nitrogen and peri to the imidazole ring, shows the most evenly distributed conversion profile among the aforementioned substrates. In the conversion map (Figure 5, bottom left), all entries show at least moderate bromide consumption, and BINAP, IPent·HCl, dppf, and XantPhos each deliver high conversion across all four nucleophiles with at least one base. This behavior contrasts with the more polarized profiles observed for **5-Br-ImPy** (**13**) and **6-Br-ImPy** (**14**), indicating that **8-Br-ImPy** (**6**) is less dependent on a narrow ligand/base combination in terms of its reactivity.

The product yield map (Figure 5, bottom right) also contains the broadest set of productive entries in the pyridine–ring series. XantPhos/NaO*t*Bu gives the benzamide product in 80% chromatographic product yield (**Hit 7**; the pie chart shows the target product as the major component), the best result for benzamide in the present study. For benzylamine, BINAP/Cs_2_CO_3_ gives 78% product yield (**Hit 8**); the corresponding product distribution chart shows substantial residual starting material, consistent with incomplete bromide conversion rather than extensive formation of additional byproducts. IPent·HCl/Cs_2_CO_3_ is also effective with morpholine, yielding 78%, and it provides moderate yields with benzylamine and aniline, both at 59%; IPent·HCl/NaO*t*Bu produces the benzylamine product in 52%. Overall, **8-Br-ImPy** (**6**) exhibits a relatively balanced reactivity profile across the set of nucleophiles, with productive entries observed for benzamide, morpholine, benzylamine, and aniline. The result with benzamide is notable because amidation was generally the least efficient in the screen: the 5-, 6-, and 7-bromides (**13**–**15**) gave only low-to-moderate product yields. They required different ligand/base combinations, whereas **8-Br-ImPy** (**6**) gave 80% yield with XantPhos/NaO*t*Bu. This suggests that the response of amide coupling to base and ligand choice is strongly dependent on the bromide position, rather than governed by a single general condition preference across the scaffold.

Bromides in the imidazole ring, **1-Br-ImPy 2** and **3-Br-ImPy 3**, were evaluated under the same screening matrix as isomers in the pyridine one. For these substrates, only conversion data are shown (Figure 6) because no target C-N coupling products were isolated and structurally confirmed from the screened entries. However, the conversion maps provide a useful perspective on reactivity across the series of bromoimidazo[1,5-a]pyridines. 1-bromide **2** gives mostly low-to-moderate bromide consumption. The only consistently active region is the benzylamine/NaO*t*Bu column, where DavePhos, IPent·HCl, *t*BuBrettPhos, RuPhos, BrettPhos, XantPhos, and dppf give 54–83% conversion; outside this region, full conversion is observed only for the RuPhos/NaO*t*Bu combination with morpholine. In contrast, 3-bromide **3** is substantially more reactive, with much of the matrix giving 50–100% conversion and several entries achieving quantitative conversion, including dppf/NaO*t*Bu/benzamide, BrettPhos/NaO*t*Bu/benzylamine, and DavePhos/Cs_2_CO_3_/aniline.

Preparative re-runs of selected high-conversion entries from both substrates did not yield the expected target products in a form suitable for unambiguous NMR confirmation. Several LCMS traces showed peaks at the expected product mass, particularly in reactions of 1-Br-ImPy **2** with morpholine, but the corresponding materials could not be isolated cleanly; therefore, no product yields are reported. This behavior is consistent with the known condition sensitivity of azolyl bromides and related five-membered heteroaryl electrophiles in Buchwald–Hartwig amination, where productive coupling is more often limited by slow reductive elimination, off-cycle palladium speciation, and competing decomposition pathways [10,11,13,41]. Thus, the imidazole–ring bromides define a practical reactivity boundary for the present screen: the C-Br bonds are consumed under selected conditions, but the reaction mixtures do not provide isolable, structurally confirmed target *N*-arylation products within the ligand/base set examined here.

From an experimental standpoint, the four pyridine–ring bromides (**6** and **13**–**15**) show optimal reactivity under determined sets of conditions rather than under a single transferable protocol. 8-bromide **6** shows the broadest productive reactivity, with target product yields across all four nucleophiles, including entries that achieve high yields: 80% for benzamide with XantPhos/NaOtBu, 78% for benzylamine with BINAP/Cs_2_CO_3_, and 78% for morpholine with IPent·HCl/Cs_2_CO_3_. 6-bromide **14** is highly productive with amine nucleophiles, giving 75% yield with aniline and essentially quantitative yields with morpholine and benzylamine, but its coupling with benzamide remains limited to 27%. The 5- and 7-bromides (**13** and **15**) show narrower reactivity windows: **5-Br-ImPy 13** works best with morpholine, reaching 82% yield, whereas **7-Br-ImPy 15** shows high conversion under various conditions, with productive target compound formation mainly in selected entries, most notably with XantPhos/NaO*t*Bu and morpholine. Across the pyridine–ring series, morpholine is the most consistently productive nucleophile, whereas aniline and benzamide are more strongly dependent on the bromide position and the choice of ligand/base. As for bases, NaO*t*Bu appeared to be the most useful for many amine couplings, but Cs_2_CO_3_ is essential in several productive benzylamine and benzamide entries, emphasizing that both base and ligand choice must be adjusted to the bromide position and nucleophile class. XantPhos and IPent·HCl can be highlighted as the most efficient among productive ligands, but neither is versatile across the full matrix. Notably, BINAP and *t*BuBrettPhos are priority ligand choices for several entries. In general, our results support experimental electrophile/nucleophile/ligand/base matching over approaches that aim to find universal conditions.

## 3. Materials and Methods

### 3.1. High-Throughput Screening Design

The screening campaign was performed to evaluate the influence of bromide position and nucleophile class on Buchwald–Hartwig C-N coupling within the parent imidazo[1,5]-pyridine series. Six bromoimidazo[1,5-a]pyridine regioisomers (specifically, 1-, 3-, 5-, 6-, 7-, and 8-bromoimidazo[1,5-a]pyridines) were combined with four representative nitrogen nucleophiles, namely, benzamide, aniline, morpholine, and benzylamine. This nucleophile panel was adapted from a previously reported 96-well plate Buchwald–Hartwig screening protocol [25]. The resulting 24 substrate/nucleophile combinations were evaluated using [Pd(cinnamyl)Cl]_2_ as the palladium source, together with an in-house-designed set of ligands and two bases, in toluene at 100 °C.

The focused screening set was assembled from a single Pd source, [Pd(cinnamyl)Cl]_2_, chosen according to the Cook–Newman screening protocol [25] to avoid additional precatalyst optimization, in combination with 10 ligands, 2 bases, and toluene as a solvent. The ligand set comprised five biaryl monophosphines (BrettPhos, *t*BuBrettPhos, RuPhos, *t*BuXPhos, DavePhos), three bisphosphines (XantPhos, dppf, BINAP), P(o-tol)_3_, and one NHC·HCl ligand (IPent·HCl). The bases were NaO*t*Bu and Cs_2_CO_3_; the solvent was toluene. *tert*-Amyl alcohol was used for benzamide dissolution and preparation of its stock solution. The condition array was uniform across the 24 substrate/nucleophile pairs. The full screening matrix, reagent structures, and detailed condition tables are provided in the Supporting Information.

### 3.2. Screening Reaction Setup

Screening reactions were performed in a crimp-top vial in an 800 µL standard 24-Position Parallel Synthesis Reaction Block, Gen II Para-dox^®^, Analytical Sales & Service Inc. (Flanders, NJ, USA). Reactions were assembled under argon conditions in a glovebox by the addition of the bromoimidazo[1,5-a]pyridine substrate (electrophile), nucleophile, [Pd(cinnamyl)Cl]_2_, ligand, base, and solvent. Stock solutions of electrophile, nucleophile, and NaO*t*Bu in toluene were prepared in advance in the glovebox. [Pd(cinnamyl)Cl]_2_ and ligands were coated onto acid-washed glass beads (G1277, 212–300 µm, Sigma Aldrich (Darmstadt, Germany) at 1.3 wt% and 4.5 wt%, respectively, using a Resodyn Acoustic Mixer LabRAM I (Resodyn Acoustic Mixers, Butte, MT, USA) at 95× *g* for 10 min. The standard screening scale was 10 μmol of electrophile at 0.1 M concentration. The nucleophile, base, catalyst, and ligand were used in 1.2 equiv (12 μmol), 2.0 equiv (20 μmol; 2.2 equiv/22 μmol for selected entries), and 5 mol% (0.5 μmol), respectively, in 100 μL of solvent. Reactions were sealed with a rubber sealing mat and PFA film and heated at 100 °C for 16 h with stirring. No internal replicates were included in the primary screen (each unique ligand × base combination was run once per substrate/nucleophile pair). After reaction, the reaction mixtures were processed directly for LCMS analysis as described below. Approximately 480 individual reactions (including the pre-optimization steps that are not discussed in the manuscript) were performed in total.

### 3.3. LCMS Analysis of Screening Reactions

Reaction performance in the HTE screen was assessed by LCMS using internal standard-normalized analysis. After the reactions were completed, the block was opened, and toluene was removed by evaporation. A total of 20 μL aliquots of a 0.05 M naphthalene solution in DMSO were added to each vial as an internal standard during post-reaction sample preparation at 10 mol% relative to the starting bromide (1 μmol of naphthalene per 10 μmol of bromide). Reaction mixtures were diluted to 500 µL with DMSO, filtered through a syringe filter, and analyzed by LCMS. The LCMS analyses were performed on an Agilent 1290 Infinity II LCMS system equipped with DAD, ELSD, and G6135 LC/MSD detectors, using ESI ionization in positive and negative modes over an *m*/*z* range of 83–1000 *m*/*z*, with an Agilent Poroshell 120 SB-C18 column (4.6 × 30 mm, 2.7 µm), Agilent Technologies (Santa Clara, CA, USA). Mobile phase A was 100% acetonitrile (0.1% formic acid), and mobile phase B was 100% water (0.1% formic acid). The gradient ran from 0 to 1.5 min (A 0–100%, B 100–0%), with a flow rate of 3 mL/min, a column temperature of 60 °C, and an injection volume of 3 μL. UV/DAD integration was performed at 216 nm using an in-house-developed LCMS analysis toolkit [44].

Conversion values for arylbromides were calculated from starting material calibration using internal standard-normalized peak areas. Crude product content was estimated from the corresponding LCMS traces. Data acquisition and chromatographic integration were performed using Agilent OpenLab CDS 2.8; sample identifiers, Agilent Technologies (Santa Clara, CA, USA), integrated peak areas, conversion values, and crude product distribution estimates were recorded and processed in preformatted Microsoft Excel spreadsheets. Selected products were synthesized on a 250–500 mg scale, isolated preparatively, and characterized by NMR. They were used as standard samples to assign the corresponding chromatographic peaks and to verify the initial screening results. Calibration procedures and the processed screening dataset are provided in the Supporting Information.

### 3.4. Product Assignment and Preparative Verification

Selected screening hits were repeated on a preparative scale under the corresponding conditions identified in the screening campaign. Products were isolated by flash column chromatography on an ISCO^®^ Interchim puriflash XS 420, Advion Interchim Scientific (Montlucon, France); normal-phase chromatography was performed using silica ZEOprep 60/40, 63 µm. A gradient solvent system of chloroform–acetonitrile (acetonitrile 50–100%) was used to purify all reaction mixtures. Purified products were characterized by NMR and HRMS. Confirmed product samples were used to assign the corresponding LCMS peaks and to verify the initial screening results. Cases in which a peak with the expected mass did not correspond to the desired product were excluded from product yield assignment and are discussed separately where relevant. Preparative re-runs were carried out at 250 mg or 500 mg of electrophile loading.

Full preparative procedures, isolated yields, compound characterization data, and copies of NMR spectra are provided in the Experimental section and the Supporting Information.

### 3.5. Data Processing and Visualization

Primary screening data were tabulated and processed in an in-house-designed Microsoft Excel workbook for HTE catalyst screening studies. For each reaction, the workbook calculates the amounts of the aryl bromide and the desired product from the integral intensities of the corresponding analyte and internal standard peaks in the chromatogram, using calibration data. Processed conversion, selectivity, and product distribution data were imported from preformatted Microsoft Excel spreadsheets and analyzed in Python 3.12 using pandas and matplotlib. Heatmaps, axis-wise ranking plots, pairwise regioisomer comparisons, and product composition pie charts were generated from the processed screening dataset.

## 4. Conclusions

Buchwald–Hartwig amination and amidation of heteroaryl halides remain highly condition-dependent. Our study addresses this challenge by examining the six regioisomers of bromoimidazo[1,5-a]pyridine, with the substituents in all possible positions. Even within this single scaffold, no single ligand/base/nucleophile combination provided uniformly effective coupling, underscoring the limited transferability of “general” Buchwald–Hartwig conditions across regioisomeric heteroaryl bromides. The resulting dataset provides the first position-resolved reactivity map for Buchwald–Hartwig C-N coupling in the bromoimidazo[1,5-a]pyridine series. The nucleophile panel, comprising a cyclic secondary amine, a primary aliphatic amine, a primary aromatic amine, and a primary amide, enabled direct comparison of amination and amidation behavior across the full electrophile set. All pyridine–ring bromides were viable electrophiles, but their useful condition windows differed markedly: 8-Br-ImPy displayed the broadest reactivity, 6-Br-ImPy was most effective with amines, and 5-Br- and 7-Br-ImPy were productive only in narrow combinations of ligand/base/nucleophile. By contrast, the imidazole–ring bromides underwent conversion under many conditions but did not yield isolable, structurally confirmed target products in the present screen, consistent with the known behavior of azolyl bromides, in which productive Buchwald–Hartwig coupling is often limited by slow C-N reductive elimination and competing off-cycle palladium pathways.

The methodological value of the study lies in its use of a defined, compact HTE workflow that combines a fixed palladium source, an internally designed ligand set covering the main ligand families used in Buchwald–Hartwig chemistry, two bases, and LCMS analysis using internal standard calibration. Selected hits were repeated preparatively, and NMR-confirmed products were used to assign product peaks and validate product yield estimates. This two-stage workflow distinguishes simple bromide consumption from formation of the assigned target C-N coupling product, which proved essential for the results interpretation. Productive entries were distributed across NHC, bisphosphine, and biaryl monophosphine ligand classes, confirming that no single ligand family is sufficient even for the substrate series as narrow as regiosomeric bromides of a single heteroaromatic core scaffold.

In terms of synthetic utility, morpholine gave the most consistently productive response across the pyridine–ring bromides, whereas aniline and benzylamine showed greater dependence on bromide position and ligand/base pairing. Benzamide was the most demanding nucleophile overall, although the 8-bromo isomer afforded a notably efficient amidation. Beyond the specific imidazo[1,5-a]pyridine case, the study demonstrates a practical early-stage screening approach for evaluating the reactivity of heteroaryl halides in Buchwald–Hartwig-type reactions. By combining defined condition arrays with product-level analytical validation, this workflow can identify useful ligand/base regimes while avoiding misleading conclusions from conversion-only analysis. The full numerical dataset and experimental details provided in the Supporting Information are intended to support reuse of the workflow in related Buchwald–Hartwig studies.

## Data Availability

All data that support the findings of this study are available in the Supplementary Materials.

## Supplementary Materials

The following supporting information can be downloaded at: https://www.mdpi.com/article/10.3390/molecules31132339/s1, Figure S1: Target N-arylation products for the bromoimidazo[1,5-a]pyridine series. Generic substitution patterns are shown in blue; isolated, structurally confirmed products with preparative yields obtained after scale-up of selected hits on a 250–500 mg bromide scale are shown in black; unconfirmed 1- and 3-substituted targets are shown in gray; Figure S2: Structures of the ligands and the Pd precatalyst included in the Buchwald–Hartwig C-N coupling screen.; Figure S3: Reaction–mixture composition matrix for 1-bromoimidazo[1,5-a]pyridine (1-Br-ImPy, **2**) across the screened ligand/base/nucleophile combinations according to LCMS data; Figure S4: Reaction–mixture composition matrix for 3-bromoimidazo[1,5-a]pyridine (3-Br-ImPy, **3**) across the screened ligand/base/nucleophile combinations according to LCMS data; Figure S5: Reaction–mixture composition matrix for 5-bromoimidazo[1,5-a]pyridine (5-Br-ImPy, **13**) across the screened ligand/base/nucleophile combinations according to LCMS data; Figure S6: Reaction–mixture composition matrix for 6-bromoimidazo[1,5-a]pyridine (6-Br-ImPy, **14**) across the screened ligand/base/nucleophile combinations according to LCMS data; Figure S7: Reaction–mixture composition matrix for 7-bromoimidazo[1,5-a]pyridine (7-Br-ImPy, **15**) across the screened ligand/base/nucleophile combinations according to LCMS data; Figure S8: Reaction–mixture composition matrix for 8-bromoimidazo[1,5-a]pyridine (8-Br-ImPy, **6**) across the screened ligand/base/nucleophile combinations according to LCMS data; Table S1: LCMS-based conversion (%) of the bromoimidazo[1,5-a]pyridines under the screened ligand/base/nucleophile combinations. Conversion values were calculated from residual starting bromide signals normalized to the post-added naphthalene internal standard; Table S2: Chromatographic yields (%) of the assigned target products for the pyridine–ring bromoimidazo[1,5-a]pyridines under the screened ligand/base/nucleophile combinations.
