# Supplementary material for: Mapping Regioisomer-Dependent Buchwald–Hartwig C-N Coupling: Bromoimidazo[1,5-a]pyridines as a Model Electrophile Series
Source: Molecules. 2026 Jul 3;31(13):2339. doi: 10.3390/molecules31132339 (PMC13363657; doi:10.3390/molecules31132339)

Mapping Regioisomer-Dependent Buchwald-Hartwig C-N Coupling: Bromoimidazo[1,5-a]pyridines as a Model Electrophile Series

Svitlana O. Sotnik [1,2], Svitlana V. Stetsenko [1,3], Illia M. Pavliei [1,2], Oleksii A. Brusylovets [1,3], Oleksandr A. Pokholenko [1,3], Galyna P. Grabchuk [3], Olexandr Ye. Pashenko, [1,3,4,5] Dmytro M. Volochnyuk [1,3,4,5,]* and Serhiy V. Ryabukhin [1,3,4,5,]*

[1] Enamine Ltd., 78 Winston Churchill Street., 02094 Kyiv, Ukraine; s.stetsenko@enamine.net (S.V.S.); ilia.pavley@gmail.com (I.M.P.); o.brusylovets@enamine.net (O.A.B.); o.pokholenko@enamine.net (O.A.P.); alev.pashenko@gmail.com (O.Y.P.)

[2] L.V. Pisarzhevskii Institute of Physical Chemistry, NAS of Ukraine, Prospekt Nauki 31, 03028 Kyiv, Ukraine

[3] Institute of High Technologies, Taras Shevchenko National University of Kyiv, 60 Volodymyrska Street, 01033 Kyiv, Ukraine; grabchyk@knu.ua

[4] Enamine Scientific Research Institute, 78 Winston Churchill Street, 02094 Kyiv, Ukraine

[5] Institute of Organic Chemistry, NAS of Ukraine, 5 Academik Kukhar Street, 02094 Kyiv, Ukraine

* Correspondence: d.volochnyuk@gmail.com (D.M.V.); s.v.ryabukhin@gmail.com (S.V.R.)

Table of contents

General information and materials S-2

Overview of target products S-3

Analytical data S-4

NMR spectra S-22

General information and materials

The solvents were purified according to the standard procedures. All starting materials were obtained from Enamine Ltd (Kyiv, Ukraine). Melting points were measured on an automated melting point system. ^{1}H and ^{13}C NMR spectra were recorded on a Bruker Avance 500 spectrometer (at 500 MHz for Protons and 126 MHz for Carbon-13) and a Varian Unity Plus 400 spectrometer (at 400 MHz for protons, 101 MHz for Carbon-13). Tetramethylsilane (^{1}H, ^{13}C) was used as standard. HPLC analyses were done on the Agilent 1200 instrument. Mass spectra were recorded on an Agilent 1100 LCMSD SL instrument (chemical ionization (APCI)). Column chromatography was performed using silica gel (200-300 mesh). The high-resolution mass spectrometric analyses (HRMS) were conducted using an Agilent instrument, specifically a hybrid system comprising the 6200 Series Time-of-Flight (TOF) and the 6500 Series Quadrupole Time-of-Flight (Q-TOF). This system was operated with software version B.08.00 (B8058.0). Elemental analyses were performed at the Laboratory of Organic Analysis, Institute of Organic Chemistry, National Academy of Sciences of Ukraine; their results were found to be in good agreement (±0.4%) with the calculated values.

Overview of target products

Figure S1. Target *N*-arylation products for the bromoimidazo[1,5-a]pyridine series. Generic substitution patterns are shown in blue; isolated, structurally confirmed products with preparative yields obtained after scale-up of selected hits on a 250–500 mg bromide scale are shown in black; unconfirmed 1- and 3-substituted targets are shown in gray.

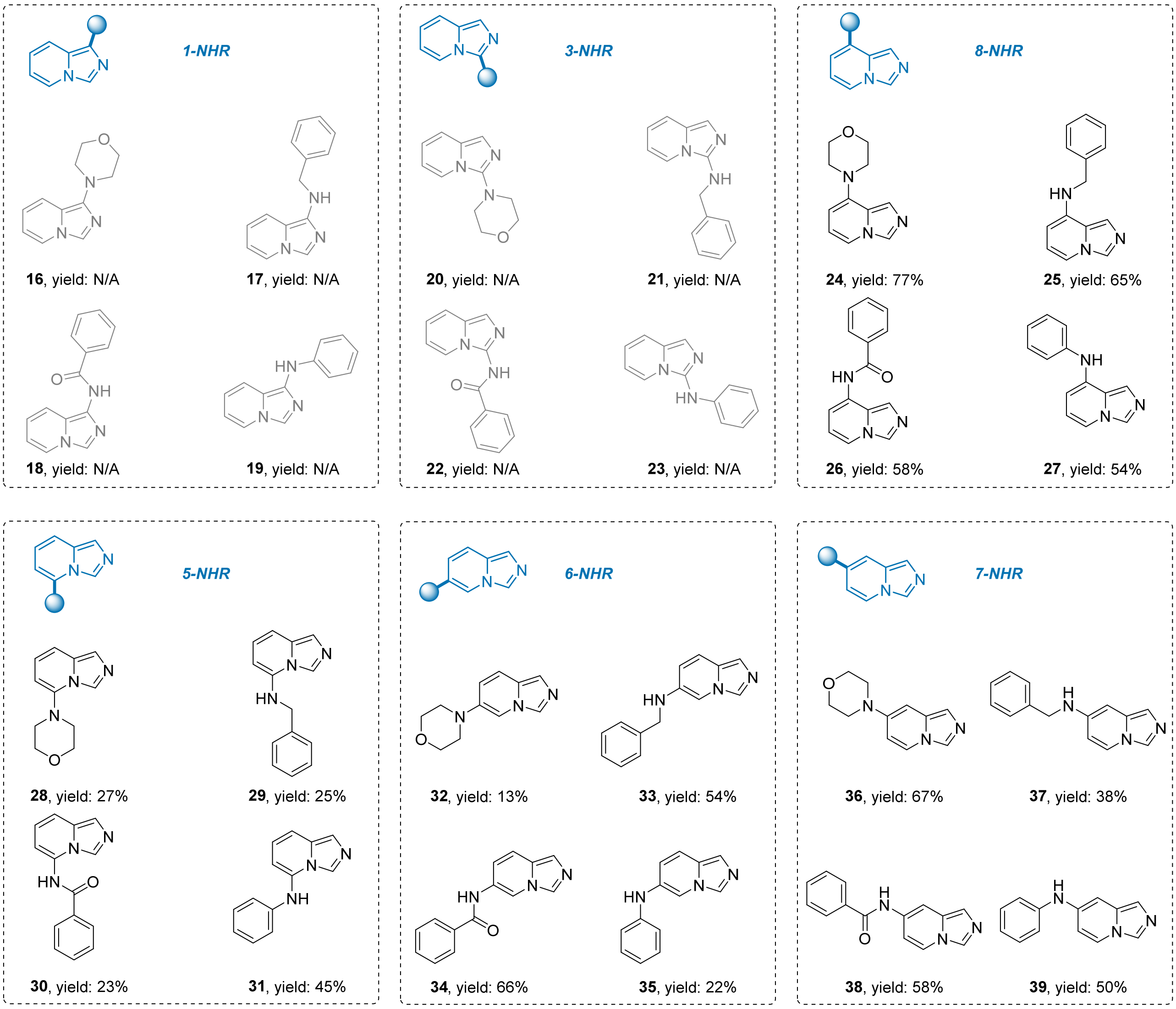

Analytical data

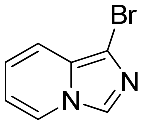

1-bromoimidazo[1,5-a]pyridine (2)

Gray powder. M.p. 78 ºC. ^{1}H NMR (500 MHz, Chloroform-*d*) δ 8.02 (s, 1H), 7.87 (d, *J* = 7.1 Hz, 1H), 7.36 (d, *J* = 9.2 Hz, 1H), 6.76 (dd, *J* = 9.3, 6.4 Hz, 1H), 6.59 (t, *J* = 6.8 Hz, 1H). ^{13}C NMR (101 MHz, Chloroform-*d*) δ 127.9, 126.5, 122.3, 119.8, 117.6, 113.6, 105.0. LCMS, positive mode, m/z: 197.0 [M+H]$^+$. HRMS (ESI): calcd. for $C_7H_6BrN_2$ [M+H]$^+$: 196.9714; found: 196.9709.

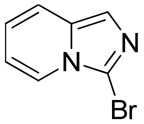

3-bromoimidazo[1,5-a]pyridine (3)

Brown powder. M.p. 44 ºC. ^{1}H NMR (500 MHz, Chloroform-*d*) δ 7.85 (d, *J* = 7.2 Hz, 1H), 7.43 (s, 1H), 7.40 (d, *J* = 9.2 Hz, 1H), 6.75 (dd, *J* = 9.2, 6.4 Hz, 1H), 6.65 (t, *J* = 6.7 Hz, 1H). ^{13}C NMR (101 MHz, Chloroform-*d*) δ 132.7, 121.5, 120.7, 119.1, 118.2, 113.4, 108.2. LCMS, positive mode, m/z: 197.0 [M+H]$^+$. HRMS (ESI): calcd. for $C_7H_6BrN_2$ [M+H]$^+$: 196.9714; found: 196.9708.

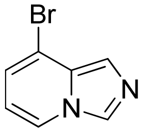

8-bromoimidazo[1,5-a]pyridine (6)

Yellow powder. M.p. 44 ºC. ^{1}H NMR (500 MHz, Chloroform-*d*) δ 8.15 (s, 1H), 7.89 (d, *J* = 7.0 Hz, 1H), 7.50 (s, 1H), 6.92 (d, *J* = 6.9 Hz, 1H), 6.43 (t, *J* = 7.0 Hz, 1H). ^{13}C NMR (126 MHz, Chloroform-*d*) δ 130.1, 129.1, 122.0, 121.6, 121.2, 112.7, 112.4. LCMS, positive mode, m/z: 197.0 [M+H]$^+$. HRMS (ESI): calcd. for $C_7H_6BrN_2$ [M+H]$^+$: 196.9714; found: 196.9707.

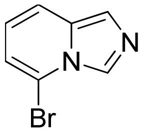

5-bromoimidazo[1,5-a]pyridine (13)

Brown low-melting crystals. ^{1}H NMR (500 MHz, Chloroform-*d*) δ 8.31 (s, 1H), 7.57 (s, 1H), 7.45 (d, *J* = 9.0 Hz, 1H), 6.80 (d, *J* = 6.8 Hz, 1H), 6.62 (dd, *J* = 9.1, 6.9 Hz, 1H). ^{13}C NMR (126 MHz, Chloroform-*d*) δ 132.0, 128.6, 122.1, 119.2, 117.2, 116.4, 111.6. LCMS, positive mode, m/z: 197.0 [M+H]$^+$. HRMS (ESI): calcd. for $C_7H_6BrN_2$ [M+H]$^+$: 196.9714; found: 196.9708.

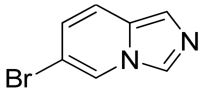

6-bromoimidazo[1,5-a]pyridine (14)

Brown powder. M.p. 92 ºC. ^{1}H NMR (500 MHz, Chloroform-*d*) δ 8.08 (d, *J* = 1.6 Hz, 1H), 8.05 (s, 1H), 7.43 (s, 1H), 7.33 (d, *J* = 9.5 Hz, 1H), 6.74 (dd, *J* = 9.6, 1.5 Hz, 1H). ^{13}C NMR (101 MHz, Chloroform-*d*) δ 128.6, 127.6, 122.7, 122.0, 121.2, 118.9, 108.2. LCMS, positive mode, m/z: 197.0 [M+H]$^+$. HRMS (ESI): calcd. for $C_7H_6BrN_2$ [M+H]$^+$: 196.9714; found: 196.9709.

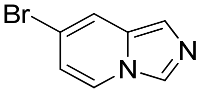

7-bromoimidazo[1,5-a]pyridine (15)

Beige powder. M.p. 75 ºC. ^{1}H NMR (500 MHz, Chloroform-*d*) δ 8.09 (s, 1H), 7.79 (d, *J* = 7.4 Hz, 1H), 7.61 (s, 1H), 7.36 (s, 1H), 6.60 (dd, *J* = 7.4, 1.9 Hz, 1H). ^{13}C NMR (101 MHz, Chloroform-*d*) δ 130.6, 128.0, 122.6, 120.3, 119.7, 116.6, 112.3. LCMS, positive mode, m/z: 197.0 [M+H]$^{+}$. HRMS (ESI): calcd. for $C_7H_6BrN_2$ [M+H]$^{+}$: 196.9714; found: 196.9707.

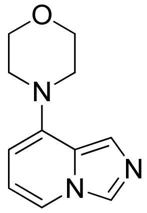

4-(imidazo[1,5-a]pyridin-8-yl)morpholine (24). Conditions (ligand / base): IPent·HCl / Cs$_2$CO$_3$

Beige powder. Yield 77%. M.p. 80 ºC. ^{1}H NMR (500 MHz, DMSO-*d*$_6$) δ 8.33 (s, 1H), 7.99 (d, *J* = 6.8 Hz, 1H), 7.37 (s, 1H), 6.56 (t, *J* = 7.0 Hz, 1H), 6.09 (d, *J* = 7.1 Hz, 1H), 3.79 (t, *J* = 4.7 Hz, 4H), 3.12 (t, *J* = 4.7 Hz, 4H). ^{13}C NMR (126 MHz, DMSO-*d*$_6$) δ 142.7, 129.6, 126.6, 119.4, 117.5, 113.1, 102.6, 66.6, 50.8. LCMS, positive mode, m/z: 204.2 [M+H]$^{+}$. HRMS (ESI): calcd. for $C_{11}H_{14}N_3O$ [M+H]$^{+}$: 204.1132; found 204.1137.

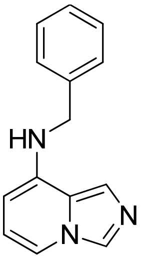

N-benzylimidazo[1,5-a]pyridin-8-amine (25). Conditions (ligand / base): BINAP / Cs$_2$CO$_3$

Gray powder. Yield 65%. ^{1}H NMR (500 MHz, DMSO-*d*$_6$) δ 8.20 (s, 1H), 7.60 (d, *J* = 6.7 Hz, 1H), 7.56 (s, 1H), 7.38 – 7.33 (m, 2H), 7.30 (t, *J* = 7.5 Hz, 2H), 7.20 (t, *J* = 7.5 Hz, 1H), 6.89 (t, *J* = 6.1 Hz, 1H), 6.37 (t, *J* = 7.0 Hz, 1H), 5.48 (d, *J* = 7.1 Hz, 1H), 4.40 (d, *J* = 5.8 Hz, 2H). ^{13}C NMR (126 MHz, DMSO-*d*$_6$) δ 140.0, 138.3, 129.3, 128.8, 127.4, 127.2, 125.5, 118.1, 114.2, 111.9, 92.0, 46.3. LCMS, positive mode, m/z: 224.2 [M+H]$^{+}$. HRMS (ESI): calcd. for $C_{14}H_{14}N_3$ [M+H]$^{+}$: 224.1182; found 224.1189.

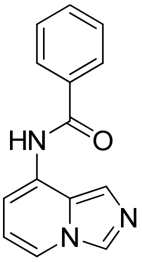

N-(imidazo[1,5-a]pyridin-8-yl)benzamide (26)). Conditions (ligand / base): XantPhos / tBuONa

Beige powder. Yield 58%. ^{1}H NMR (400 MHz, DMSO-*d*$_6$) δ 10.24 (s, 1H), 8.41 (d, *J* = 2.1 Hz, 1H), 8.19 (d, *J* = 6.9 Hz, 1H), 7.97 (dd, *J* = 7.3, 2.5 Hz, 2H), 7.67 – 7.58 (m, 2H), 7.55 (t, *J* = 7.3 Hz, 2H), 7.30 (dd, *J* = 7.2, 2.1 Hz, 1H), 6.69 (td, *J* = 7.0, 2.2 Hz, 1H). ^{13}C NMR (101 MHz, Chloroform-*d*) δ 166.2, 134.3, 132.3, 129.0, 129.0, 128.1, 127.1, 125.7, 118.2, 116.4, 113.4, 108.3. LCMS, positive mode, m/z: 238.2 [M+H]$^{+}$. HRMS (ESI): calcd. for $C_{14}H_{12}N_3O$ [M+H]$^{+}$: 238.0975; found 238.0980.

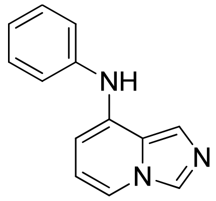

***N*-phenylimidazo[1,5-a]pyridin-8-amine (27). Conditions (ligand / base): IPent·HCl / Cs_2CO_3**

Brown powder. Yield 54%. ^{1}H NMR (500 MHz, DMSO-d_6) δ 8.32 (s, 1H), 8.25 (s, 1H), 7.86 (d, J = 6.8 Hz, 1H), 7.58 (s, 1H), 7.32 (t, J = 7.7 Hz, 2H), 7.24 (d, J = 7.9 Hz, 2H), 6.97 (t, J = 7.3 Hz, 1H), 6.52 (t, J = 7.0 Hz, 1H), 6.36 (d, J = 7.2 Hz, 1H). ^{13}C NMR (101 MHz, DMSO-d_6) δ 142.2, 134.4, 129.6, 129.6, 126.0, 122.0, 120.3, 118.6, 114.9, 113.6, 97.2. LCMS, positive mode, m/z: 210.2 $[M+H]^+$. HRMS (ESI): calcd. for $C_{13}H_{12}N_3$ $[M+H]^+$: 210.1026; found 210.1033.

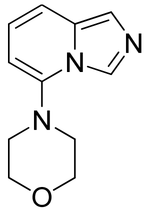

4-(imidazo[1,5-a]pyridin-5-yl)morpholine (28). Conditions (ligand / base): IPent·HCl / tBuONa

Yellow powder. Yield 27%. M.p. 100 ºC. ^{1}H NMR (500 MHz, DMSO-d_6) δ 8.28 (s, 1H), 7.39 (s, 1H), 7.30 (d, J = 9.0 Hz, 1H), 6.80 (dd, J = 9.0, 6.8 Hz, 1H), 6.19 (d, J = 6.8 Hz, 1H), 3.82 (t, J = 4.6 Hz, 4H), 3.05 (t, J = 4.6 Hz, 4H). ^{13}C NMR (151 MHz, Chloroform-d) δ 142.5, 131.6, 125.0, 120.7, 119.9, 113.4, 99.0, 66.8, 50.2. LCMS, positive mode, m/z: 204.2 $[M+H]^+$. HRMS (ESI): calcd. for $C_{11}H_{14}N_3O$ $[M+H]^+$: 204.1132; found 204.1139.

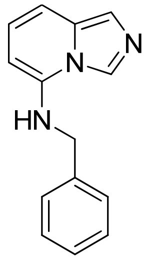

***N*-benzylimidazo[1,5-a]pyridin-5-amine (29). Conditions (ligand / base): BINAP / Cs_2CO_3**

Brown oil. Yield 25%. ^{1}H NMR (400 MHz, DMSO-d_6) δ 8.47 (s, 1H), 7.47 – 7.39 (m, 2H), 7.39 – 7.29 (m, 3H), 7.30 – 7.22 (m, 2H), 6.88 (d, J = 8.9 Hz, 1H), 6.69 (t, J = 8.0 Hz, 1H), 5.54 (d, J = 7.1 Hz, 1H), 4.52 (d, J = 5.6 Hz, 2H). ^{13}C NMR (126 MHz, Chloroform-d) δ 138.9, 137.8, 131.9, 128.8, 127.6, 127.4, 122.9, 121.7, 118.7, 106.6, 88.6, 47.3. LCMS, positive mode, m/z: 224.2 $[M+H]^+$. HRMS (ESI): calcd. for $C_{14}H_{14}N_3$ $[M+H]^+$: 224.1182; found 224.1188.

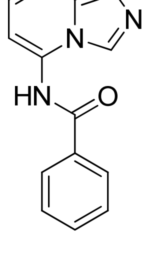

***N*-(imidazo[1,5-a]pyridin-5-yl)benzamide (30). Conditions (ligand / base): XantPhos / tBuONa**

Beige powder.. Yield 23%. ^{1}H NMR (500 MHz, DMSO-d_6) δ 10.85 (s, 1H), 8.23 (s, 1H), 8.04 (d, J = 7.6 Hz, 2H), 7.65 (t, J = 7.4 Hz, 1H), 7.57 (t, J = 7.6 Hz, 2H), 7.52 (d, J = 9.0 Hz, 1H), 7.46 (s, 1H), 6.86 (dd, J = 9.1, 6.7 Hz, 1H), 6.75 (d, J = 6.8 Hz, 1H). ^{13}C NMR (126 MHz, Chloroform-d) δ 132.8, 129.0, 127.6, 125.2, 124.5, 122.8, 121.0, 119.5, 117.1, 116.0, 107.8, 104.2. LCMS, positive mode,

m/z: 238.2 $[M+H]^+$. Anal. calcd. for $C_{14}H_{11}N_3O$: C, 70.87; H, 4.67; N, 17.71. Found: C, 71.26; H, 5.07; N, 17.77.

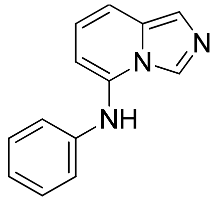

N-phenylimidazo[1,5-a]pyridin-5-amine (31). Conditions (ligand / base): XantPhos / Cs_2CO_3

Brown oil. Yield 45%. ^{1}H NMR (400 MHz, DMSO-d_6) δ 8.60 (s, 1H), 8.39 (s, 1H), 7.45 – 7.29 (m, 3H), 7.18 (t, J = 7.3 Hz, 3H), 7.04 (t, J = 7.4 Hz, 1H), 6.79 (t, J = 8.1 Hz, 1H), 6.22 (dd, J = 7.1, 2.8 Hz, 1H). ^{13}C NMR (101 MHz, Chloroform-d) δ 141.0, 134.8, 131.9, 129.6, 124.2, 122.8, 120.3, 120.2, 119.2, 111.8, 98.7. LCMS, positive mode, m/z: 210.2 $[M+H]^+$. HRMS (ESI): calcd. for $C_{13}H_{12}N_3$ $[M+H]^+$: 210.1026; found 210.1033.

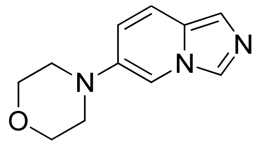

4-(imidazo[1,5-a]pyridin-6-yl)morpholine (32). Conditions (ligand / base): IPent·HCl / tBuONa

Beige powder. Yield 13%. M.p. 65 ºC. ^{1}H NMR (500 MHz, DMSO-d_6) δ 8.19 (s, 1H), 7.73 (s, 1H), 7.44 (d, J = 9.7 Hz, 1H), 7.22 (s, 1H), 6.81 (dd, J = 9.8, 2.0 Hz, 1H), 3.74 (t, J = 4.7 Hz, 4H), 2.98 (t, J = 4.7 Hz, 4H). ^{13}C NMR (126 MHz, Chloroform-d) δ 139.9, 128.1, 127.9, 120.2, 118.3, 116.6, 106.5, 66.7, 50.4. LCMS, positive mode, m/z: 204.2 $[M+H]^+$. HRMS (ESI): calcd. for $C_{11}H_{14}N_3O$ $[M+H]^+$: 204.1131; found 204.1138.

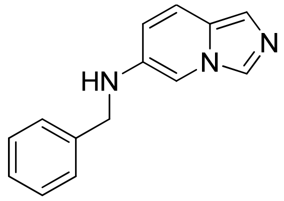

N-benzylimidazo[1,5-a]pyridin-6-amine (33). Conditions (ligand / base):BINAP / tBuONa

Gray powder. Yield 54%. ^{1}H NMR (400 MHz, DMSO-d_6) δ 8.04 (s, 1H), 7.44 – 7.37 (m, 2H), 7.35 (t, J = 8.5 Hz, 3H), 7.30 – 7.21 (m, 2H), 7.14 (s, 1H), 6.56 (dd, J = 9.6, 2.0 Hz, 1H), 6.03 (t, J = 5.7 Hz, 1H), 4.18 (d, J = 5.7 Hz, 2H). ^{13}C NMR (101 MHz, DMSO-d_6) δ 139.6, 136.8, 128.8, 127.9, 127.3, 127.2, 119.5, 117.9, 117.0, 99.5, 47.3. LCMS, positive mode, m/z: 224.2 $[M+H]^+$. HRMS (ESI): calcd. for $C_{14}H_{12}N_3O$ $[M+H]^+$: 224.1182; found 224.1187.

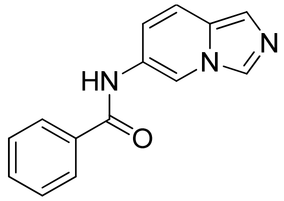

N-(imidazo[1,5-a]pyridin-6-yl)benzamide (34). Conditions (ligand / base): tBuBrettPhos / Cs_2CO_3

Beige powder. Yield 66%. ^{1}H NMR (500 MHz, DMSO-d_6) δ 10.23 (s, 1H), 9.19 (d, J = 4.0 Hz, 1H), 8.42 (s, 1H), 7.95 (d, J = 7.2 Hz, 2H), 7.60 (t, J = 7.3 Hz, 1H), 7.57 – 7.48 (m, 3H), 7.31 (s, 1H), 6.98 (dt, J = 9.6, 2.0 Hz, 1H). ^{13}C NMR (101 MHz, Chloroform-d) δ 166.1, 134.1, 132.2, 128.9, 128.8, 128.3, 127.1, 125.6, 120.4, 118.5, 115.9, 113.2. LCMS, positive mode, m/z: 238.2 $[M+H]^+$. HRMS (ESI): calcd. for $C_{14}H_{14}N_3$ $[M+H]^+$: 238.0975; found 238.0980.

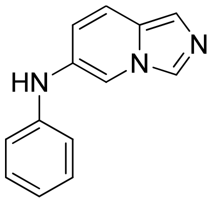

N-phenylimidazo[1,5-a]pyridin-6-amine (35). Conditions (ligand / base): XantPhos / tBuONa
Dark green powder. Yield 22%. ^{1}H NMR (500 MHz, DMSO-d_6) δ 8.24 (s, 1H), 8.12 (s, 1H), 7.98 (s, 1H), 7.49 (d, *J* = 9.6 Hz, 1H), 7.25 (dd, *J* = 13.7, 5.9 Hz, 3H), 7.03 (d, *J* = 7.9 Hz, 2H), 6.82 (t, *J* = 7.3 Hz, 1H), 6.66 (dd, *J* = 9.7, 1.8 Hz, 1H). ^{13}C NMR (101 MHz, DMSO-d_6) δ 144.2, 130.8, 129.7, 128.7, 127.9, 120.0, 119.8, 118.4, 118.4, 116.8, 108.3. LCMS, positive mode, m/z: 210.2 $[M+H]^+$. HRMS (ESI): calcd. for $C_{13}H_{12}N_3$ $[M+H]^+$: 210.1026; found 210.1033.

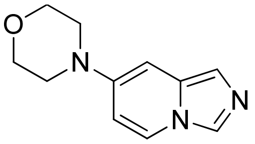

4-(imidazo[1,5-a]pyridin-7-yl)morpholine (36). Conditions (ligand / base): XantPhos / tBuONa
Black oil. Yield 67%. ^{1}H NMR (400 MHz, DMSO-d_6) δ 8.19 (dd, *J* = 7.8, 3.4 Hz, 1H), 8.10 (d, *J* = 3.0 Hz, 1H), 7.00 (d, *J* = 3.0 Hz, 1H), 6.71 – 6.61 (m, 1H), 6.62 – 6.50 (m, 1H), 3.87 – 3.56 (m, 4H), 3.14 – 2.92 (m, 4H). ^{13}C NMR (126 MHz, Chloroform-*d*) δ 143.8, 131.2, 126.3, 122.8, 117.1, 108.6, 97.5, 66.6, 49.4. LCMS, positive mode, m/z: 204.0 $[M+H]^+$. HRMS (ESI): calcd. for $C_{11}H_{14}N_3O$ $[M+H]^+$: 204.1131; found 204.1137.

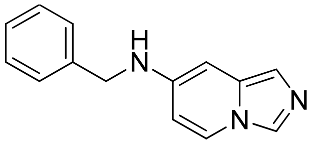

N-benzylimidazo[1,5-a]pyridin-7-amine (37). Conditions (ligand / base): XantPhos / Cs_2CO_3
Black powder. Yield 38%. ^{1}H NMR (500 MHz, DMSO-d_6) δ 8.06 (d, *J* = 7.5 Hz, 1H), 7.94 (s, 1H), 7.44 – 7.29 (m, 4H), 7.24 (t, *J* = 7.2 Hz, 1H), 6.72 (s, 1H), 6.47 (t, *J* = 5.8 Hz, 1H), 6.33 (dd, *J* = 7.5, 2.1 Hz, 1H), 6.03 (s, 1H), 4.24 (d, *J* = 5.7 Hz, 2H). ^{13}C NMR (101 MHz, Chloroform-*d*) δ 140.2, 138.2, 132.0, 128.8, 127.6, 127.5, 125.8, 122.9, 115.3, 108.8, 90.5, 48.0. LCMS, positive mode, m/z: 224.2 $[M+H]^+$. HRMS (ESI): calcd. for $C_{14}H_{14}N_3$ $[M+H]^+$: 224.1182; found 224.1188.

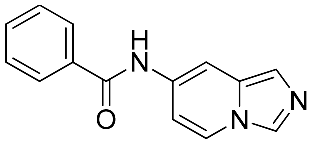

N-(imidazo[1,5-a]pyridin-7-yl)benzamide (38). Conditions (ligand / base): dppf / Cs_2CO_3
Beige powder. Yield 58%. ^{1}H NMR (500 MHz, DMSO-d_6) δ 10.28 (s, 1H), 8.30 (d, *J* = 7.6 Hz, 1H), 8.26 (s, 1H), 8.13 (t, *J* = 2.8 Hz, 1H), 7.93 (d, *J* = 7.5 Hz, 2H), 7.59 (t, *J* = 7.3 Hz, 1H), 7.53 (t, *J* = 7.5 Hz, 2H), 7.24 (s, 1H), 6.95 (dt, *J* = 7.6, 2.3 Hz, 1H). ^{13}C NMR (151 MHz, DMSO-d_6) δ 166.4, 135.1, 132.2, 131.4, 130.1, 128.9, 128.2, 128.1, 123.9, 118.8, 109.4, 104.6. LCMS, positive mode, m/z: 238.2 $[M+H]^+$. HRMS (ESI): calcd. for $C_{14}H_{12}N_3O$ $[M+H]^+$: 238.0975; found 238.0982.

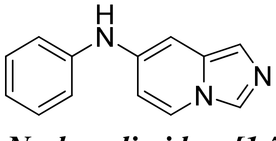

N-phenylimidazo[1,5-a]pyridin-7-amine (39). Conditions (ligand / base): RuPhos / Cs_2CO_3
Dark green powder. Yield 50%. ^{1}H NMR (400 MHz, DMSO-d_6) δ 8.29 (s, 1H), 8.22 (d, *J* = 7.5 Hz, 1H), 8.14 (s, 1H), 7.29 (t, *J* = 7.9 Hz, 2H), 7.14 (d, *J* = 7.8 Hz, 2H), 6.96 (d, *J* = 12.1 Hz, 2H), 6.90 (t, *J* = 7.5 Hz, 1H), 6.48 (dd, *J* = 7.5, 2.3 Hz, 1H). ^{13}C NMR (101 MHz, DMSO-d_6) δ 142.8, 136.3,

131.2, 129.7, 127.2, 124.5, 121.0, 118.4, 115.8, 109.6, 94.7, 79.6. LCMS, positive mode, m/z: 210.2 $[M+H]^+$. HRMS (ESI): calcd. for $C_{13}H_{12}N_3$ $[M+H]^+$: 210.1026; found 210.1033.

Ligand structures and products distributions

Figure S2. Structures of the ligands and the Pd precatalyst included in the Buchwald–Hartwig C-N coupling screen.

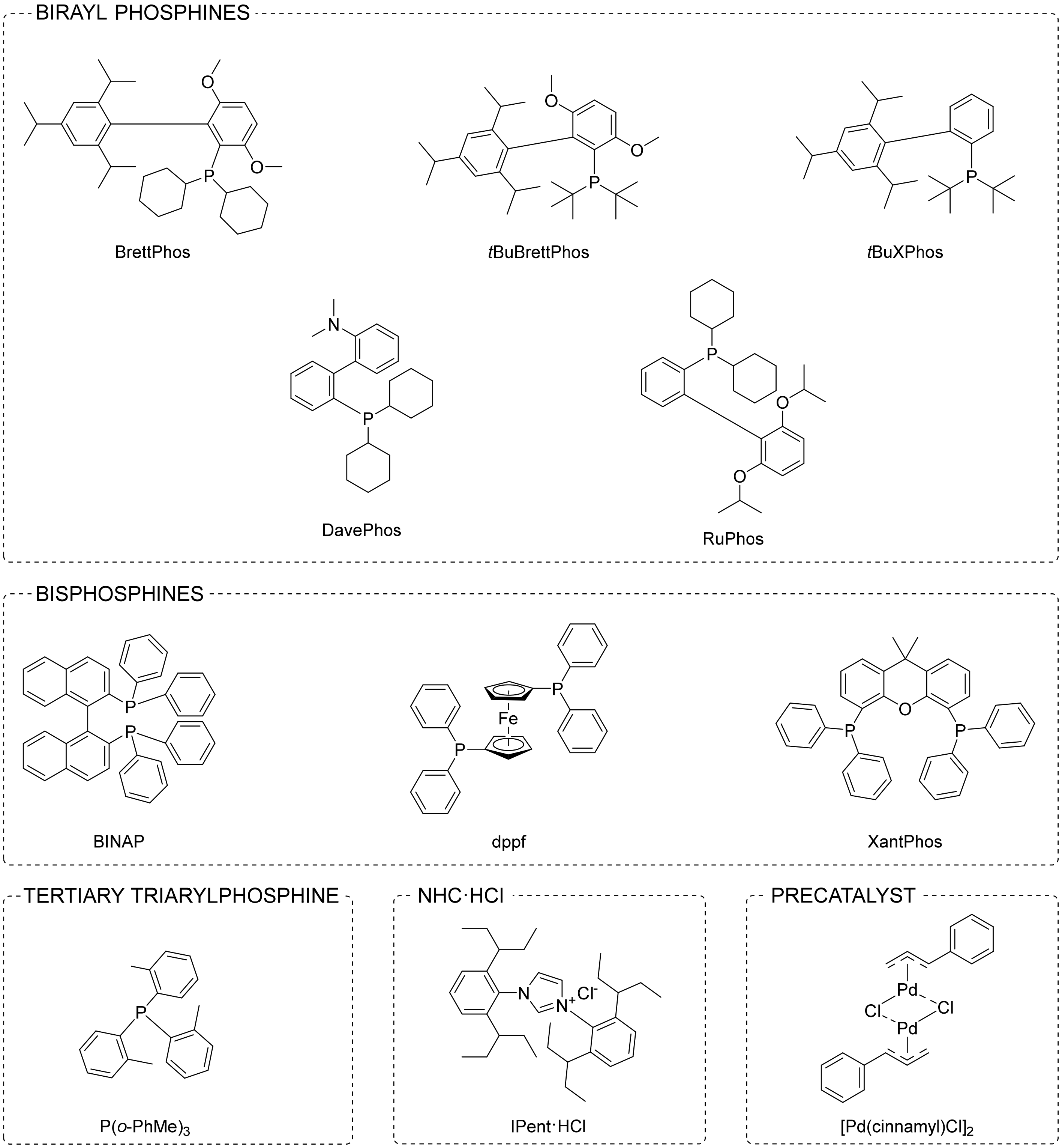

Figure S3. Reaction–mixture composition matrix for 1-bromoimidazo[1,5-a]pyridine (1-Br-ImPy, **2**) across the screened ligand/base/nucleophile combinations according to LCMS data.

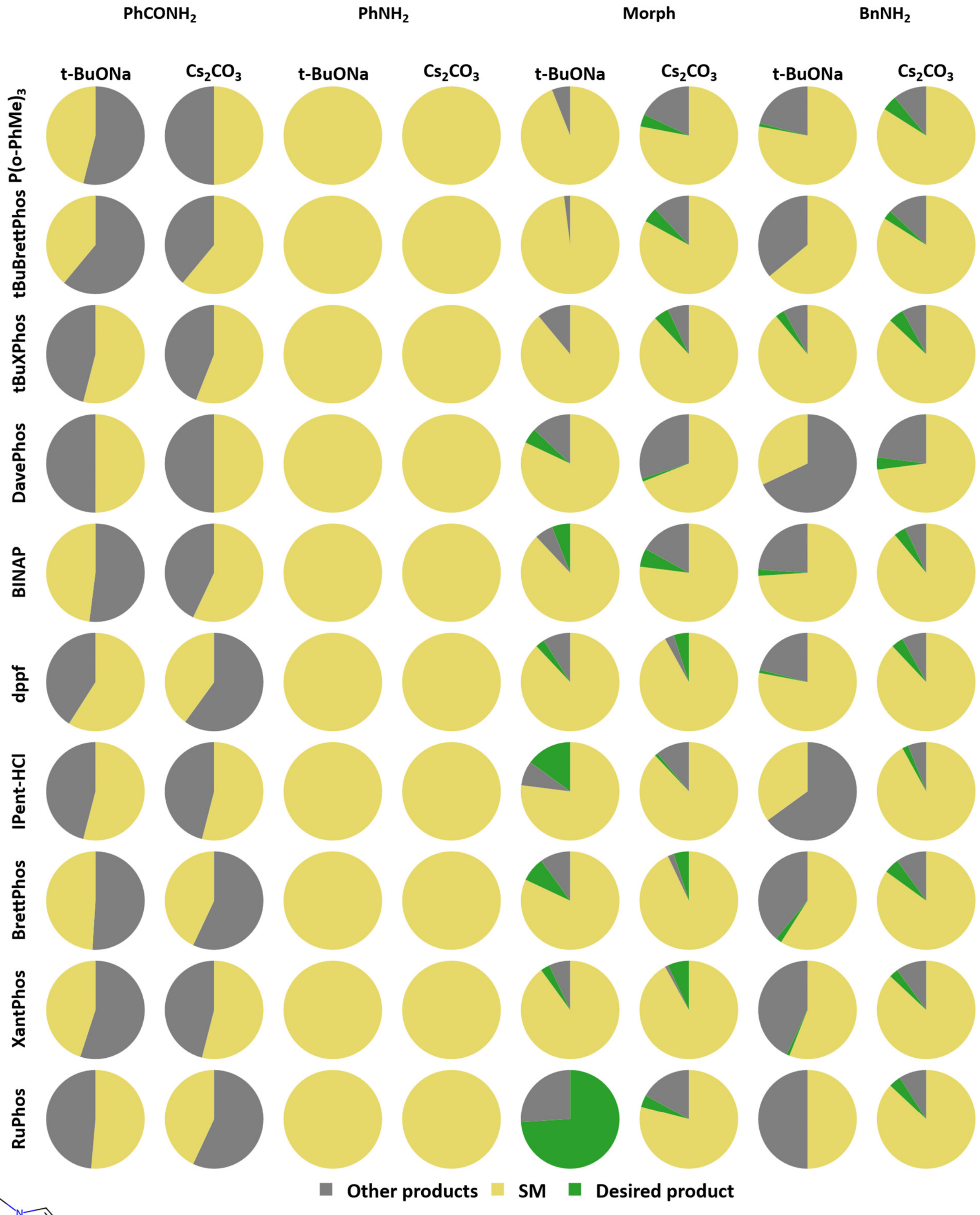

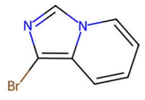

1-Br-ImPy (2)

Figure S4. Reaction–mixture composition matrix for 3-bromoimidazo[1,5-a]pyridine (3-Br-ImPy, **3**) across the screened ligand/base/nucleophile combinations according to LCMS data.

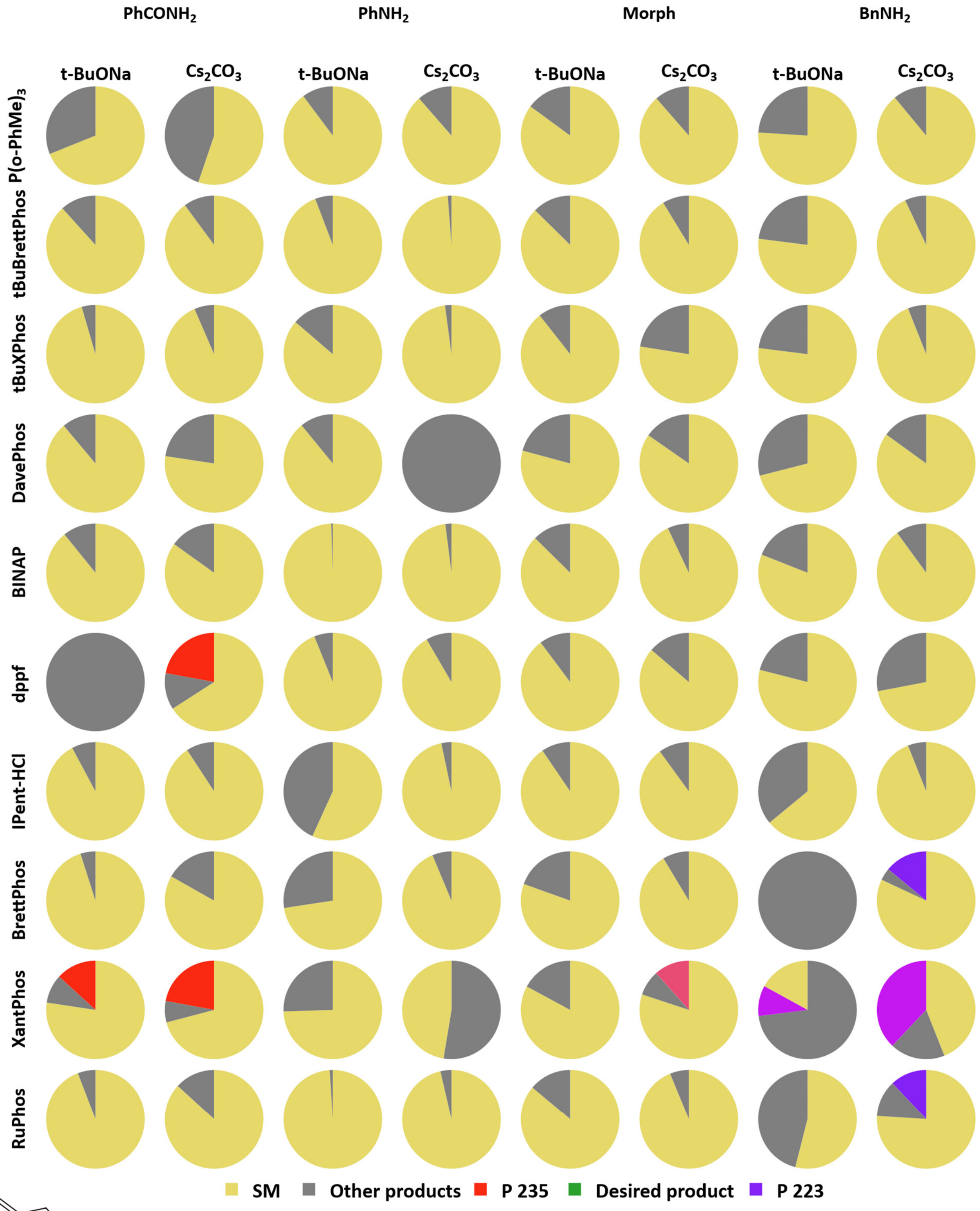

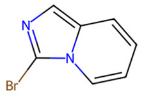

3-Br-ImPy (3)

Figure S5. Reaction–mixture composition matrix for 5-bromoimidazo[1,5-a]pyridine (5-Br-ImPy, **13**) across the screened ligand/base/nucleophile combinations according to LCMS data.

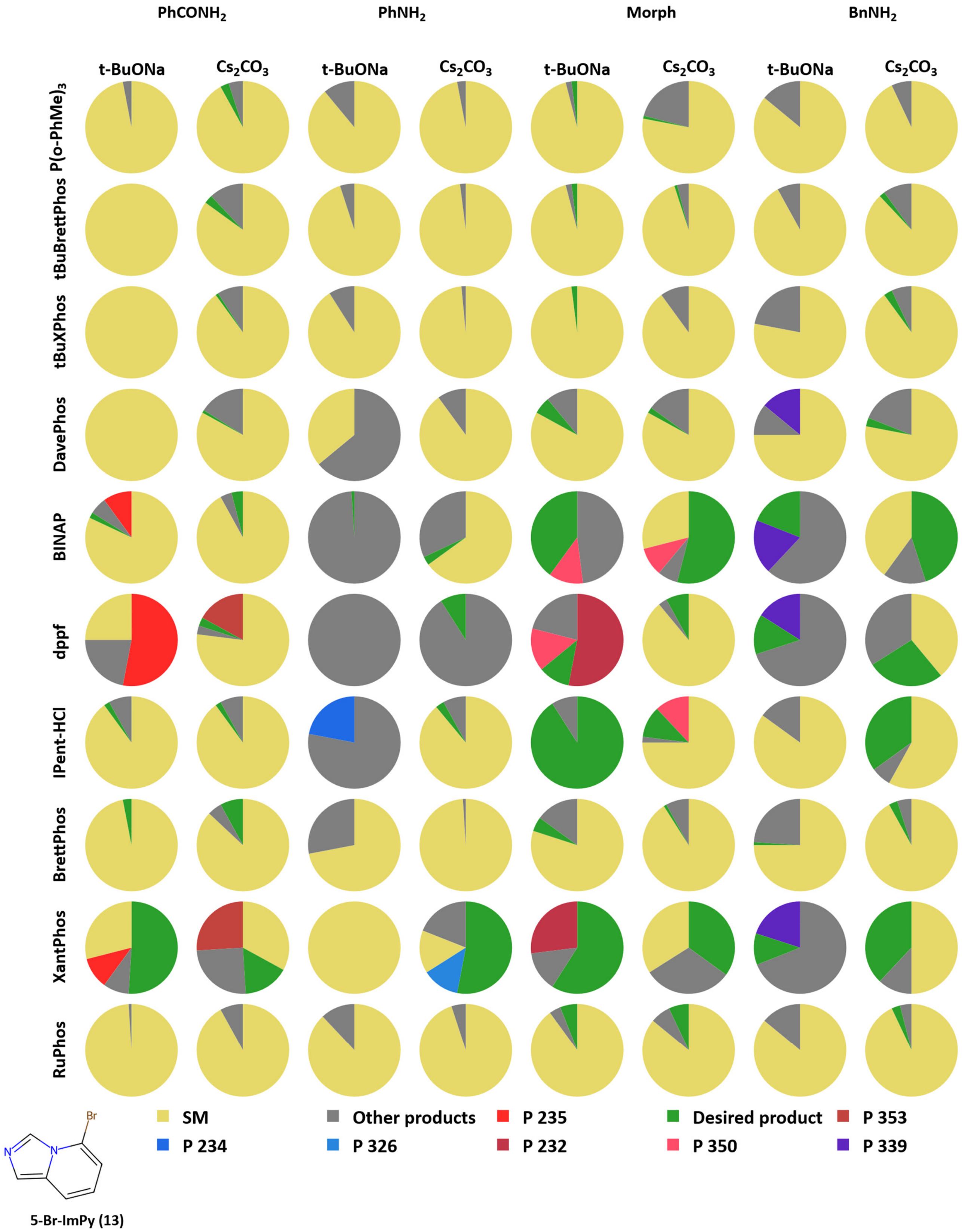

Figure S6. Reaction–mixture composition matrix for 6-bromoimidazo[1,5-a]pyridine (6-Br-ImPy, **14**) across the screened ligand/base/nucleophile combinations according to LCMS data.

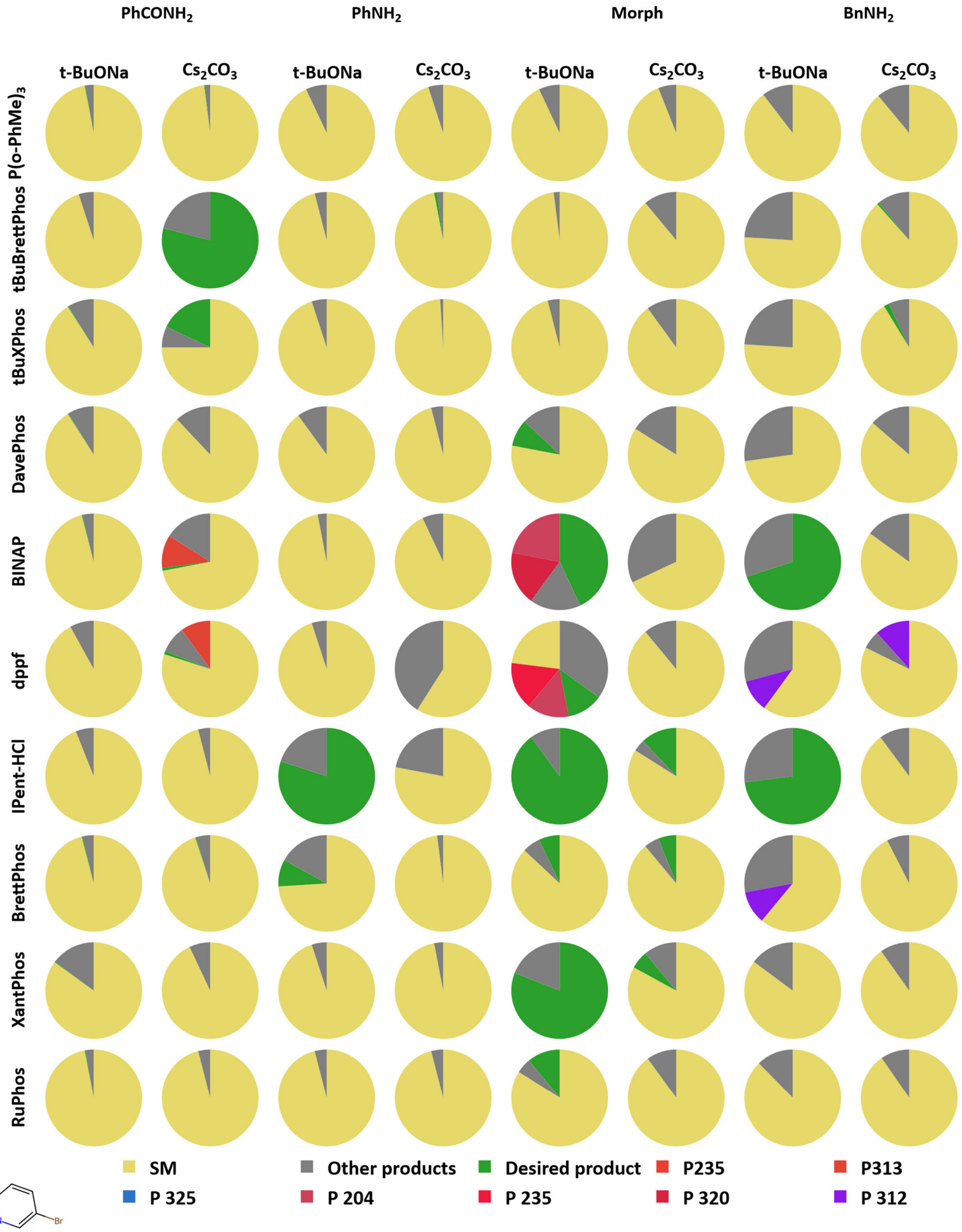

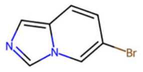

6-Br-ImPy (14)

Figure S7. Reaction–mixture composition matrix for 7-bromoimidazo[1,5-a]pyridine (7-Br-ImPy, **15**) across the screened ligand/base/nucleophile combinations according to LCMS data.

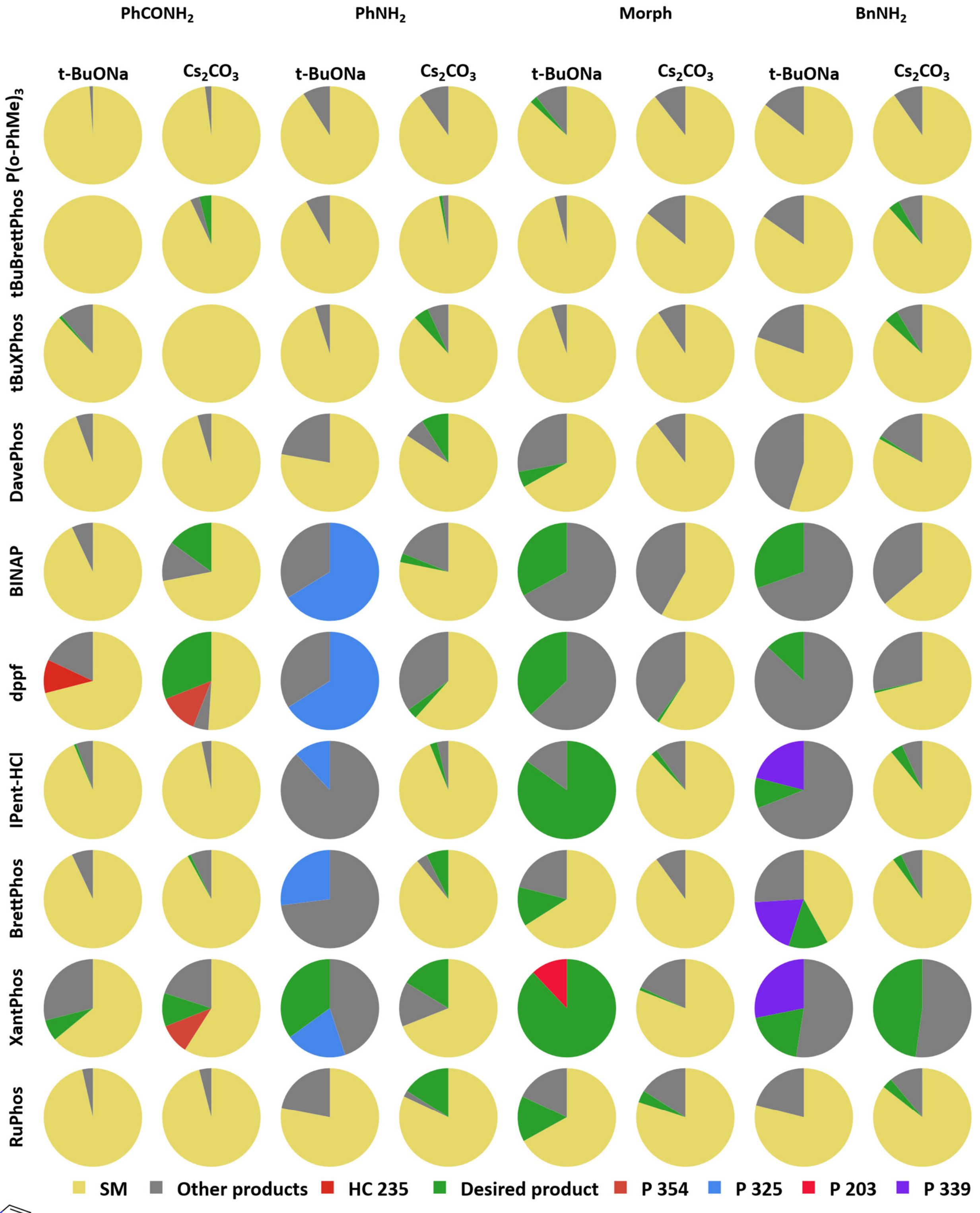

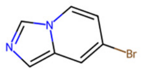

7-Br-ImPy (15)

Figure S8. Reaction–mixture composition matrix for 8-bromoimidazo[1,5-a]pyridine (8-Br-ImPy, **6**) across the screened ligand/base/nucleophile combinations according to LCMS data.

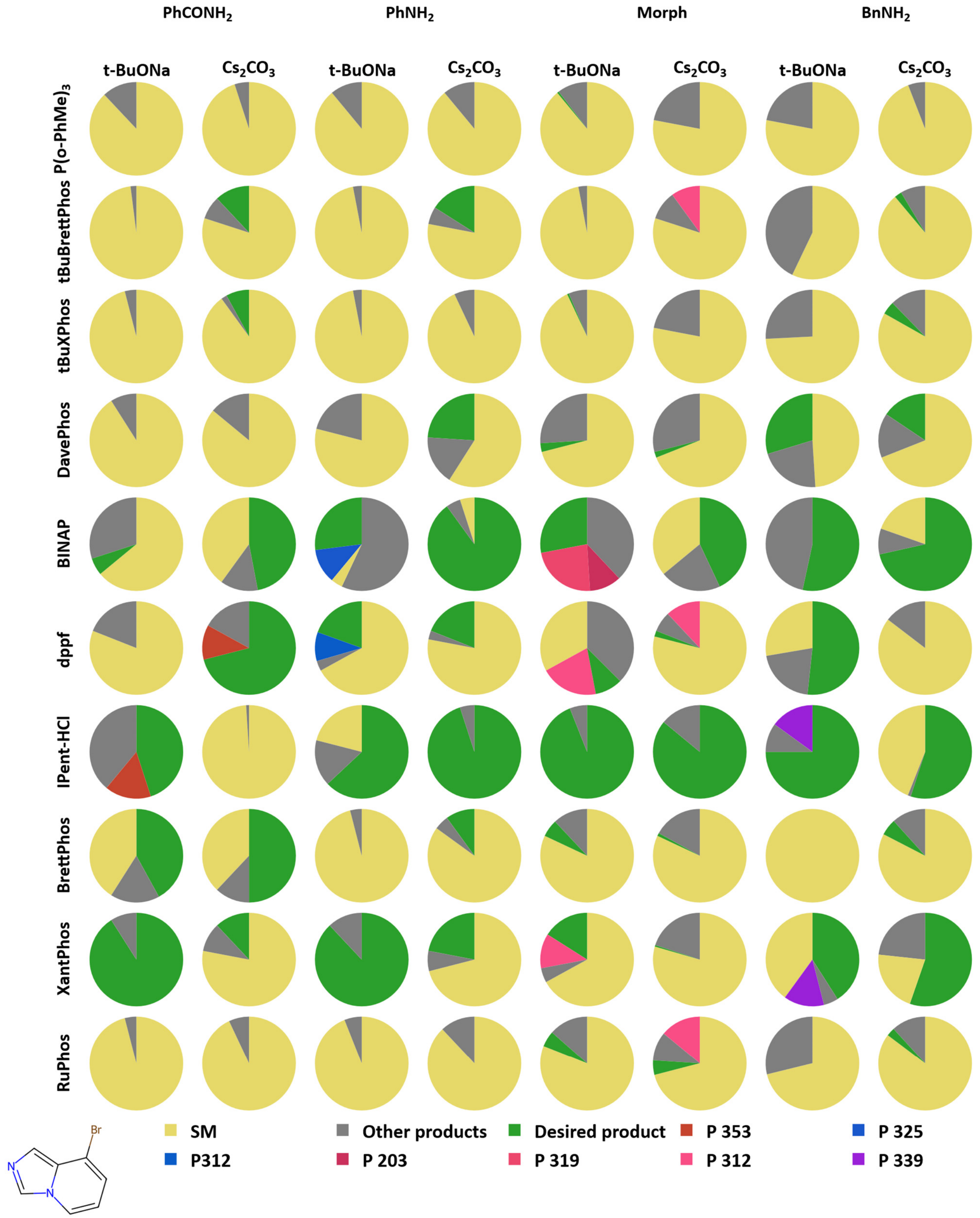

8-Br-ImPy (6)

Table S1. LCMS-based conversion (%) of the bromoimidazo[1,5-a]pyridines under the screened ligand/base/nucleophile combinations. Conversion values were calculated from residual starting bromide signals normalized to the post-added naphthalene internal standard.

**1-bromoimidazo[1,5-a]pyridine (2)**								
	$PhCONH_2$	$PhCONH_2$	$PhNH_2$	$PhNH_2$	Morph	Morph	$BnNH_2$	$BnNH_2$
	t-BuONa	Cs_2CO_3	*t*-BuONa	Cs_2CO_3	*t*-BuONa	Cs_2CO_3	*t*-BuONa	Cs_2CO_3
RuPhos	33	58	22	27	100	0	68	15
XantPhos	42	34	28	19	0	0	54	14
BrettPhos	43	53	2	34	17	0	60	18
IPent-HCl	28	31	51	40	43	0	74	18
dppf	27	69	50	37	58	40	54	45
BINAP	46	31	15	32	0	0	41	13
DavePhos	42	36	49	10	23	0	83	17
*t*BuXPhos	36	28	65	33	0	0	13	22
*t*BuBrettPhos	65	27	38	29	17	0	66	30
P(*o*-PhMe)$_3$	38	28	25	15	0	0	34	14
**3-bromoimidazo[1,5-a]pyridine (3)**								
	$PhCONH_2$	$PhCONH_2$	$PhNH_2$	$PhNH_2$	Morph	Morph	$BnNH_2$	$BnNH_2$
	t-BuONa	Cs_2CO_3	*t*-BuONa	Cs_2CO_3	*t*-BuONa	Cs_2CO_3	*t*-BuONa	Cs_2CO_3
RuPhos	50	50	63	43	51	48	82	18
XantPhos	68	70	86	68	54	58	97	66
BrettPhos	44	56	86	61	68	47	100	3
IPent-HCl	44	50	55	50	45	61	88	54
dppf	100	83	72	73	63	67	61	54
BINAP	45	45	49	51	53	61	65	50
DavePhos	44	58	54	100	46	56	69	47
*t*BuXPhos	44	47	77	34	56	69	82	52
*t*BuBrettPhos	61	46	73	53	60	46	81	50
P(*o*-PhMe)$_3$	42	61	52	45	50	49	58	45

**5-bromoimidazo[1,5-a]pyridine (13)**								
	$PhCONH_2$	$PhCONH_2$	$PhNH_2$	$PhNH_2$	Morph	Morph	$BnNH_2$	$BnNH_2$
	t-BuONa	Cs_2CO_3	*t*-BuONa	Cs_2CO_3	*t*-BuONa	Cs_2CO_3	*t*-BuONa	Cs_2CO_3
RuPhos	0	0	36	0	20	2	25	4
XantPhos	65	59	100	88	100	67	100	46
BrettPhos	1	0	63	0	42	0	53	0
IPent-HCl	10	0	100	32	100	25	44	44
dppf	89	8	100	100	100	11	100	36
BINAP	14	0	100	53	100	73	100	53
DavePhos	0	0	84	0	16	8	39	16
*t*BuXPhos	0	0	0	0	12	0	43	7
*t*BuBrettPhos	0	18	4	0	14	0	55	51
P(*o*-PhMe)$_3$	0	18	6	0	4	47	41	0

**6-bromoimidazo[1,5-a]pyridine (14)**								
	$PhCONH_2$	$PhCONH_2$	$PhNH_2$	$PhNH_2$	Morph	Morph	$BnNH_2$	$BnNH_2$
	t-BuONa	Cs_2CO_3	*t*-BuONa	Cs_2CO_3	*t*-BuONa	Cs_2CO_3	*t*-BuONa	Cs_2CO_3
RuPhos	30	38	37	39	39	0	44	15
XantPhos	45	42	35	29	100	18	35	20
BrettPhos	24	36	34	23	66	1	82	15
IPent-HCl	22	30	100	67	100	39	100	28
dppf	32	57	40	34	95	0	90	54
BINAP	29	76	28	44	100	56	100	30
DavePhos	21	48	23	38	50	8	67	24
*t*BuXPhos	23	72	19	16	36	23	31	22
*t*BuBrettPhos	27	100	20	31	12	50	47	25
P(*o*-PhMe)$_3$	23	24	24	34	16	0	25	23

7-bromoimidazo[1,5-a]pyridine (15)								
	$PhCONH_2$	$PhCONH_2$	$PhNH_2$	$PhNH_2$	Morph	Morph	$BnNH_2$	$BnNH_2$
	t-BuONa	Cs_2CO_3	*t*-BuONa	Cs_2CO_3	*t*-BuONa	Cs_2CO_3	*t*-BuONa	Cs_2CO_3
RuPhos	37	41	72	53	84	45	78	48
XantPhos	74	54	100	73	100	54	100	100
BrettPhos	35	54	100	52	83	39	87	37
IPent-HCl	48	46	100	49	100	54	100	47
dppf	70	70	100	71	100	72	100	73
BINAP	32	30	100	67	100	65	100	69
DavePhos	26	30	64	59	80	61	80	51
*t*BuXPhos	53	0	50	58	68	60	69	48
*t*BuBrettPhos	51	5	59	50	64	67	81	58
P(*o*-PhMe)$_3$	19	28	53	54	76	85	76	59
**8-bromoimidazo[1,5-a]pyridine (6)**								
	$PhCONH_2$	$PhCONH_2$	$PhNH_2$	$PhNH_2$	Morph	Morph	$BnNH_2$	$BnNH_2$
	t-BuONa	Cs_2CO_3	*t*-BuONa	Cs_2CO_3	*t*-BuONa	Cs_2CO_3	*t*-BuONa	Cs_2CO_3
RuPhos	37	31	34	50	88	59	65	34
XantPhos	100	47	100	61	87	39	72	89
BrettPhos	71	68	41	55	73	35	84	31
IPent-HCl	100	28	96	100	100	100	100	67
dppf	55	100	77	66	93	59	91	60
BINAP	66	68	97	97	100	78	100	85
DavePhos	39	31	53	61	71	42	65	50
*t*BuXPhos	42	36	39	44	73	36	57	46
*t*BuBrettPhos	32	46	43	62	72	54	81	39
P(*o*-PhMe)$_3$	52	22	46	34	71	38	41	26

Table S2. Chromatographic yields (%) of the assigned target products for the pyridine–ring bromoimidazo[1,5-a]pyridines under the screened ligand/base/nucleophile combinations.

**5-bromoimidazo[1,5-a]pyridine (13)**								
	$PhCONH_2$	$PhCONH_2$	$PhNH_2$	$PhNH_2$	Morph	Morph	$BnNH_2$	$BnNH_2$
	t-BuONa	Cs_2CO_3	*t*-BuONa	Cs_2CO_3	*t*-BuONa	Cs_2CO_3	*t*-BuONa	Cs_2CO_3
RuPhos	0	0	0	0	7	10	0	2
XantPhos	42	14	0	29	65	45	2	24
BrettPhos	2	7	0	0	5	2	0	3
IPent-HCl	1	1	0	2	82	14	0	20
dppf	0	3	0	1	12	11	3	26
BINAP	1	4	0	1	31	68	3	44
DavePhos	0	1	0	0	9	4	0	2
*t*BuXPhos	0	1	0	0	2	0	0	3
*t*BuBrettPhos	0	2	0	0	3	0	0	1
P(*o*-PhMe)$_3$	0	2	0	0	2	0	0	0
**6-bromoimidazo[1,5-a]pyridine (14)**								
	$PhCONH_2$	$PhCONH_2$	$PhNH_2$	$PhNH_2$	Morph	Morph	$BnNH_2$	$BnNH_2$
	t-BuONa	Cs_2CO_3	*t*-BuONa	Cs_2CO_3	*t*-BuONa	Cs_2CO_3	*t*-BuONa	Cs_2CO_3
RuPhos	0,1	0	0	0	14	0	0	0
XantPhos	0,1	0	0	0	74	10	0	0
BrettPhos	0,1	0,1	11	3	4	11	0	0
IPent-HCl	0	0	75	0	100	16	50	3
dppf	0	0,6	0	0	5	0	0	0
BINAP	0,1	0,3	0	0	19	0	100	0
DavePhos	0,1	0	0	0	11	0	0	0
*t*BuXPhos	0,1	5,1	0	2	0	0	0	3
*t*BuBrettPhos	0	27,3	0	3	0	0	0	1
P(*o*-PhMe)$_3$	0,1	0,1	0	0	0	0	0	0

**7-bromoimidazo[1,5-a]pyridine (15)**								
	$PhCONH_2$	$PhCONH_2$	$PhNH_2$	$PhNH_2$	Morph	Morph	$BnNH_2$	$BnNH_2$
	t-BuONa	Cs_2CO_3	*t*-BuONa	Cs_2CO_3	*t*-BuONa	Cs_2CO_3	*t*-BuONa	Cs_2CO_3
RuPhos	0	0	0	24	17	13	0	4
XantPhos	4	14	16	17	100	2	11	31
BrettPhos	0	3	0	11	16	0	7	4
IPent-HCl	1	0	0	3	79	3	4	4
dppf	0	29	0	4	23	1	4	0
BINAP	2	24	0	3	26	0	15	0
DavePhos	0	0	0	12	7	0	0	1
*t*BuXPhos	1	0	0	6	0	0	0	5
*t*BuBrettPhos	0	6	0	1	0	0	0	3
P(*o*-PhMe)$_3$	0	0	0	0	3	0	0	0
**8-bromoimidazo[1,5-a]pyridine (6)**								
	$PhCONH_2$	$PhCONH_2$	$PhNH_2$	$PhNH_2$	Morph	Morph	$BnNH_2$	$BnNH_2$
	t-BuONa	Cs_2CO_3	*t*-BuONa	Cs_2CO_3	*t*-BuONa	Cs_2CO_3	*t*-BuONa	Cs_2CO_3
RuPhos	0	0	0	4	1	4	0	3
XantPhos	80	8	43	11	6	0	40	39
BrettPhos	30	45	0	5	3	1	0	7
IPent-HCl	20	0	15	59	41	78	52	59
dppf	0	39	5	9	3	2	24	0
BINAP	3	38	13	48	11	46	56	78
DavePhos	0	0	0	13	2	2	30	16
*t*BuXPhos	0	6	0	2	0	0	0	4
*t*BuBrettPhos	0	8	0	7	0	0	0	2
P(*o*-PhMe)$_3$	0	0	0	0	0	0	0	0

NMR spectra

Compound 2

1H NMR (500 MHz, Chloroform-*d*)

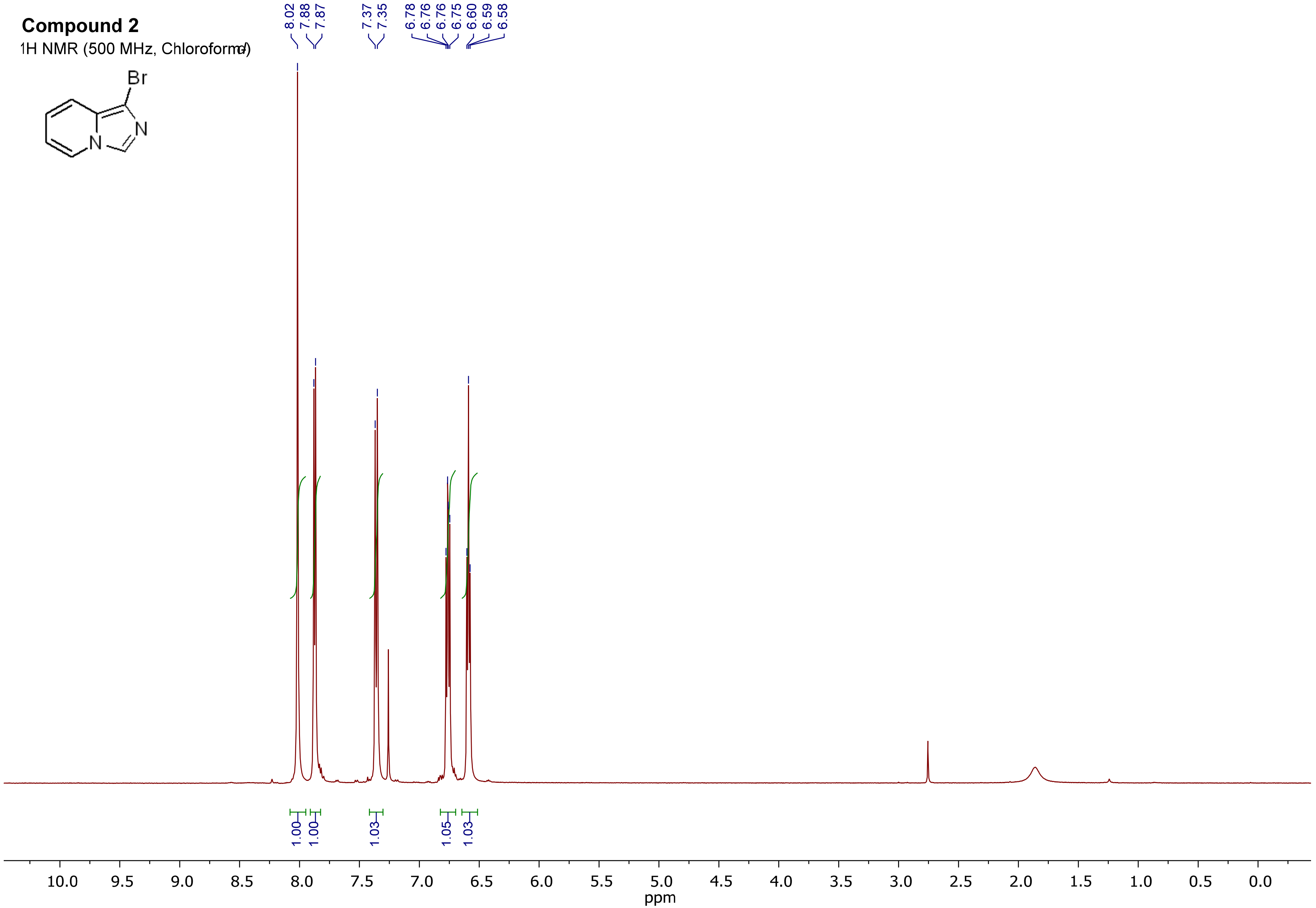

Compound 2

13C NMR (101 MHz, Chloroform-d)

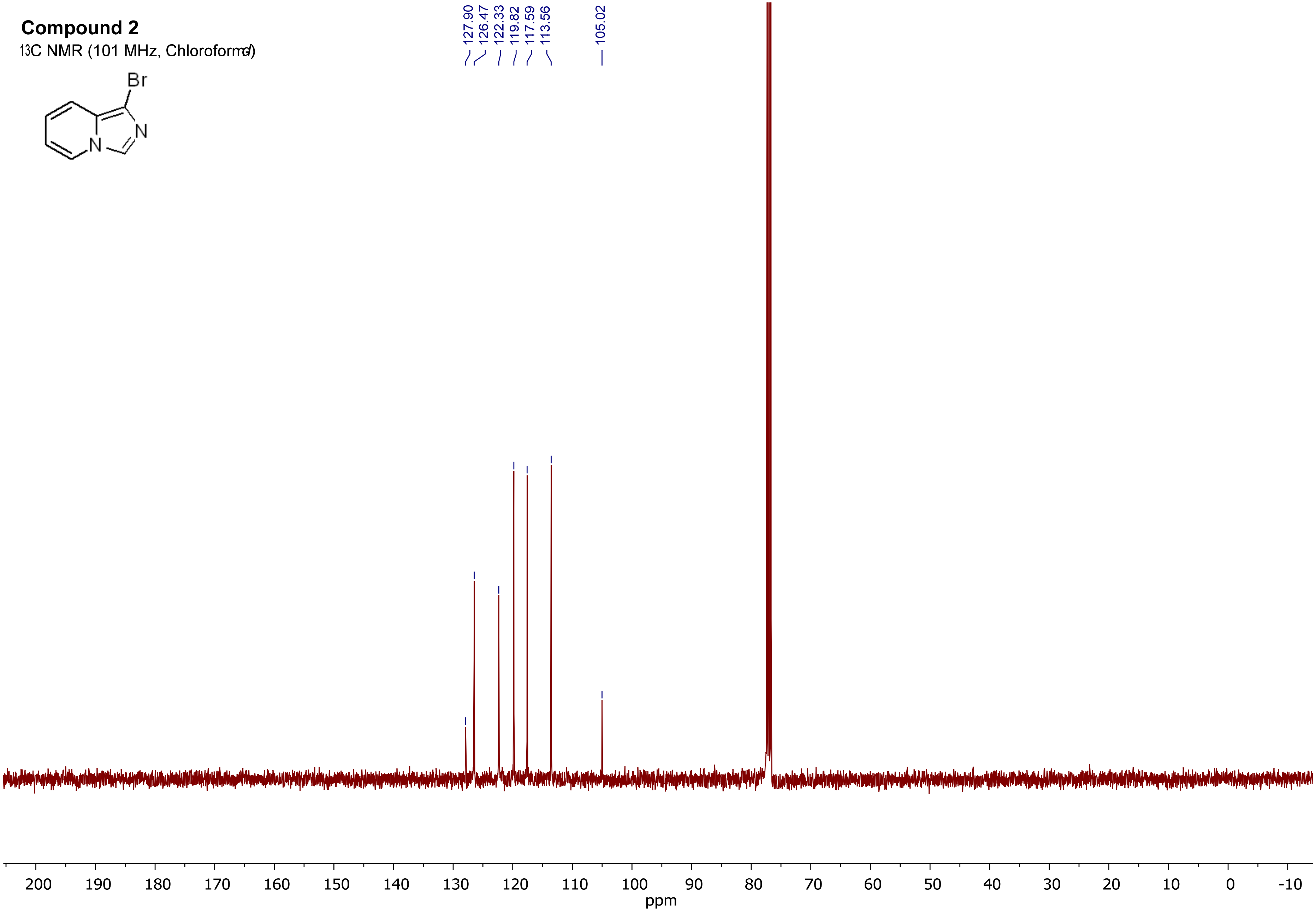

Compound 3

1H NMR (500 MHz, Chloroform-*d*)

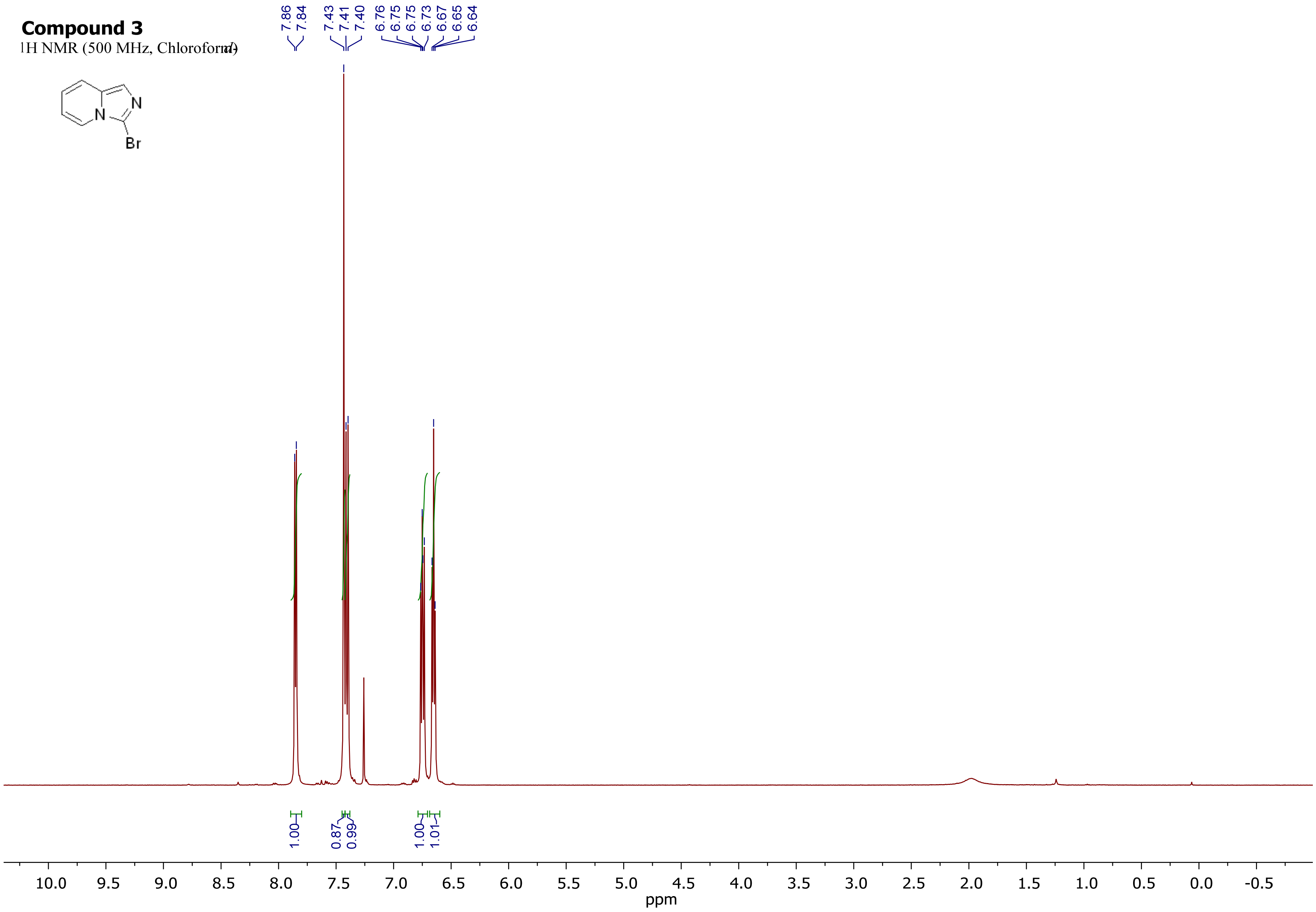

Compound 3

13C NMR (101 MHz, Chloroform-*d*)

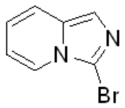

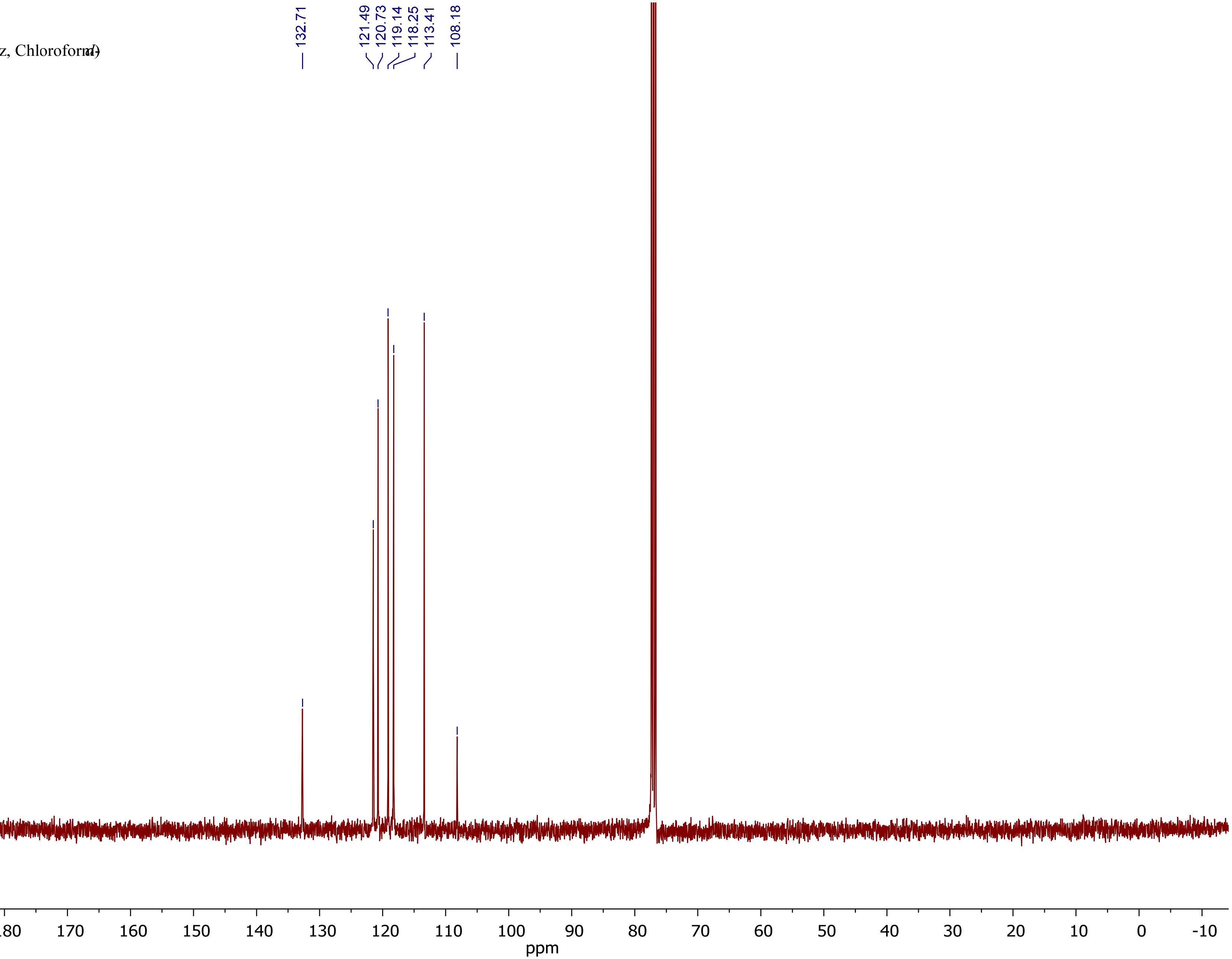

Compound 6

1H NMR (500 MHz, Chloroform-*d*)

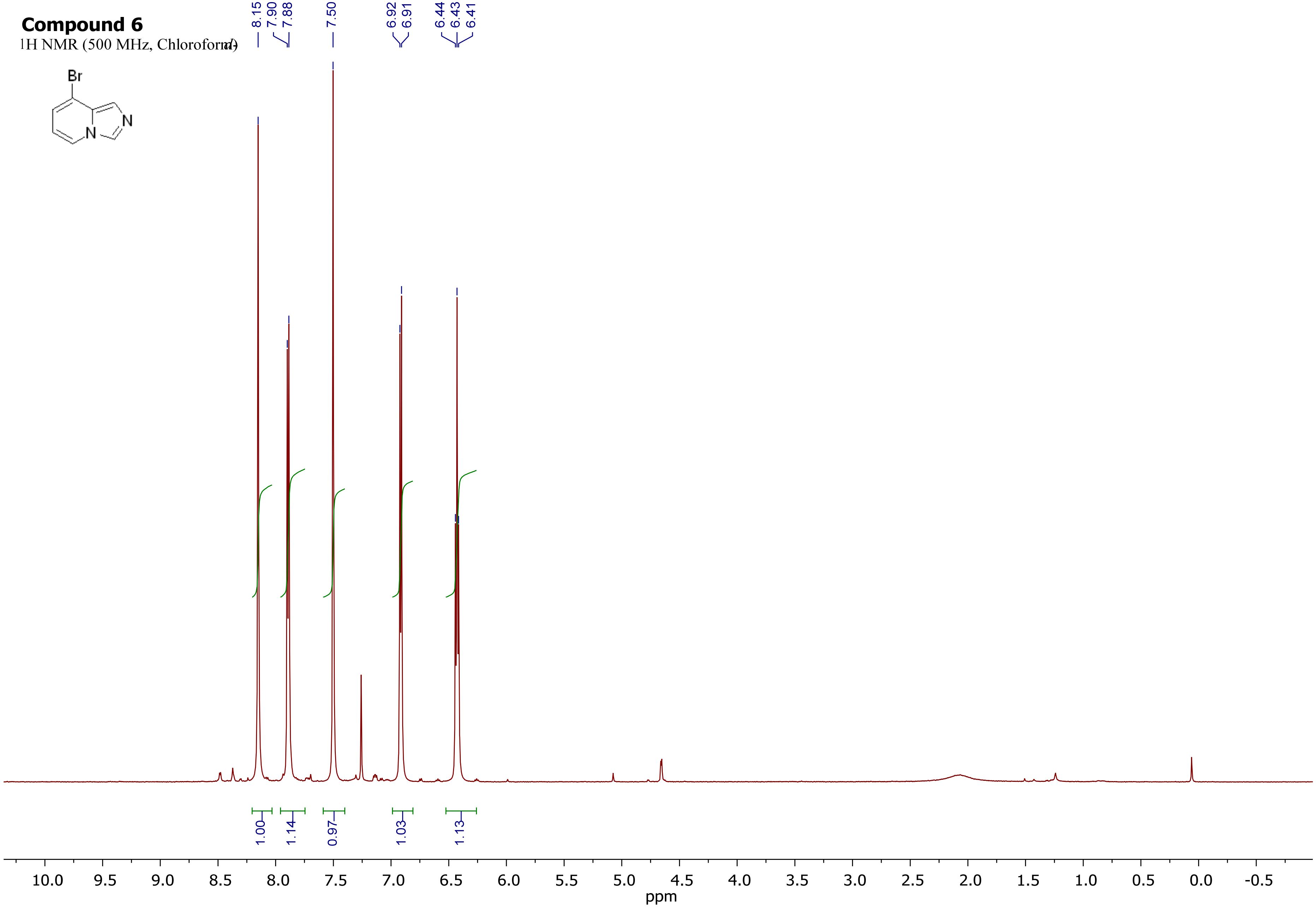

Compound 6

13C NMR (126 MHz, Chloroform-*d*)

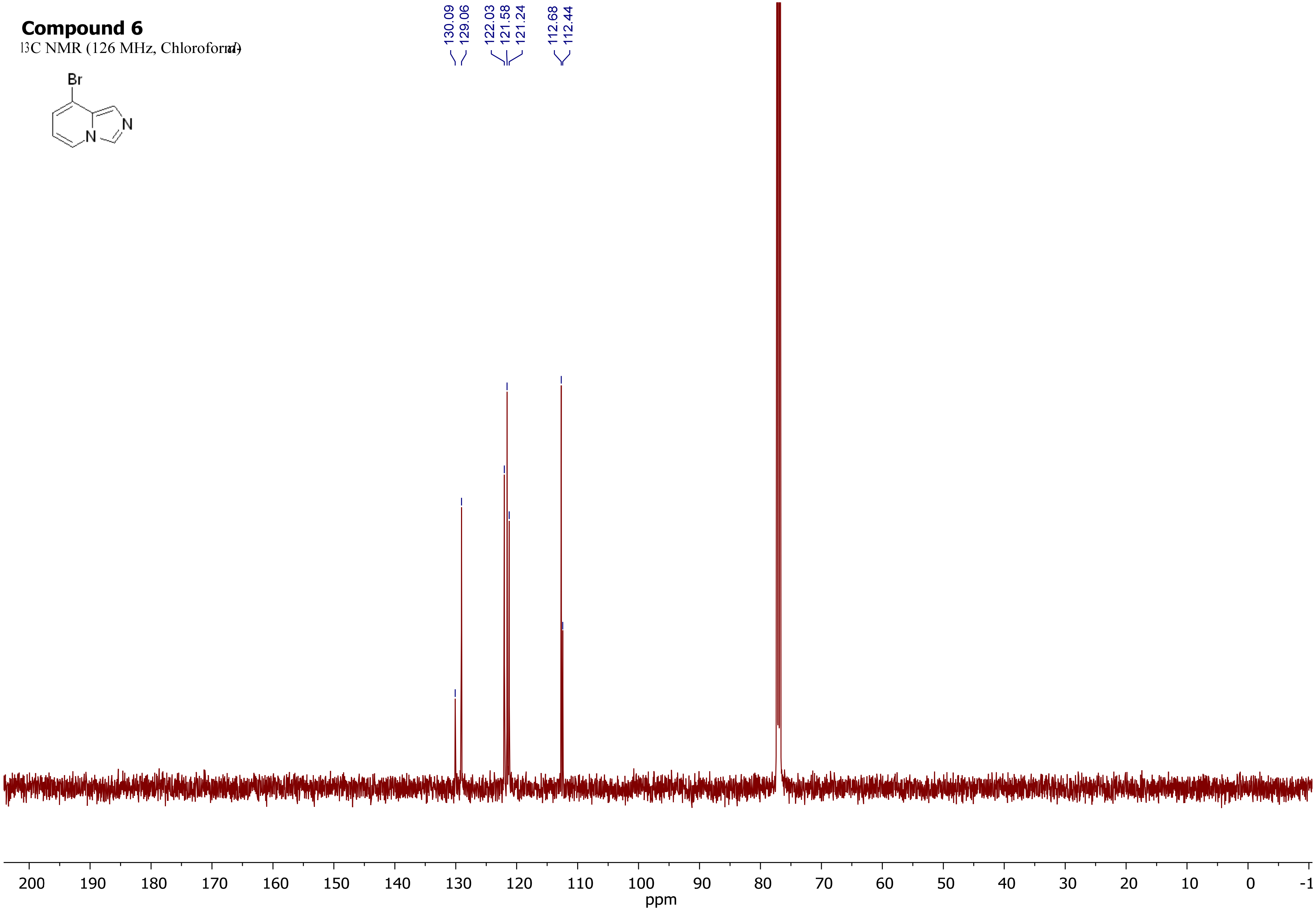

Compound 13

1H NMR (500 MHz, Chloroform-d)

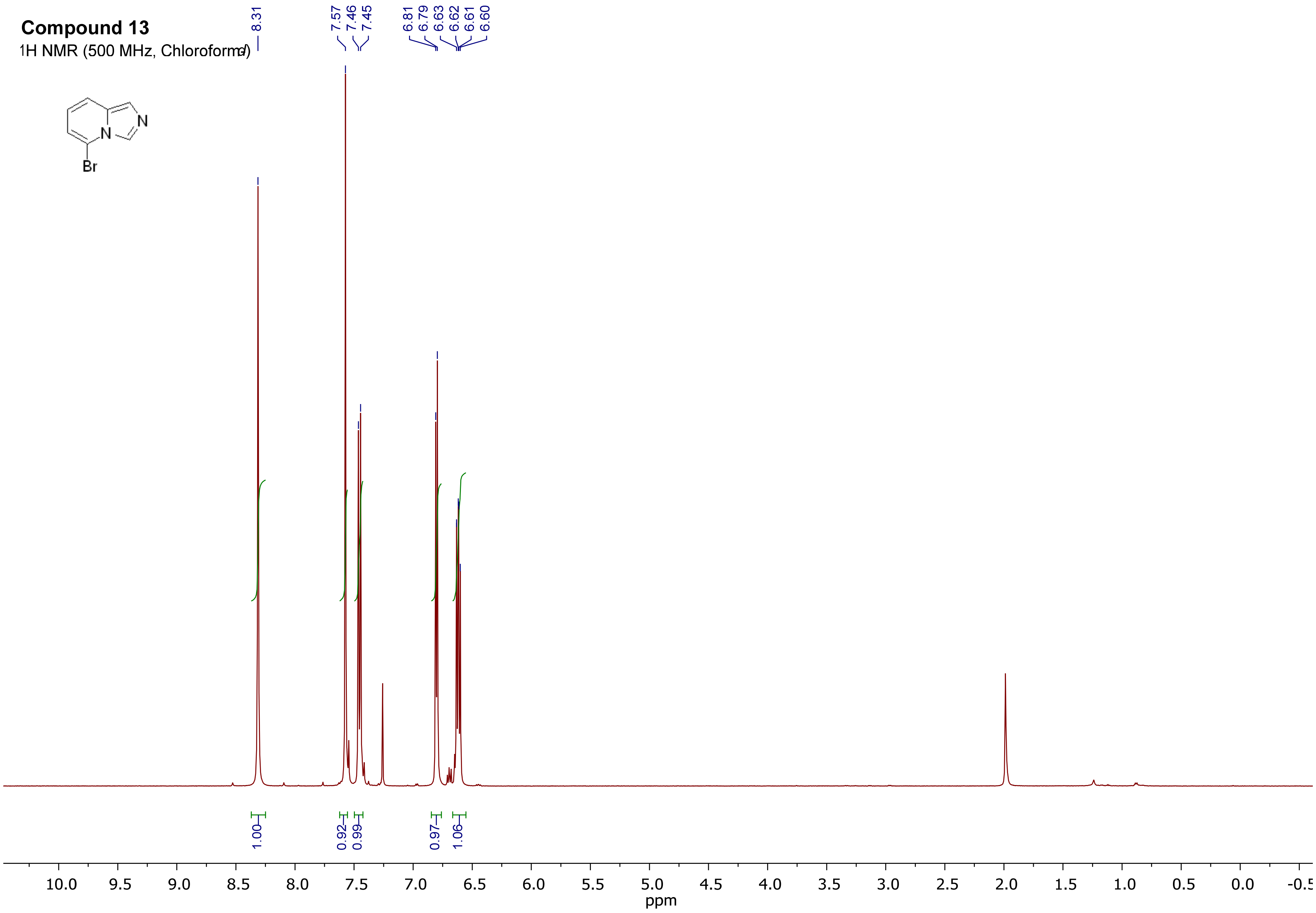

Compound 13

13C NMR (126 MHz, Chloroform-d)

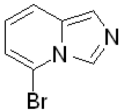

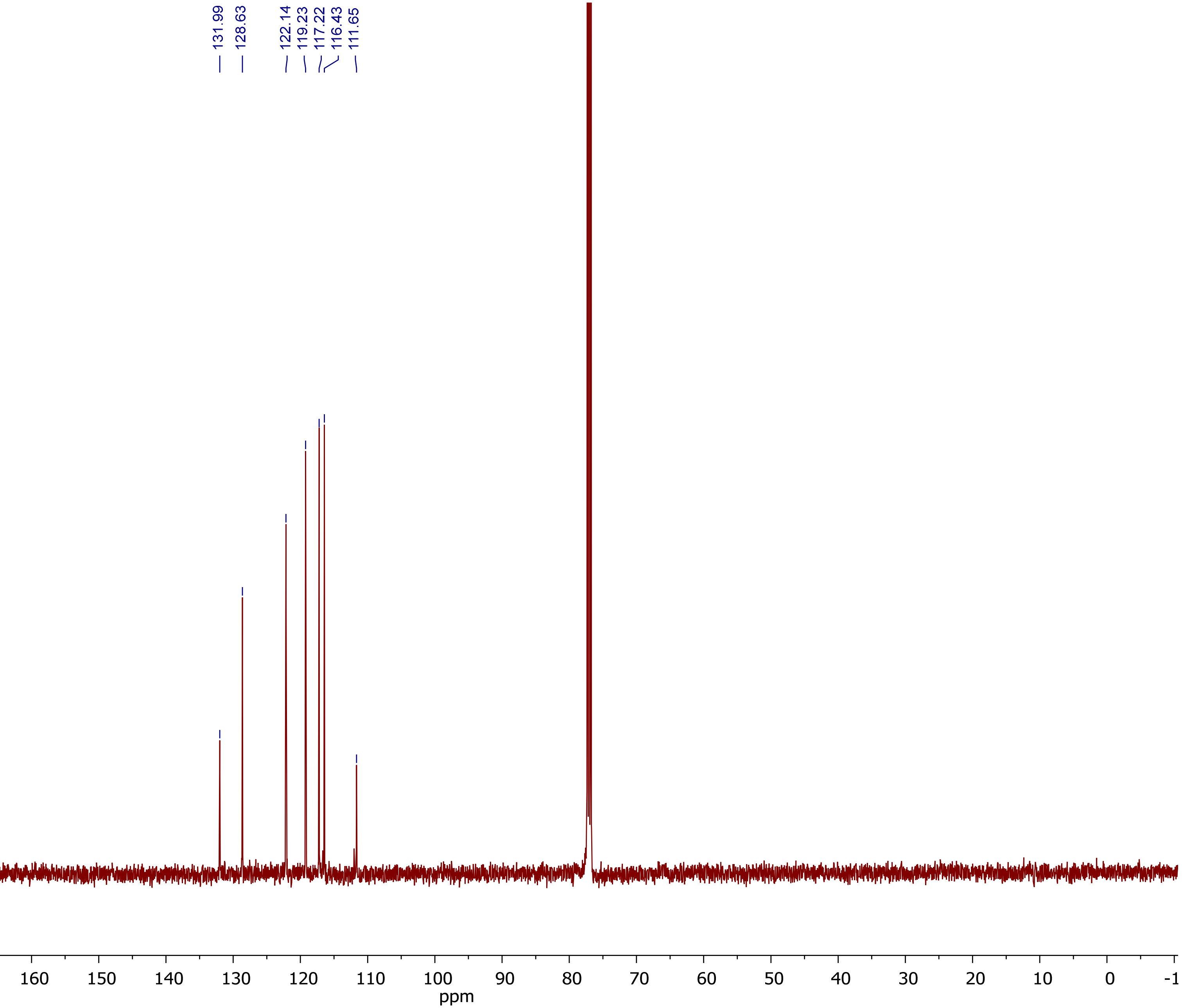

Compound 14
1H NMR (500 MHz, Chloroform-d)

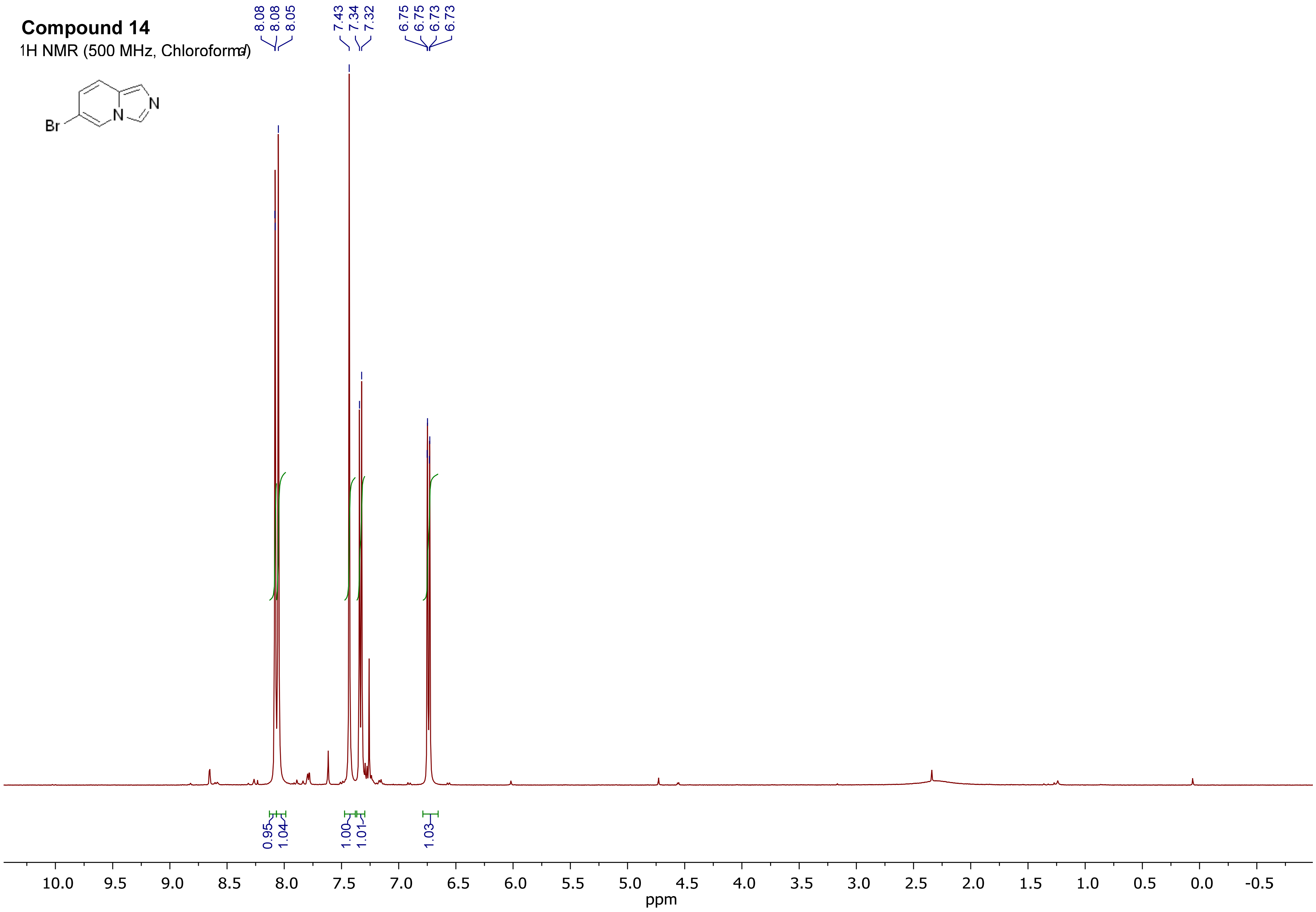

Compound 14

^{13}C NMR (101 MHz, Chloroform-*d*)

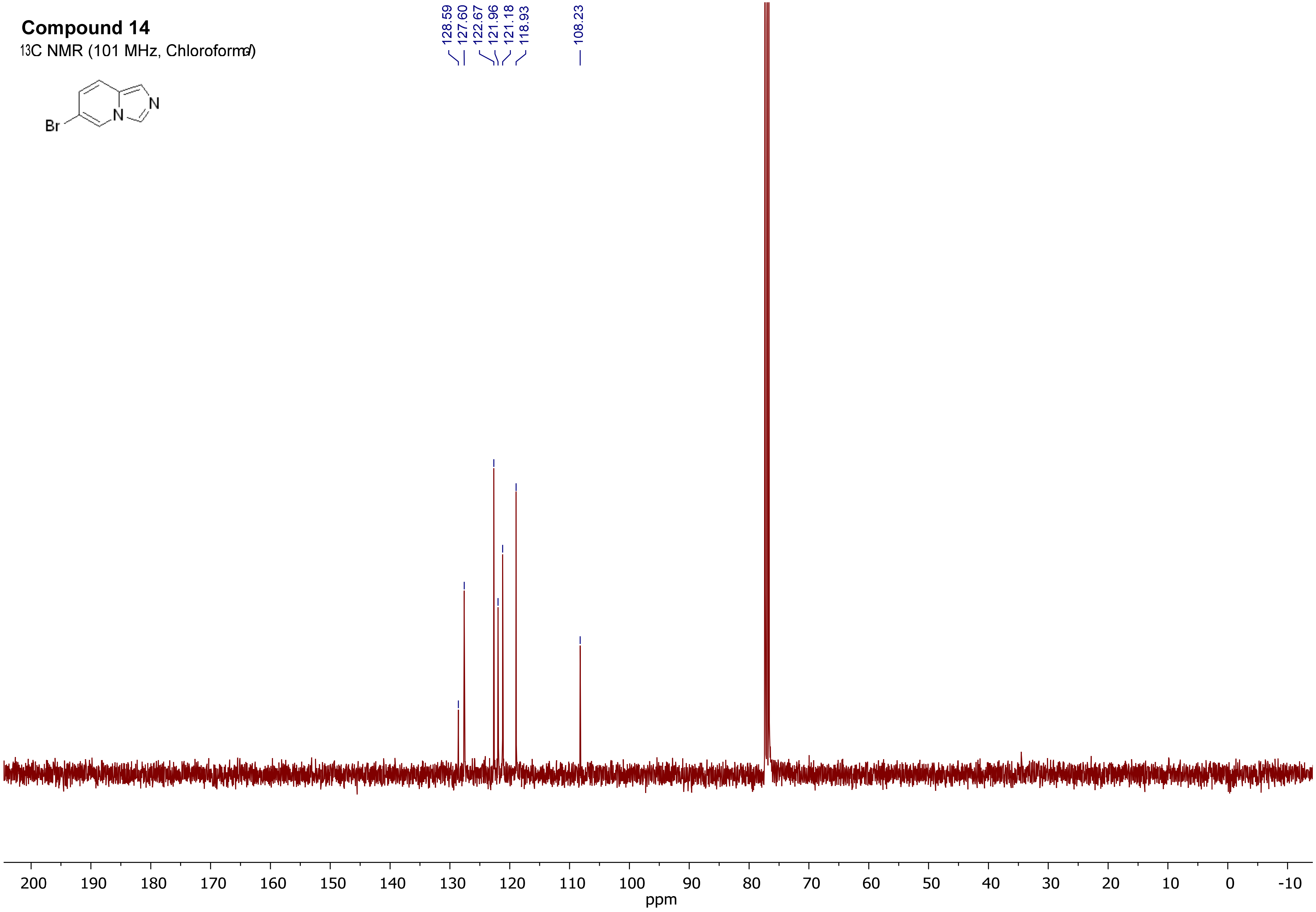

Compound 15

1H NMR (500 MHz, Chloroform-d)

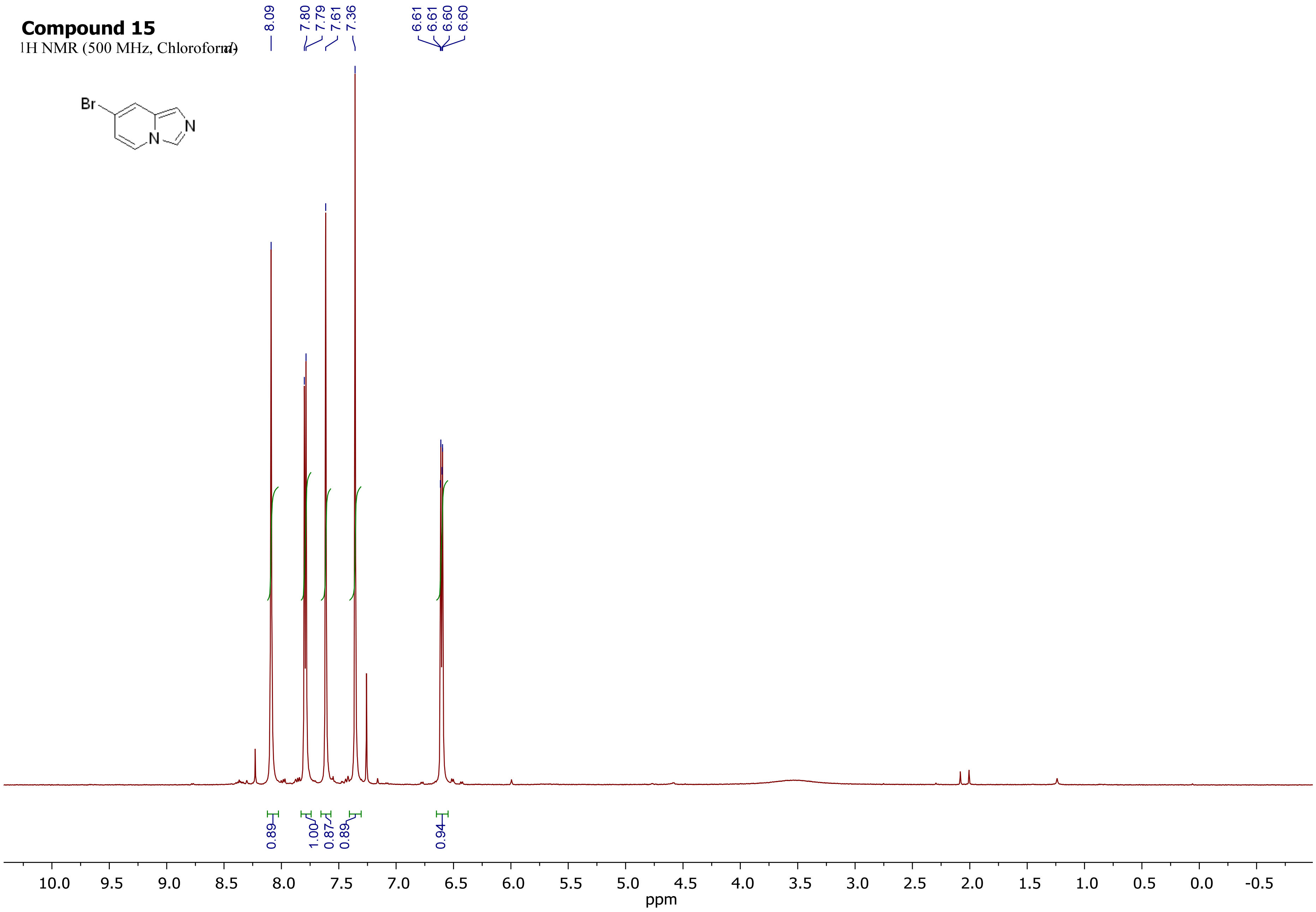

Compound 15

^{13}C NMR (101 MHz, Chloroform-*d*)

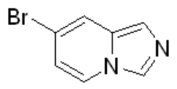

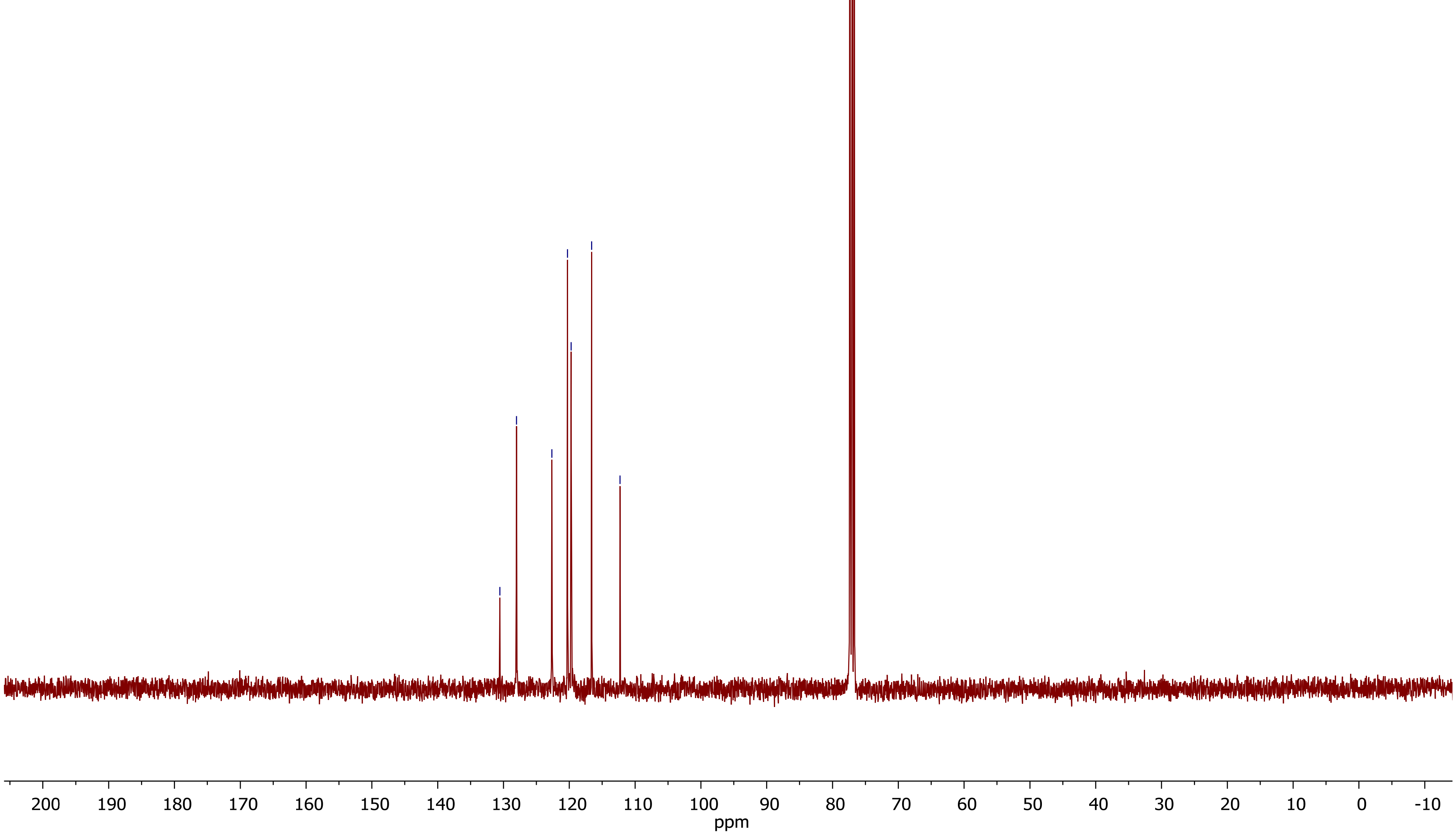

Compound 24

1H NMR (500 MHz, DMSO-d_6)

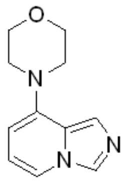

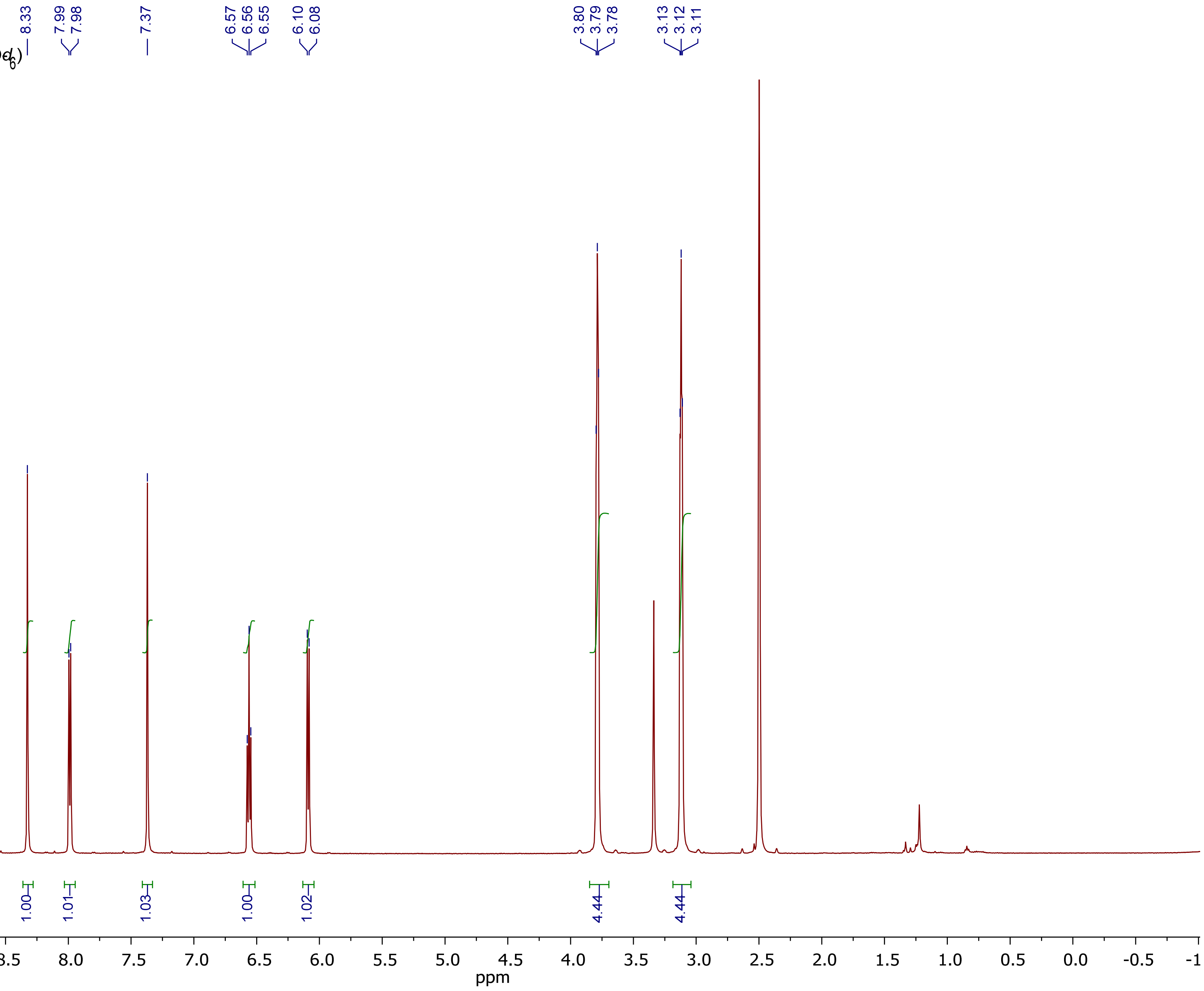

Compound 24

13C NMR (126 MHz, DMSO-d_6)

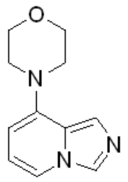

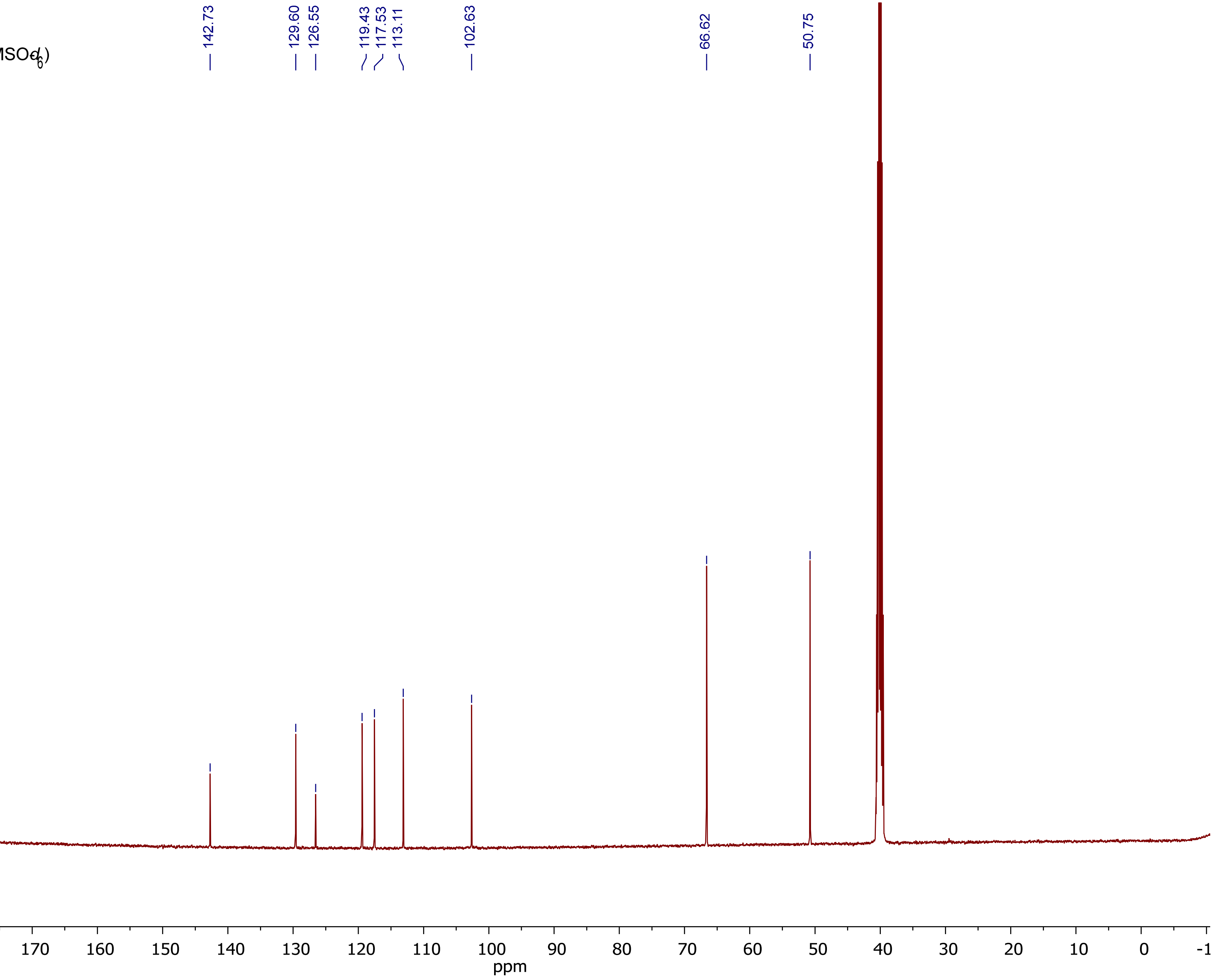

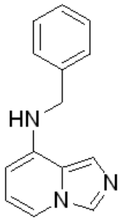

Compound 25

1H NMR (500 MHz, DMSO-d_6)

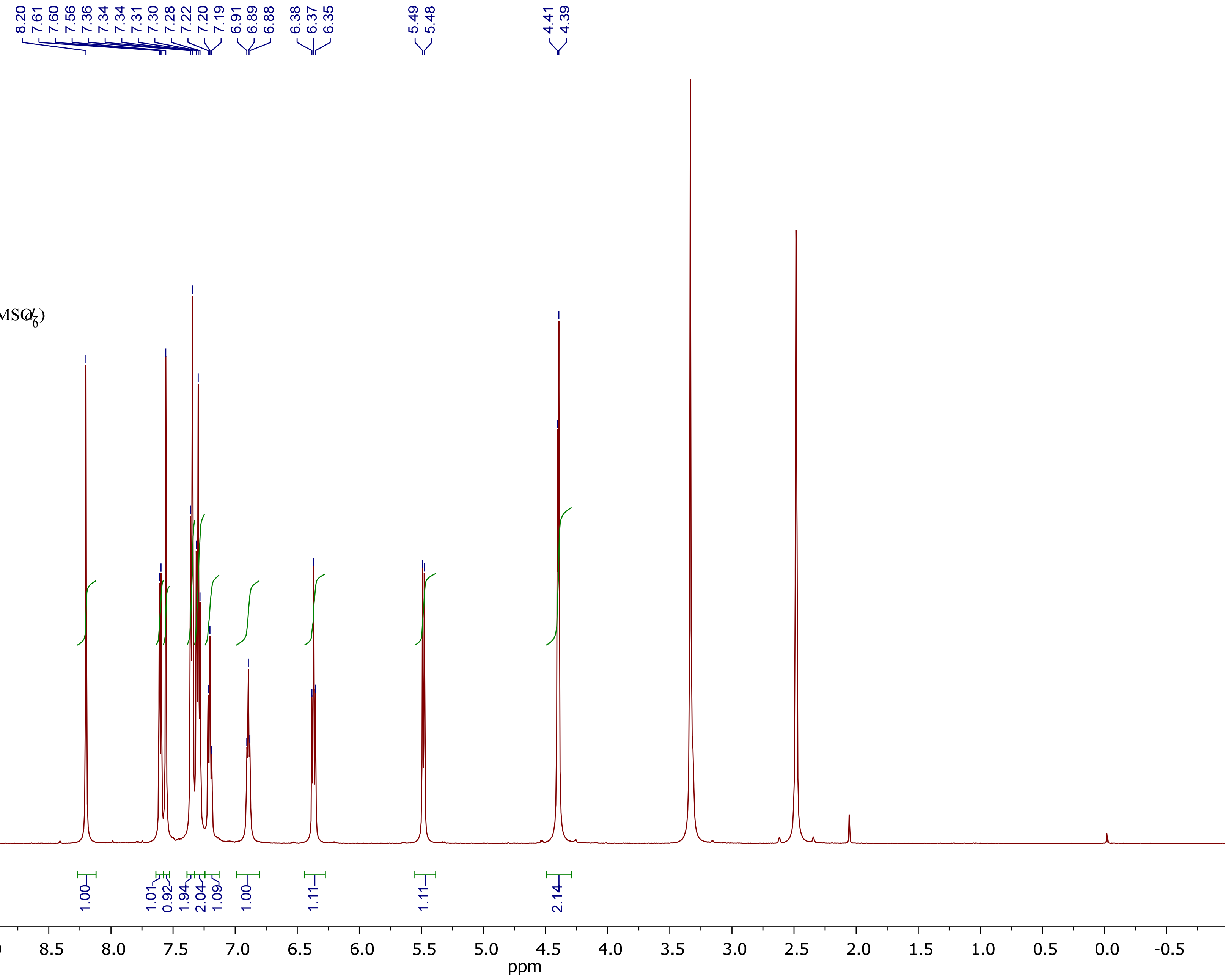

Compound 25
13C NMR (126 MHz, DMSO-d_6)

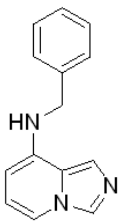

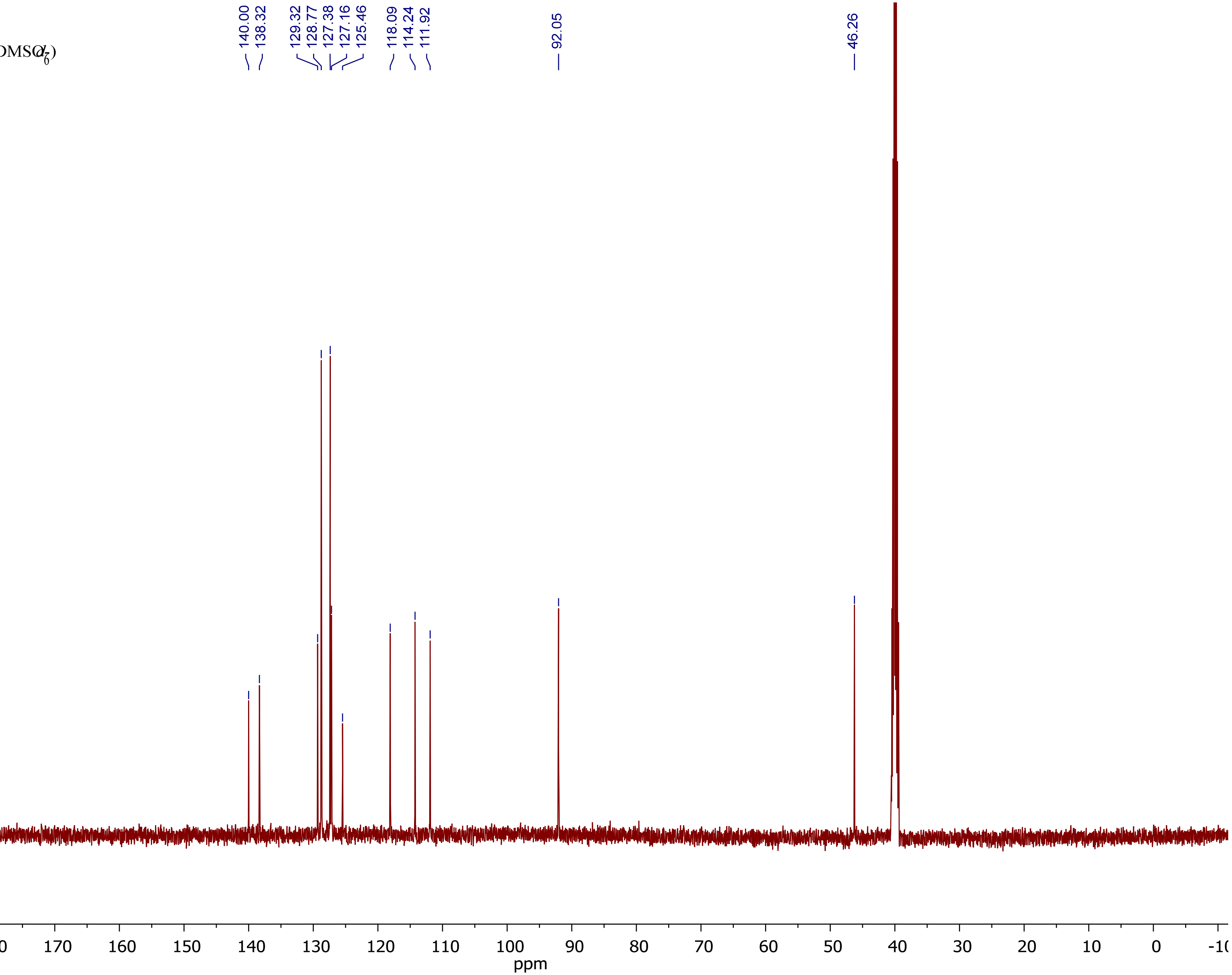

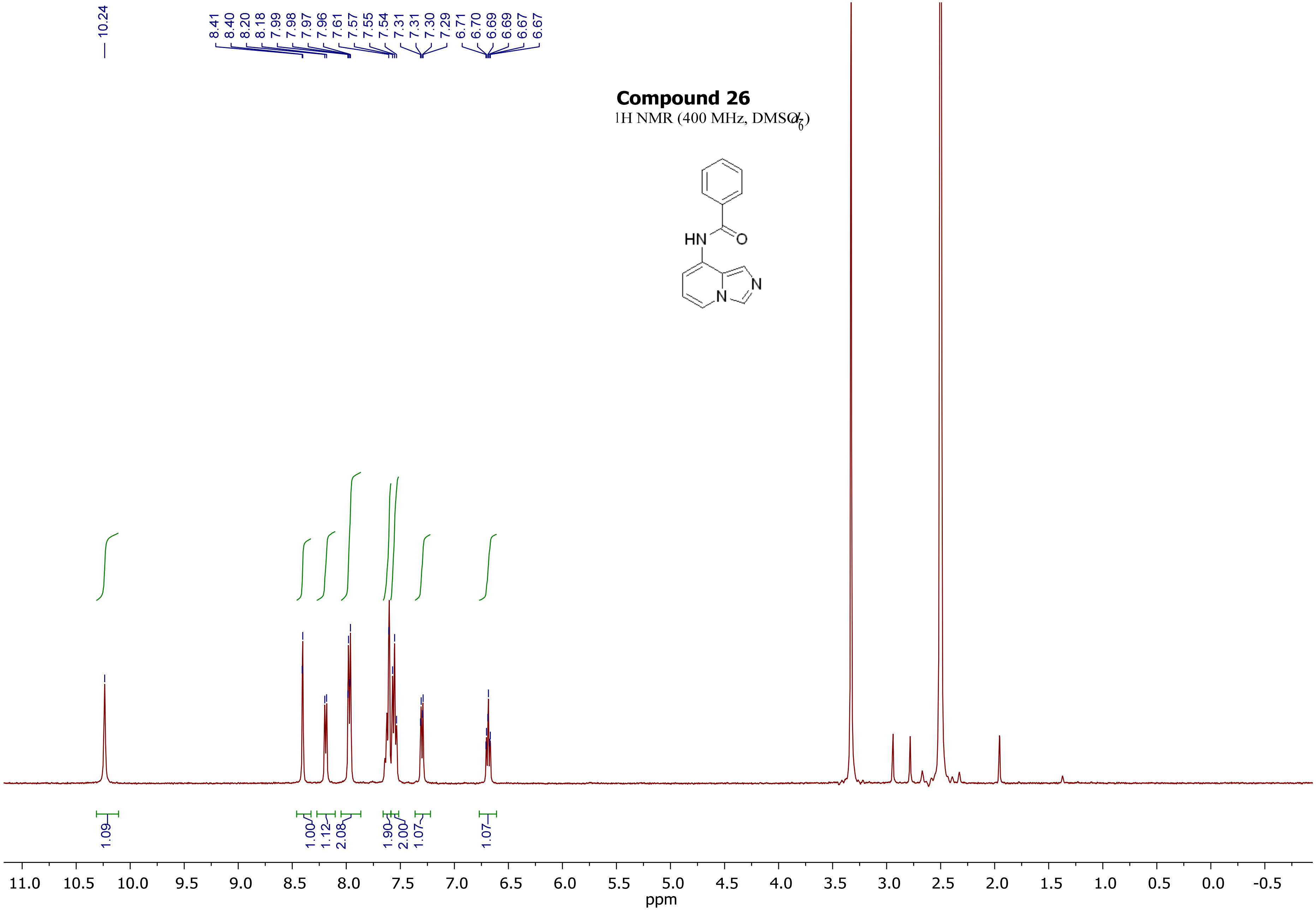
Compound 26
1H NMR (400 MHz, DMSO-d6)
HN
O
N
N
10.24
8.41 8.40 8.20 8.18 7.99 7.98 7.97 7.96 7.61 7.57 7.55 7.54 7.31 7.31 7.30 7.29 6.71 6.70 6.69 6.69 6.67 6.67
1.09 1.00 1.12 2.08 1.90 2.00 1.07 1.07
11.0 10.5 10.0 9.5 9.0 8.5 8.0 7.5 7.0 6.5 6.0 5.5 5.0 4.5 4.0 3.5 3.0 2.5 2.0 1.5 1.0 0.5 0.0 -0.5
ppm

Compound 26

13C NMR (101 MHz, Chloroform-*d*)

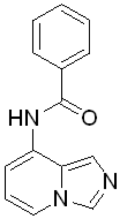

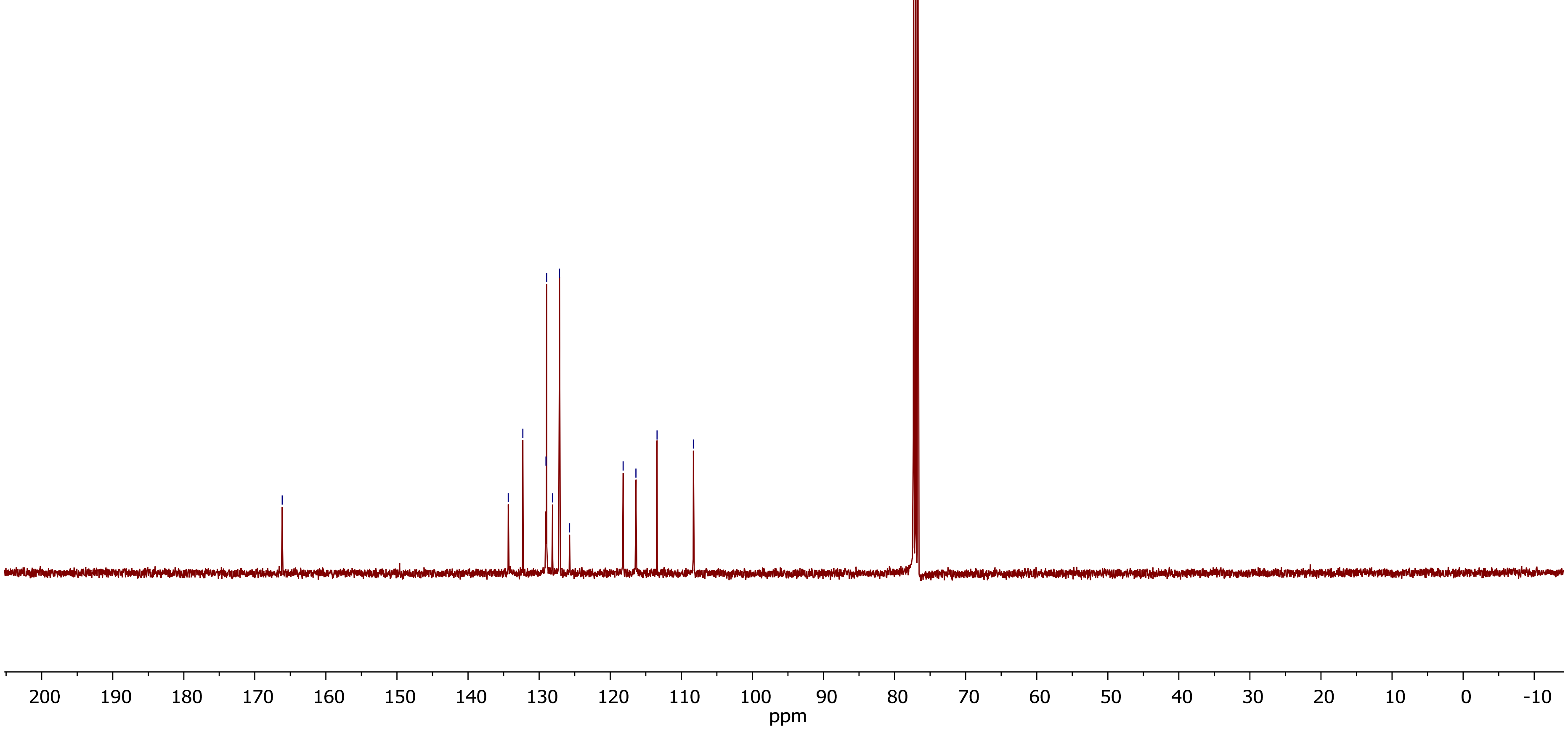

Compound 27

1H NMR (500 MHz, DMSO-d_6)

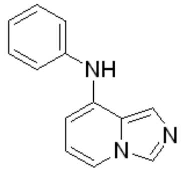

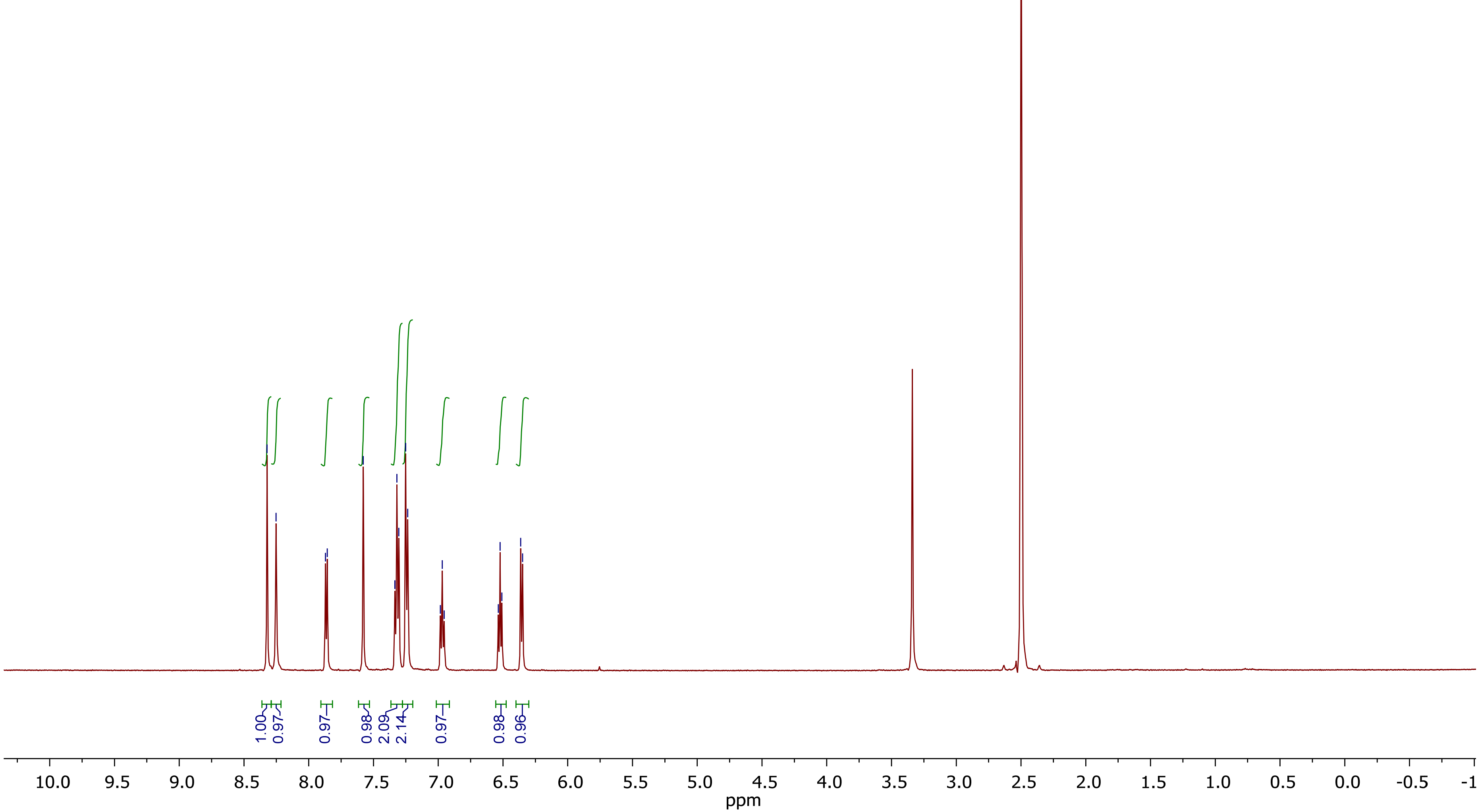

Compound 27

13C NMR (101 MHz, DMSO-d_6)

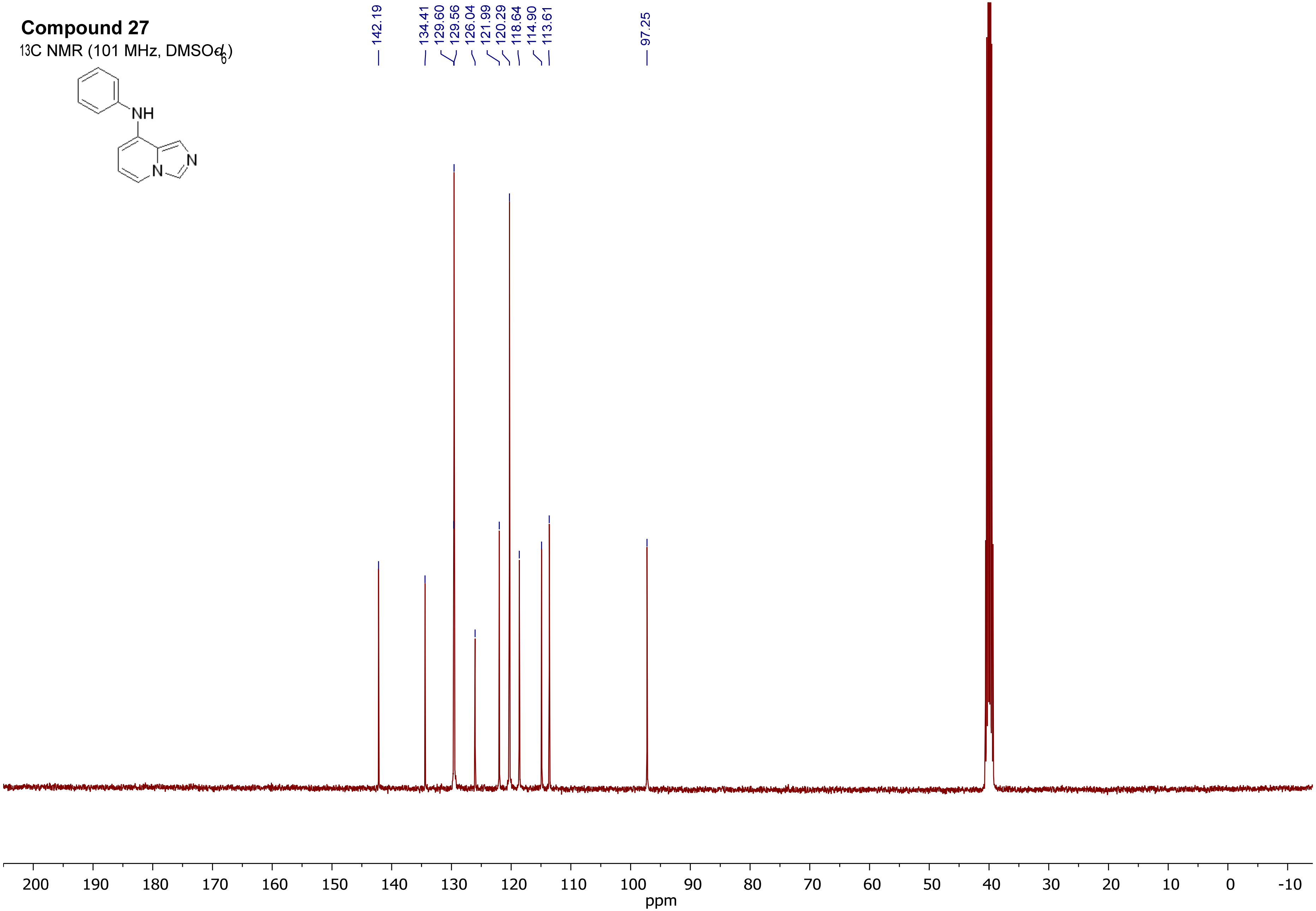

Compound 28

1H NMR (500 MHz, DMSO-d_6)

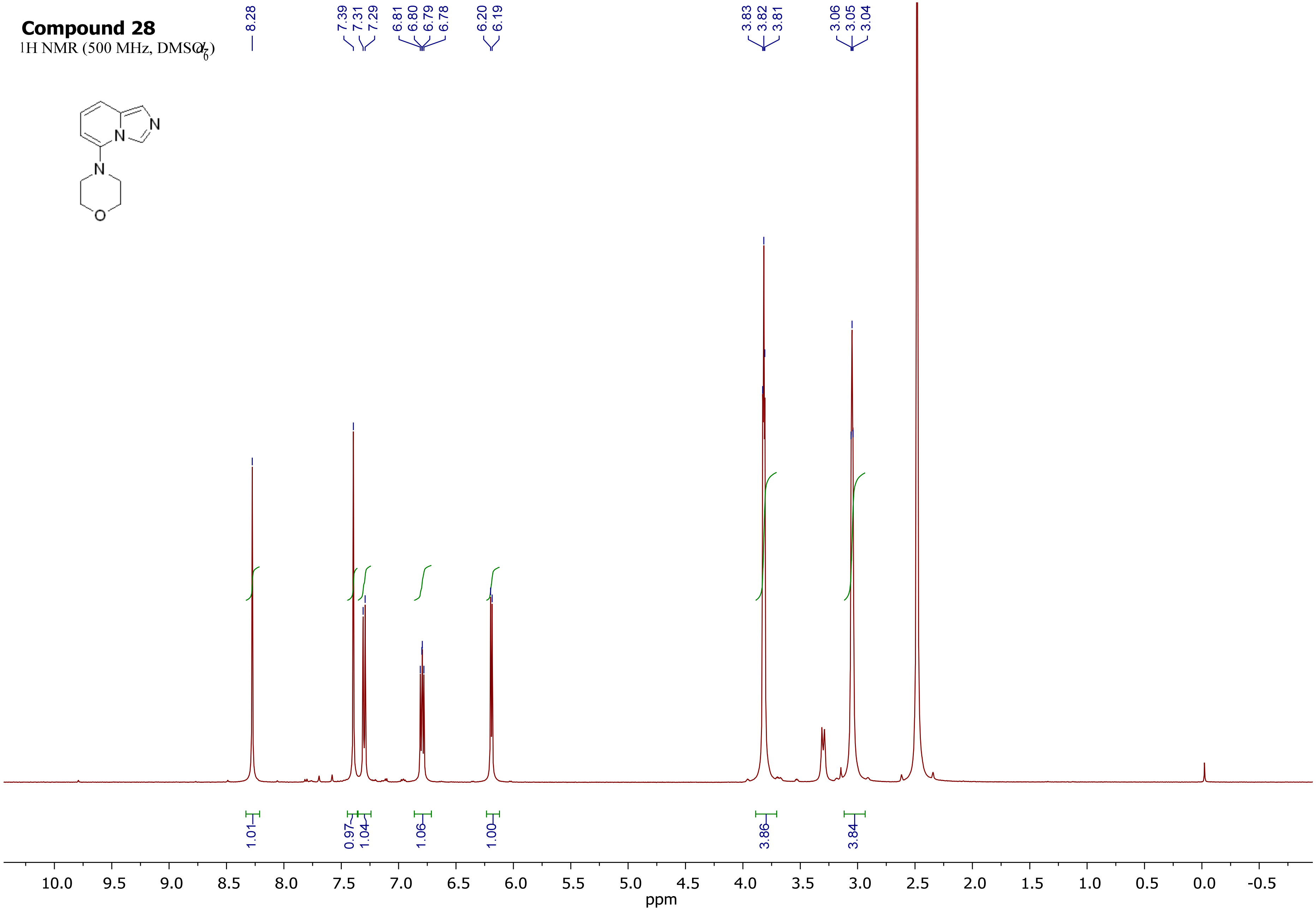

Compound 28

1H NMR (400 MHz, Chloroform-d)

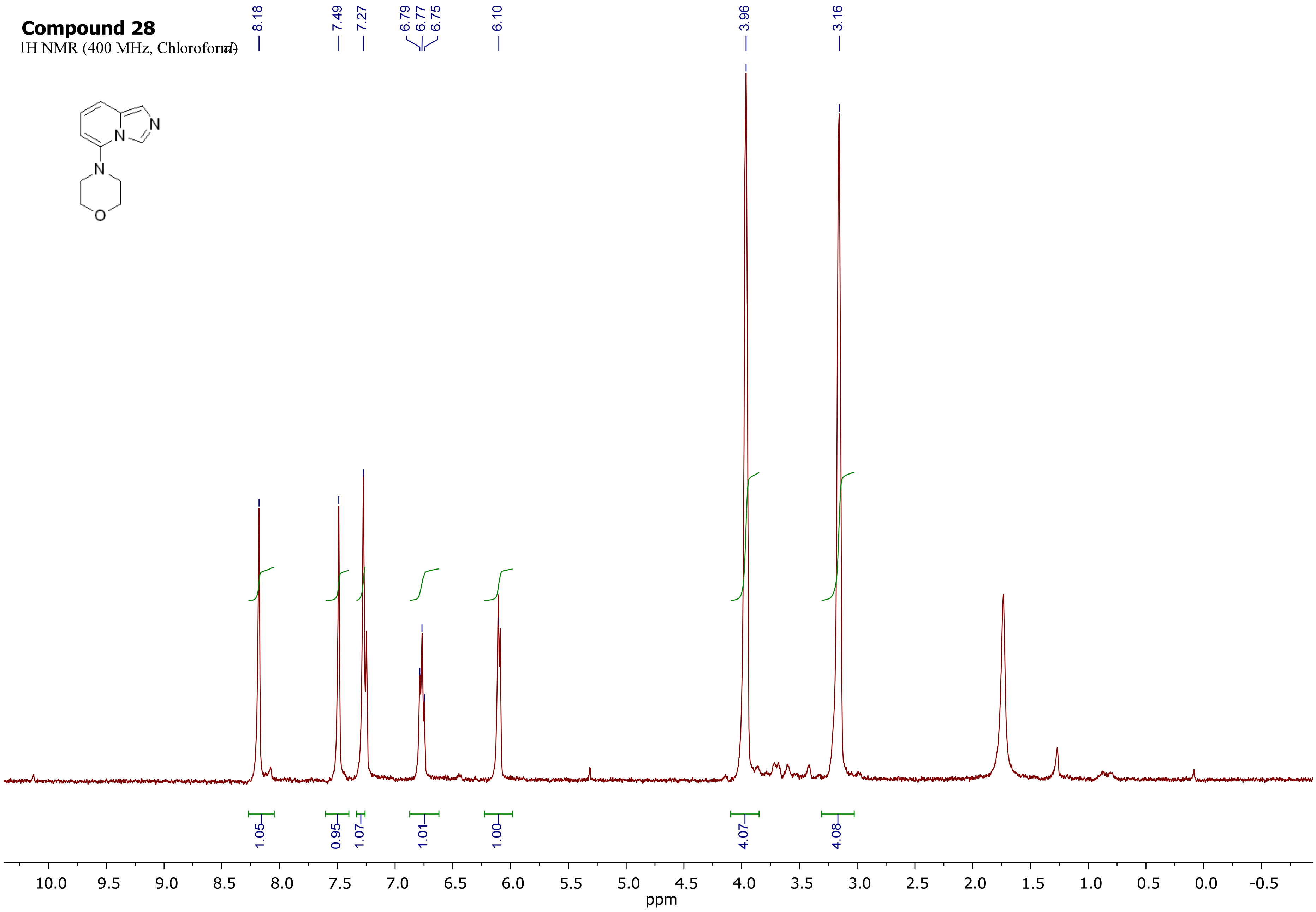

Compound 28

13C NMR (151 MHz, Chloroform-*d*)

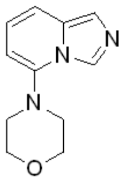

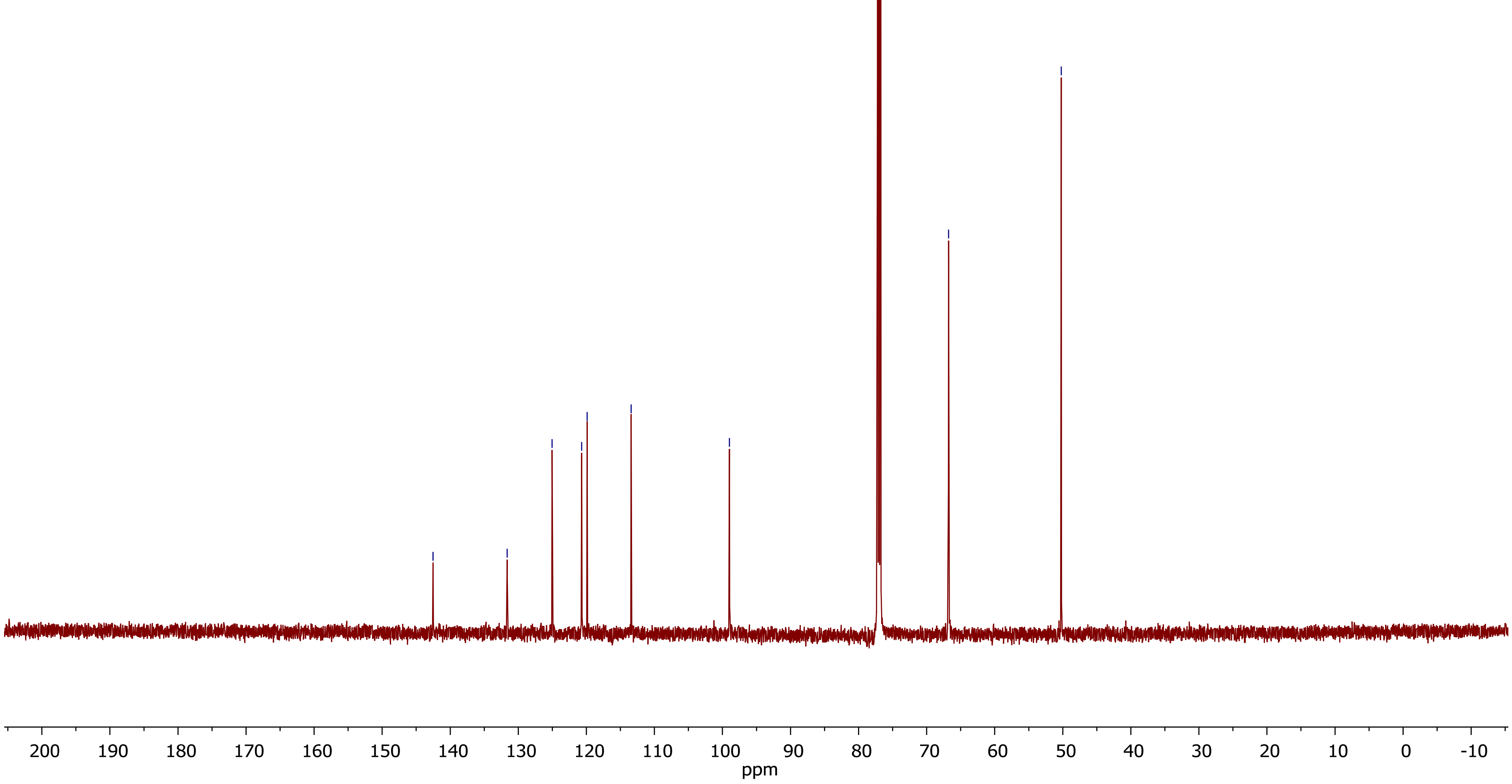

Compound 29

1H NMR (400 MHz, DMSO-d_6)

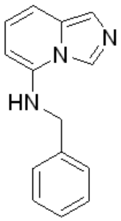

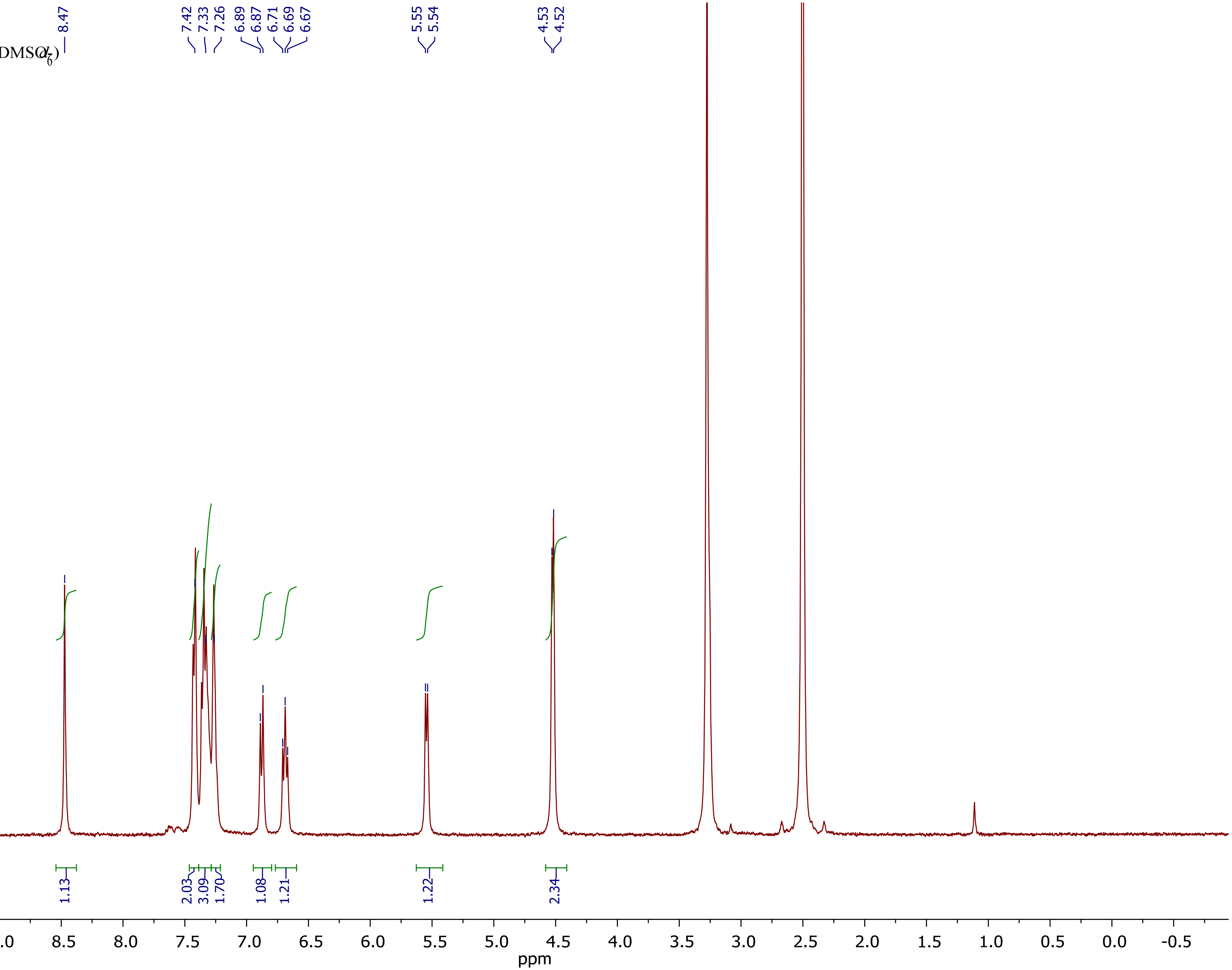

Compound 29

13C NMR (126 MHz, Chloroform-d)

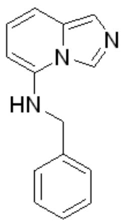

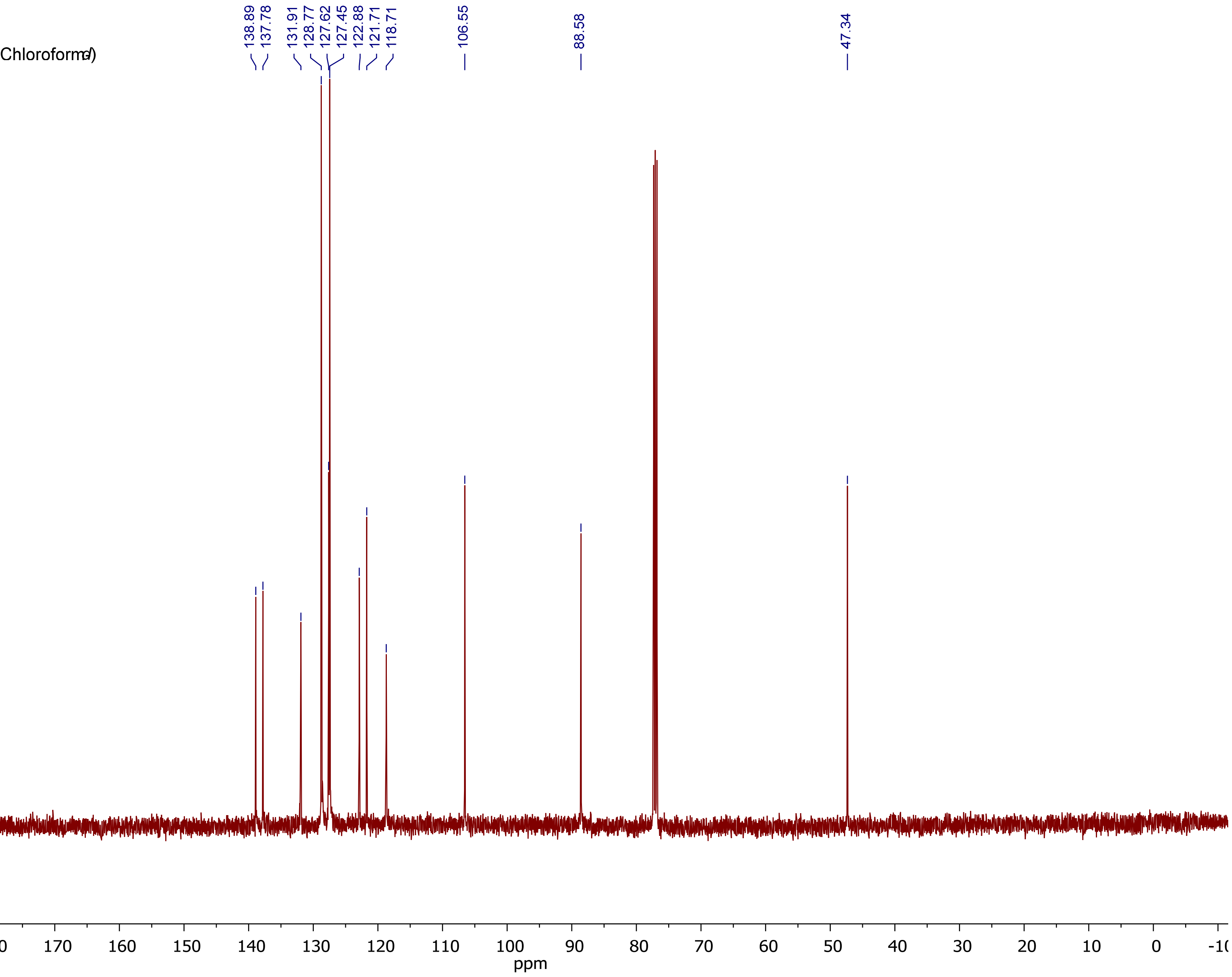

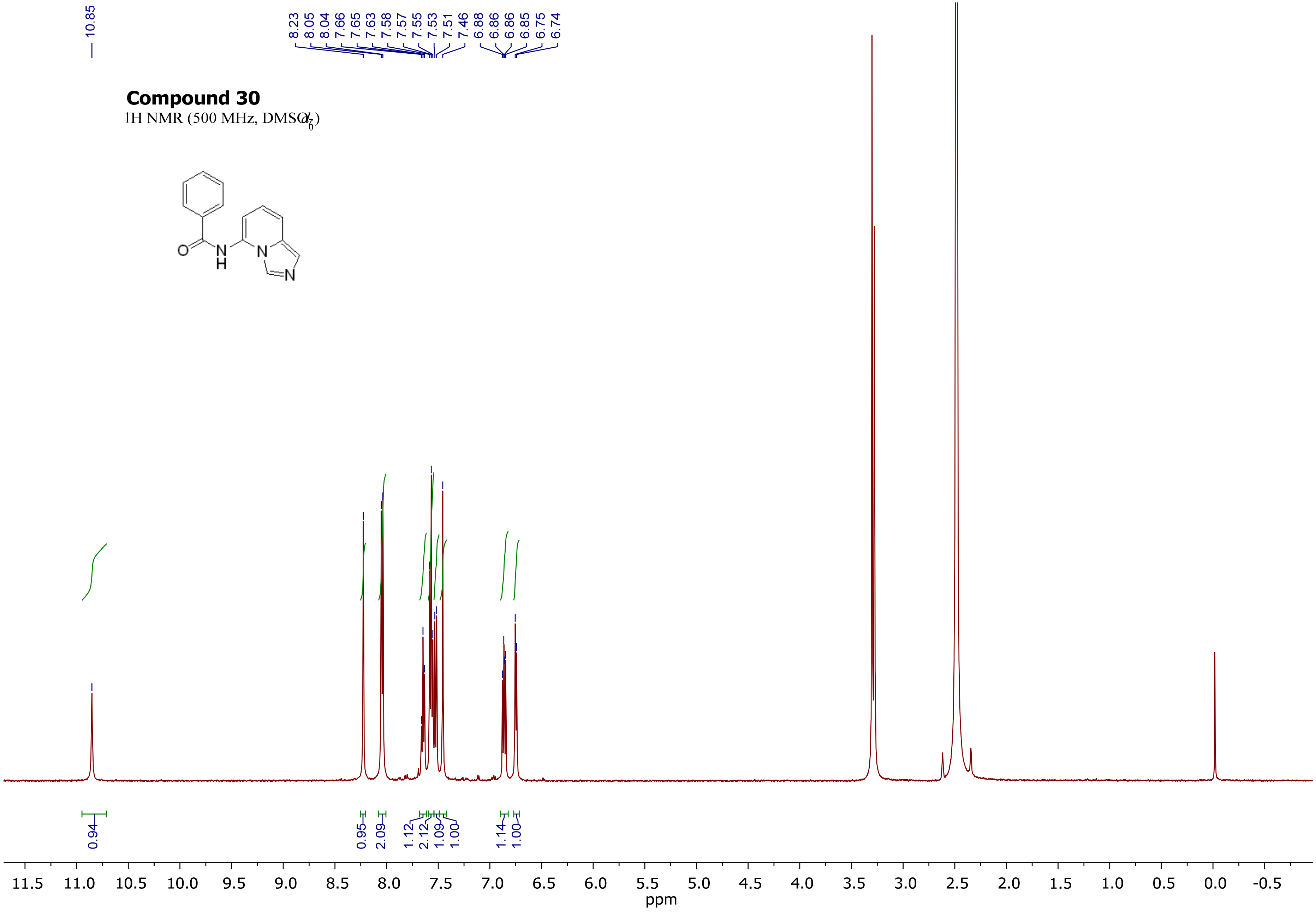
Compound 30
1H NMR (500 MHz, DMSO-d_6)
O
N
H
N
N
10.85
8.23
8.05
8.04
7.66
7.65
7.63
7.58
7.57
7.55
7.53
7.51
7.46
6.88
6.86
6.86
6.85
6.75
6.74
0.94
0.95
2.09
1.12
2.12
1.09
1.00
1.14
1.00
11.5 11.0 10.5 10.0 9.5 9.0 8.5 8.0 7.5 7.0 6.5 6.0 5.5 5.0 4.5 4.0 3.5 3.0 2.5 2.0 1.5 1.0 0.5 0.0 -0.5
ppm

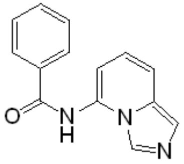

Compound 30

1H NMR (500 MHz, Chloroform-*d*)

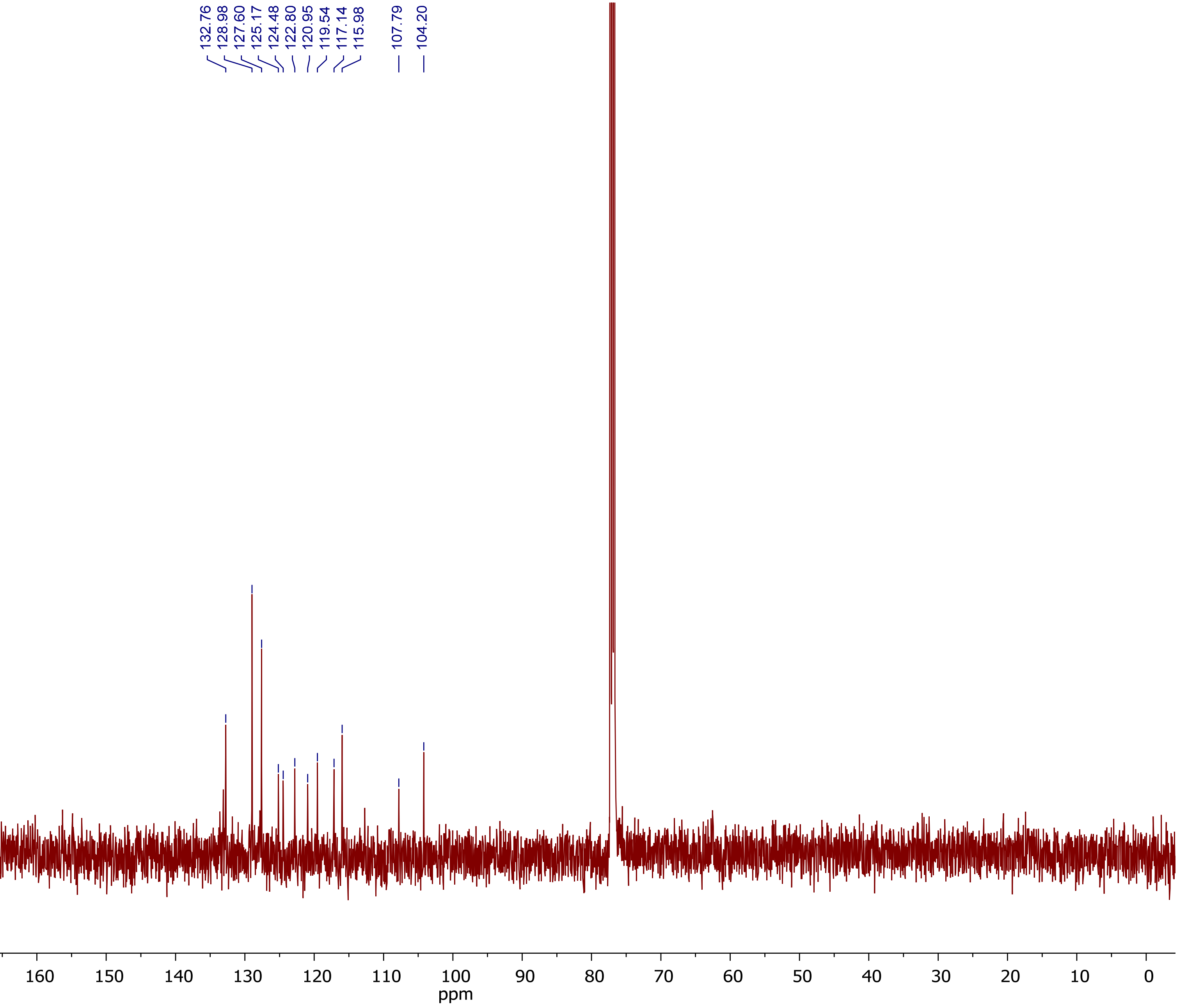

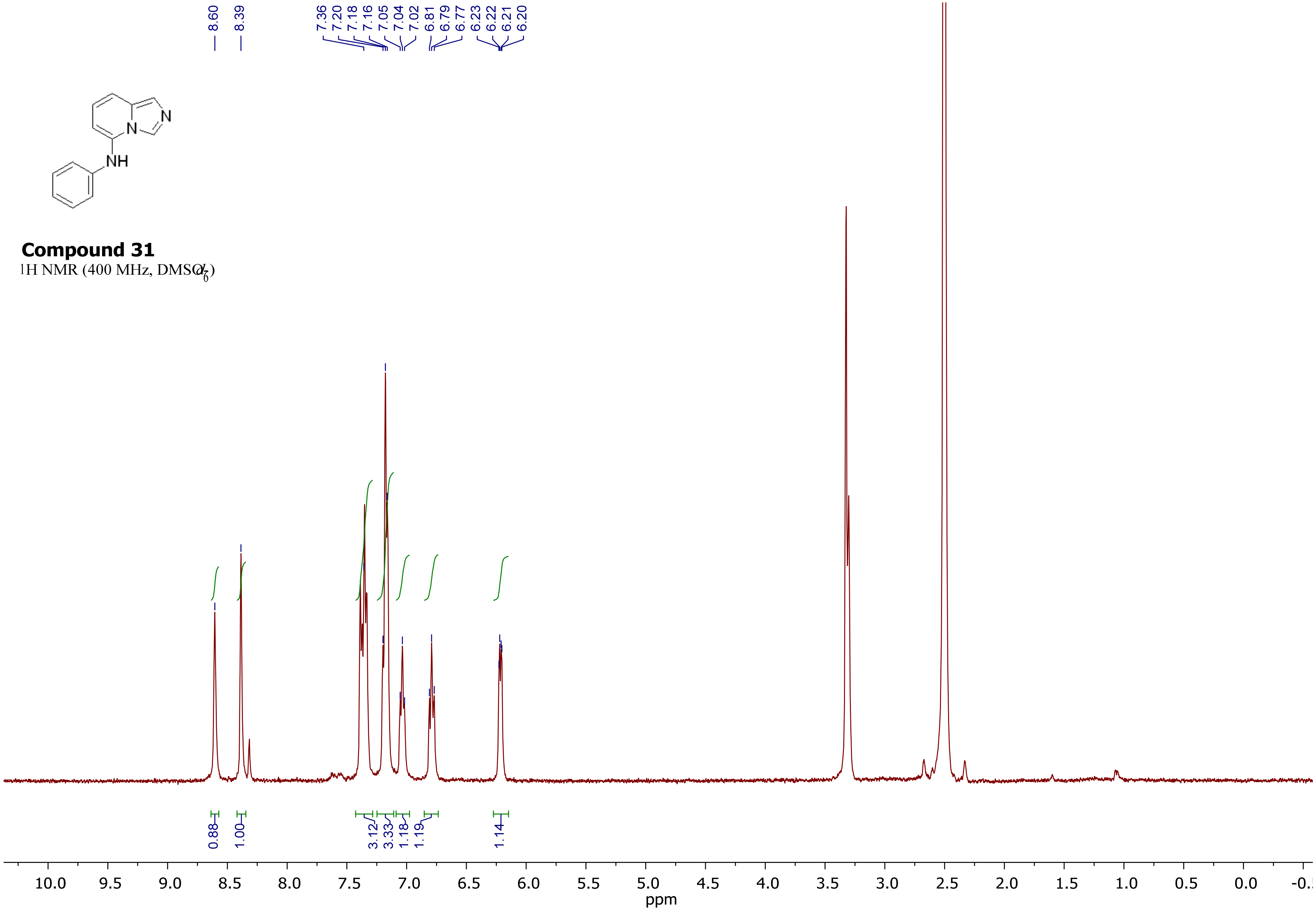

Compound 31

^{1}H NMR (400 MHz, DMSO-d_6)

Compound 31

13C NMR (101 MHz, Chloroform-*d*)

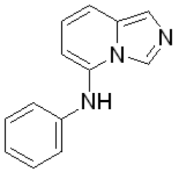

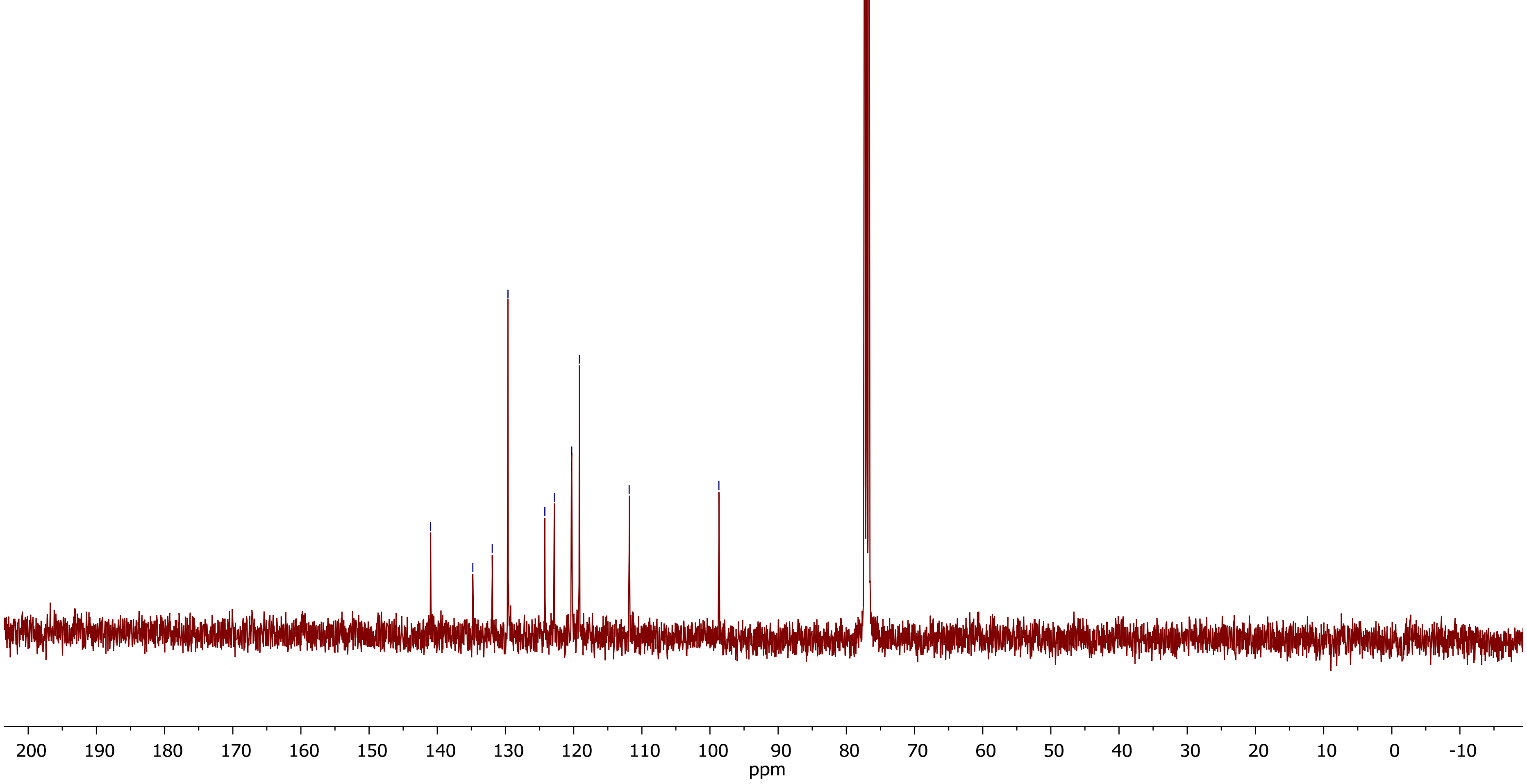

Compound 32

1H NMR (500 MHz, DMSO-d_6)

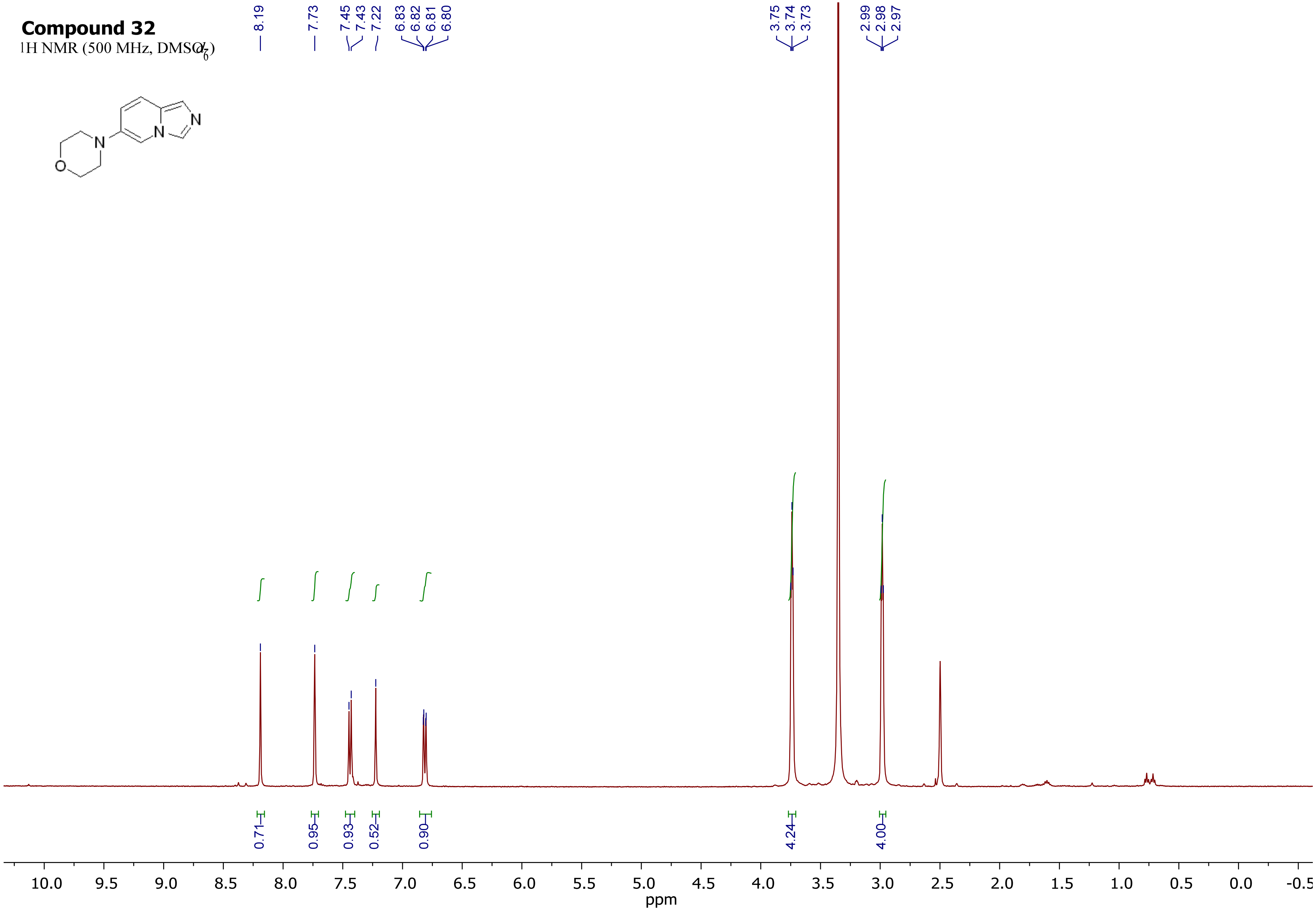

Compound 32

13C NMR (126 MHz, Chloroform-d)

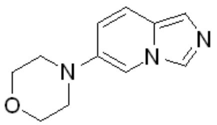

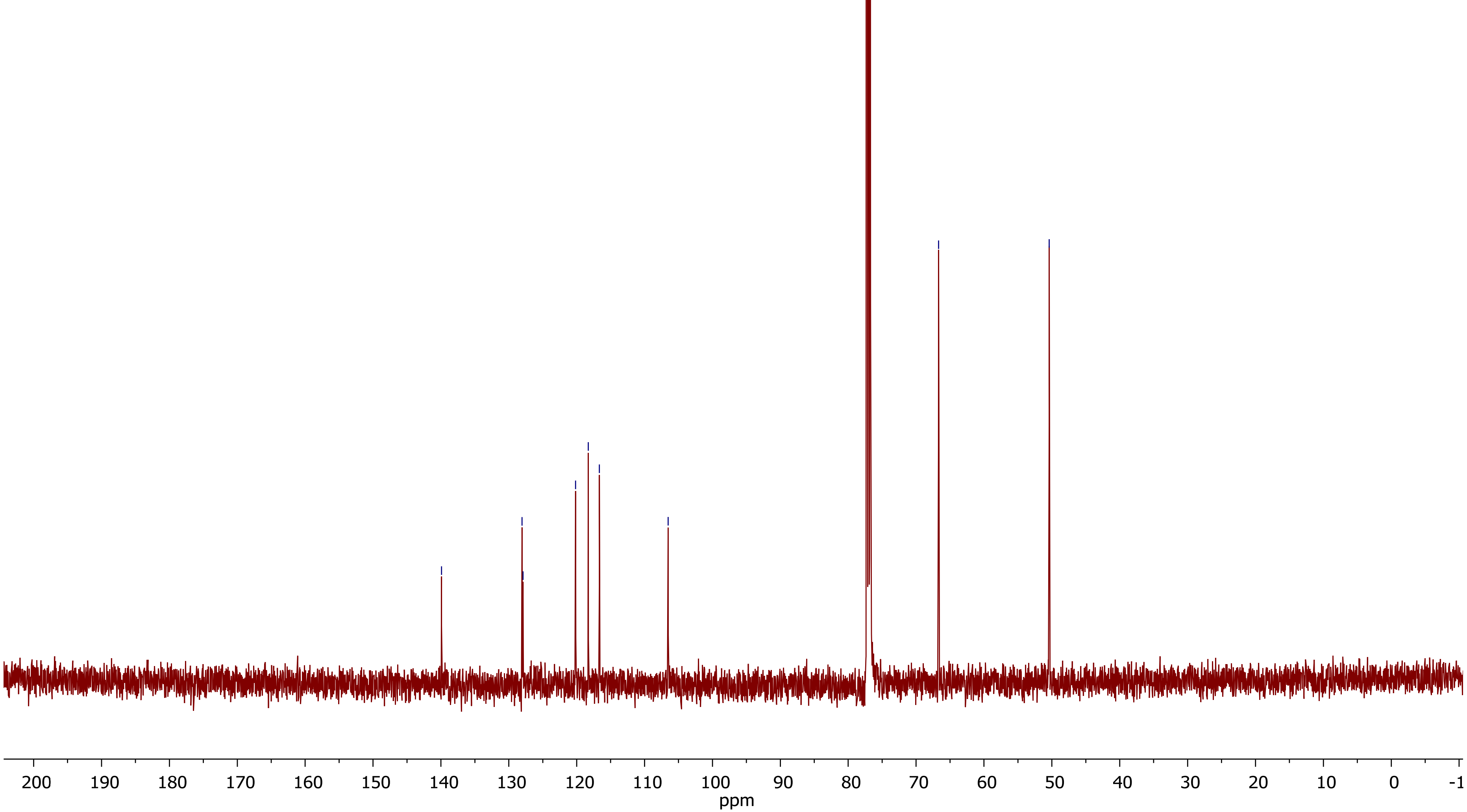

Compound 33

1H NMR (400 MHz, DMSO-d_6)

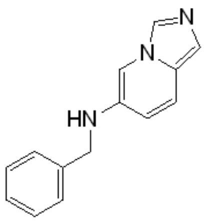

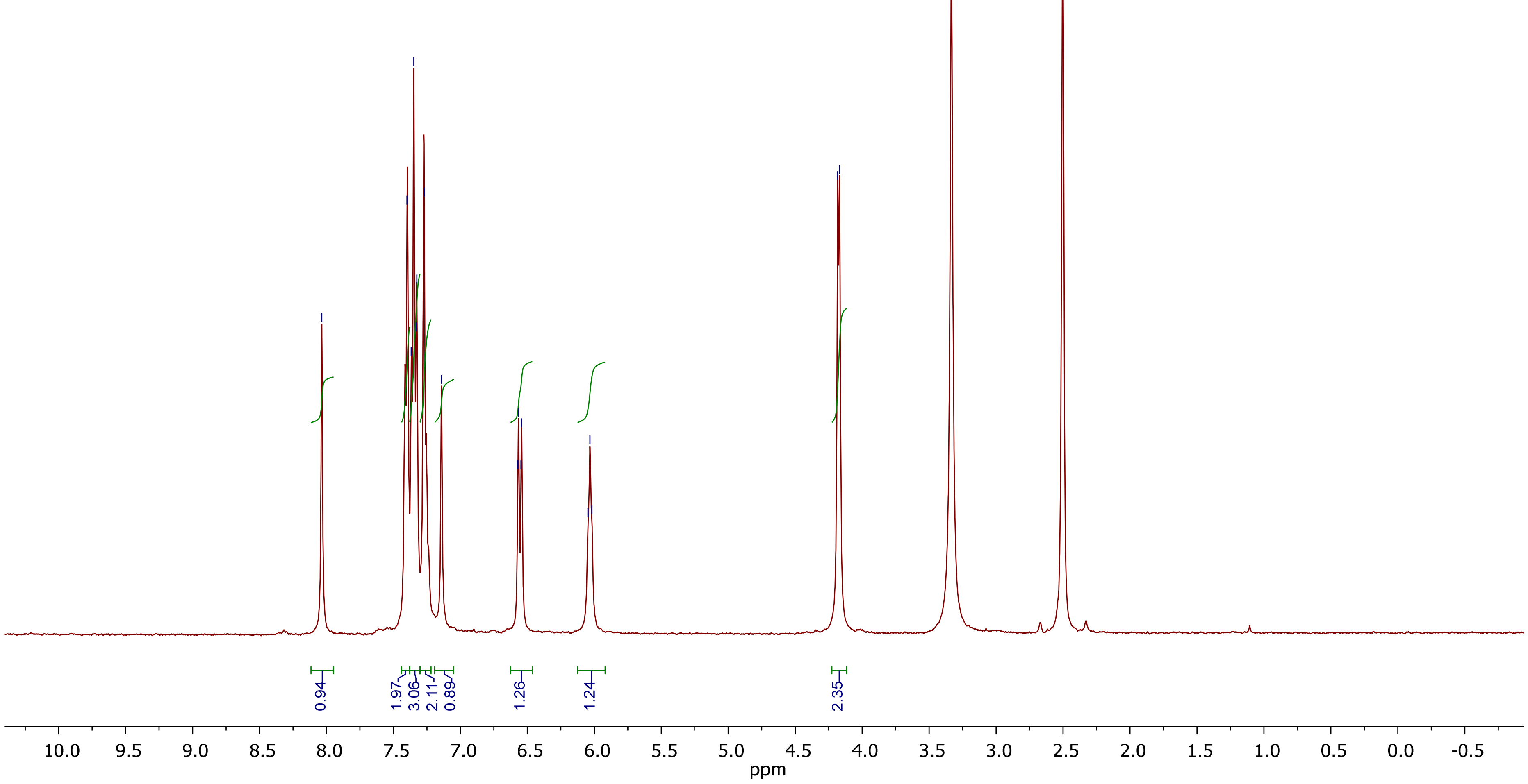

Compound 33

^{13}C NMR (101 MHz, DMSO-d_6)

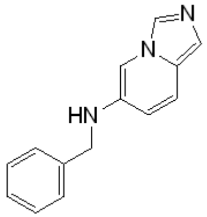

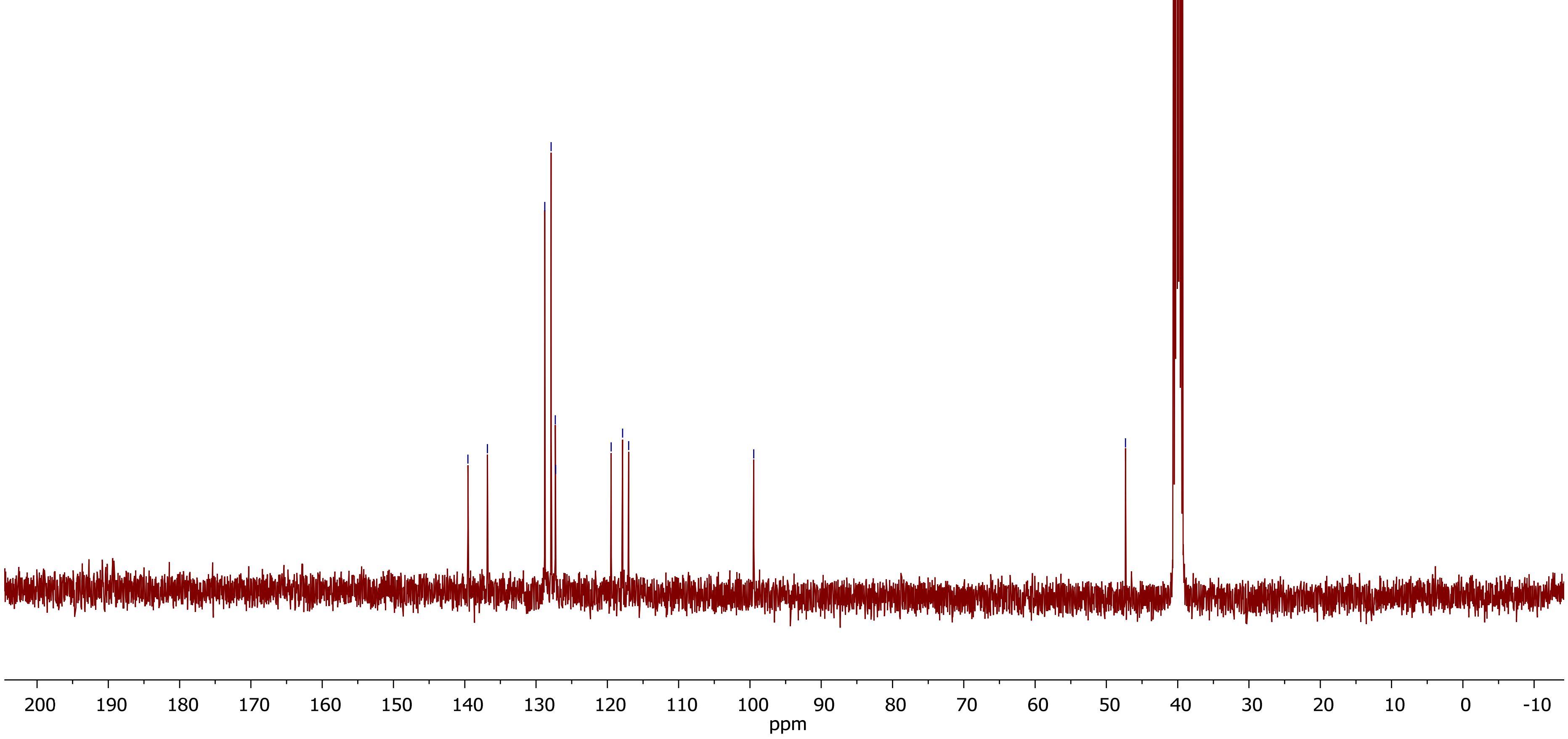

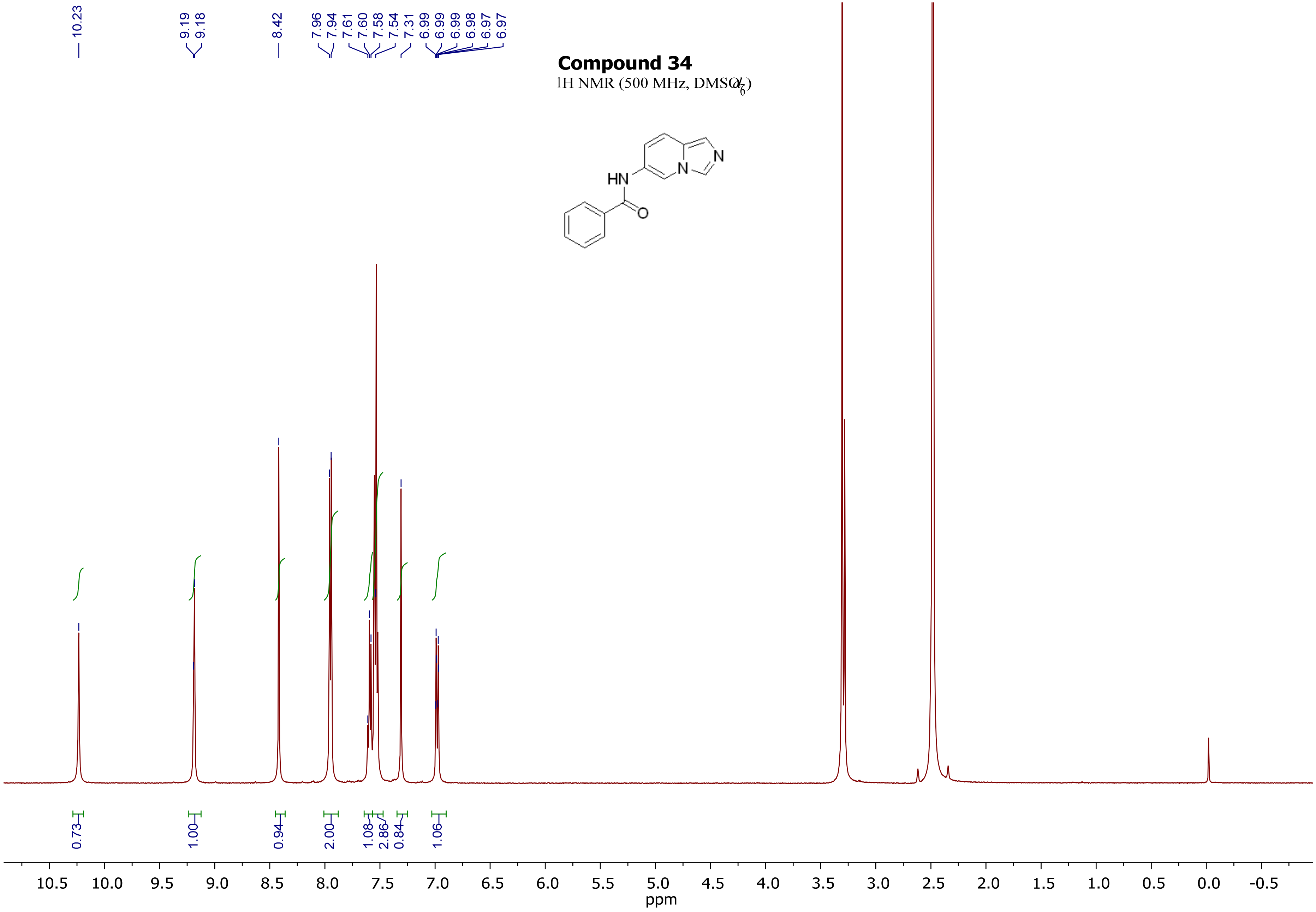

10.23
9.19
9.18
8.42
7.96
7.94
7.61
7.60
7.58
7.54
7.31
6.99
6.99
6.99
6.98
6.97
6.97
Compound 34
1H NMR (500 MHz, DMSO-d6)
HN
N
N
O
0.73
1.00
0.94
2.00
1.08
2.86
0.84
1.06
10.5 10.0 9.5 9.0 8.5 8.0 7.5 7.0 6.5 6.0 5.5 5.0 4.5 4.0 3.5 3.0 2.5 2.0 1.5 1.0 0.5 0.0 -0.5
ppm

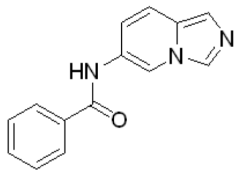

Compound 34

13C NMR (101 MHz, Chloroform-d)

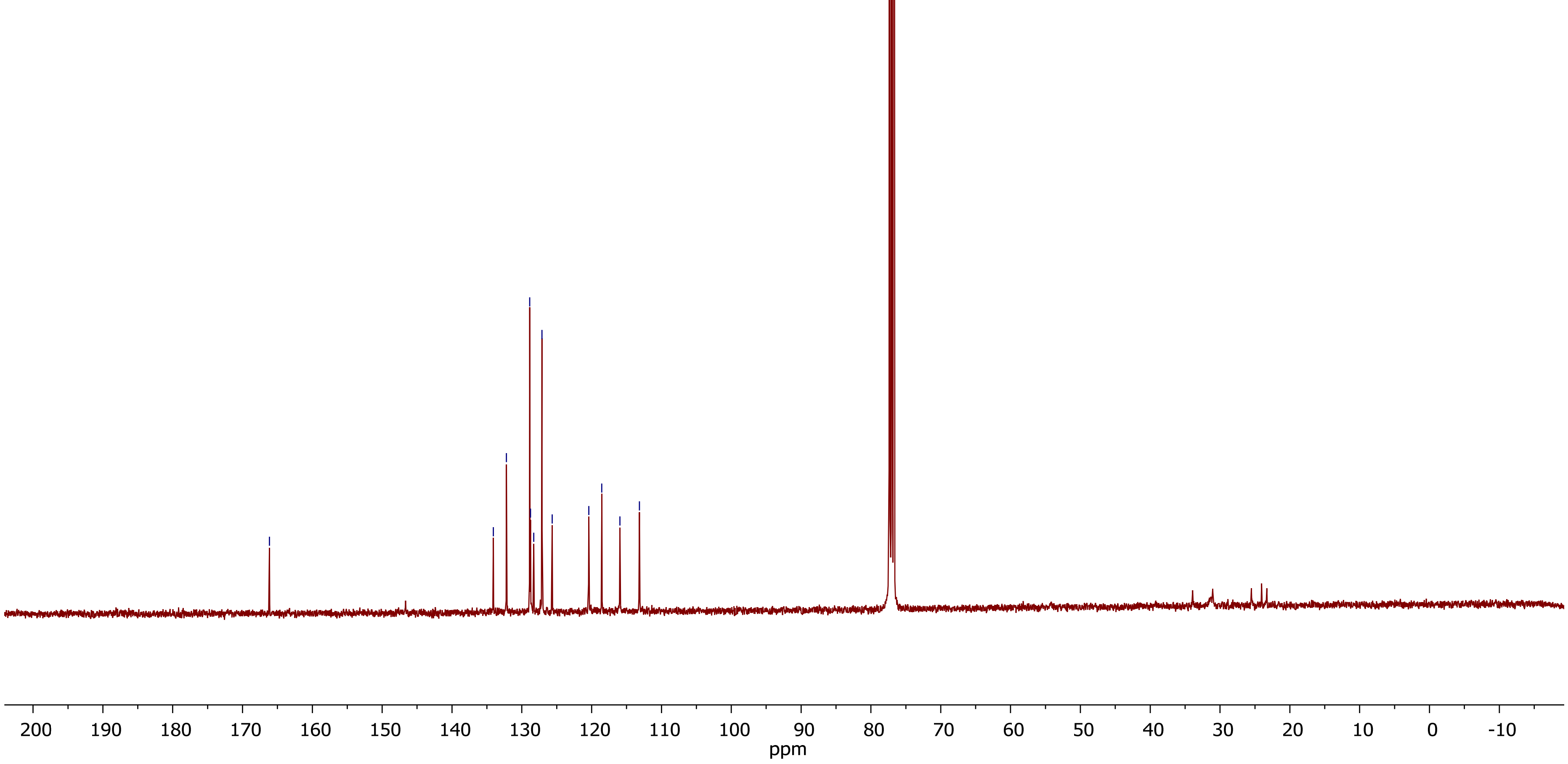

Compound 35

1H NMR (500 MHz, DMSO-d_6)

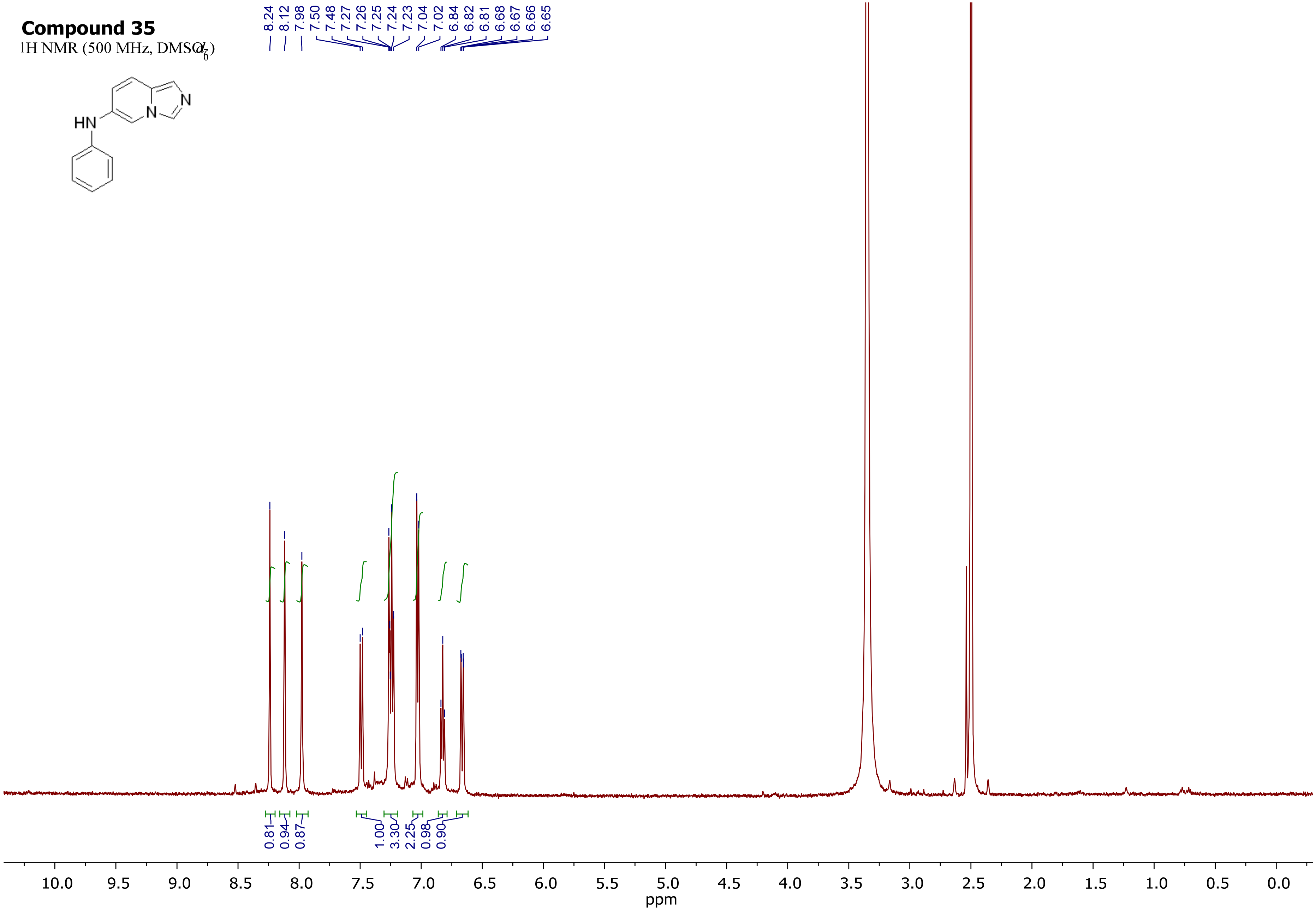

Compound 35

13C NMR (101 MHz, DMSO-d_6)

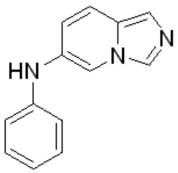

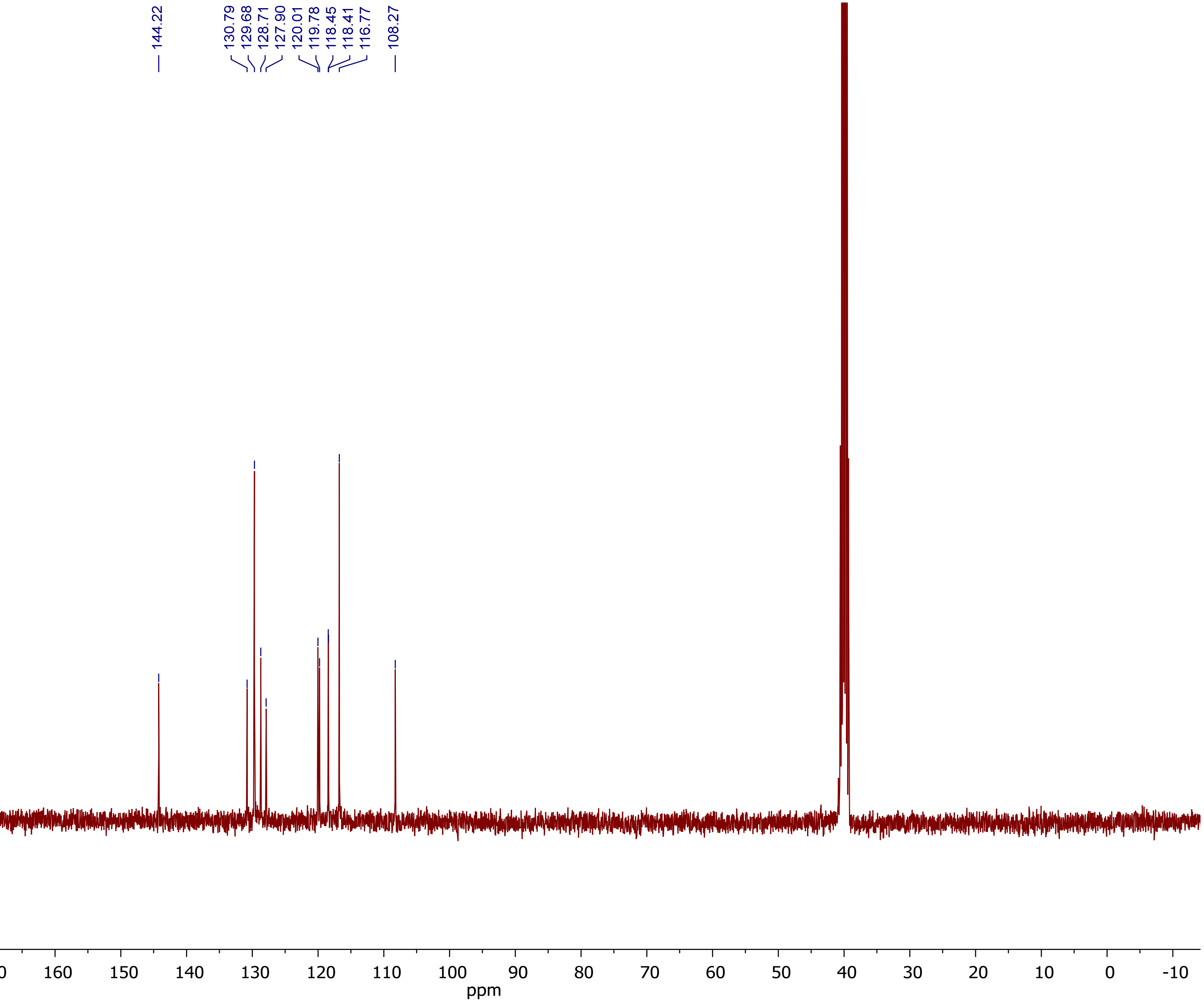

Compound 36
1H NMR (400 MHz, DMSO-d_6)

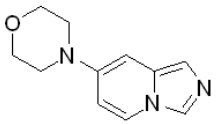

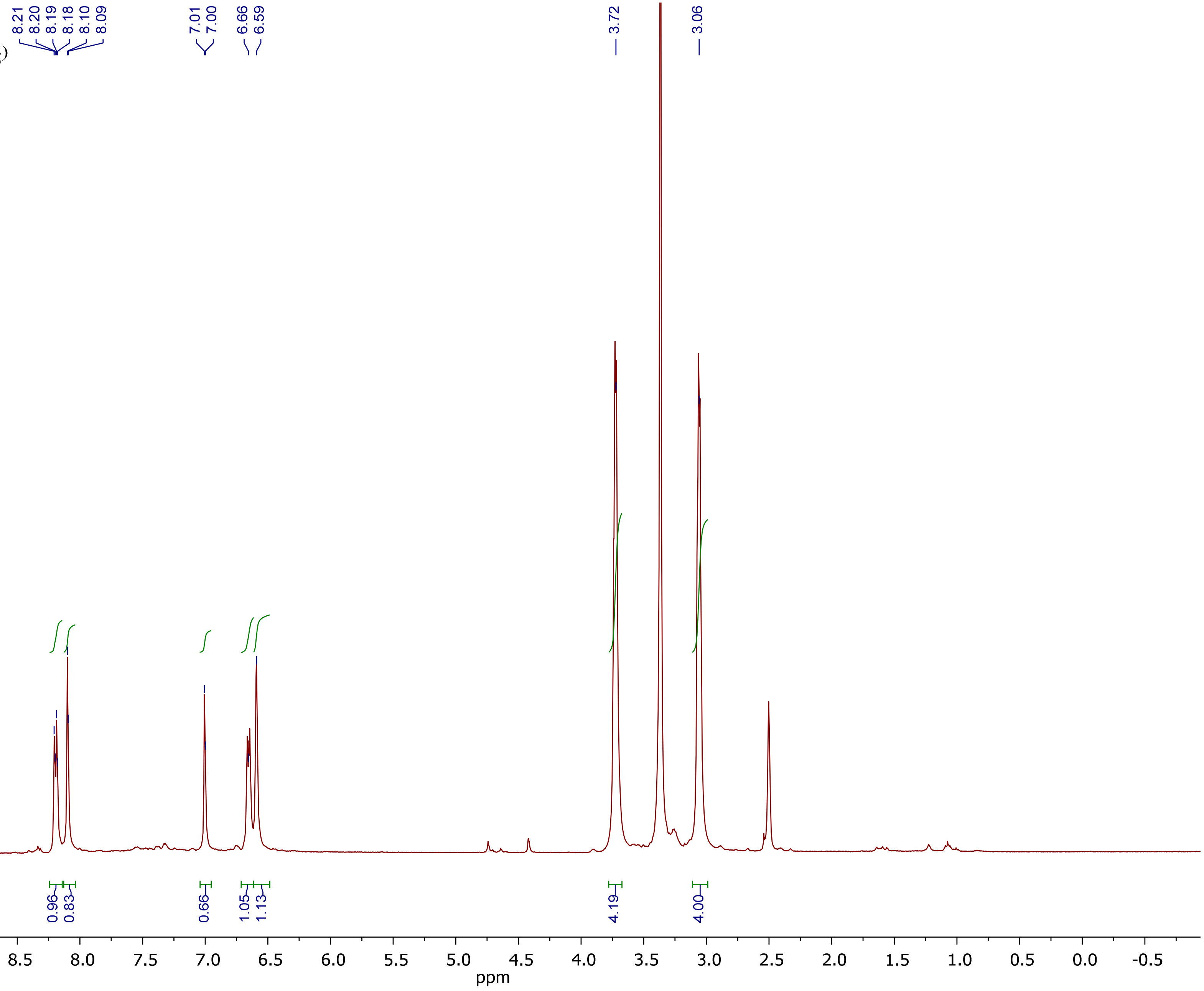

Compound 36

13C NMR (126 MHz, Chloroform-*d*)

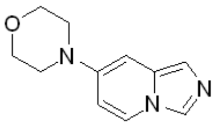

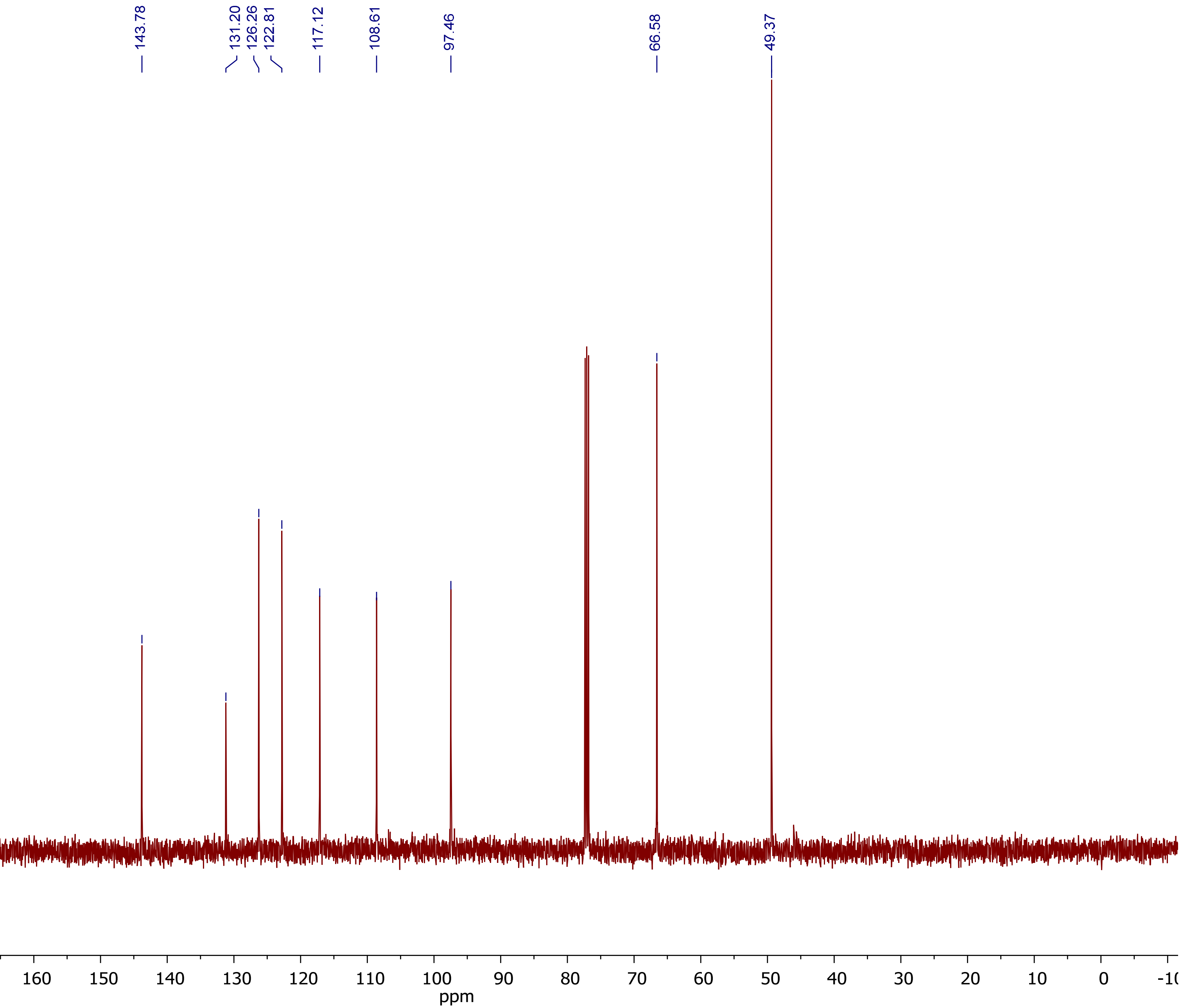

Compound 37

1H NMR (500 MHz, DMSO-d_6)

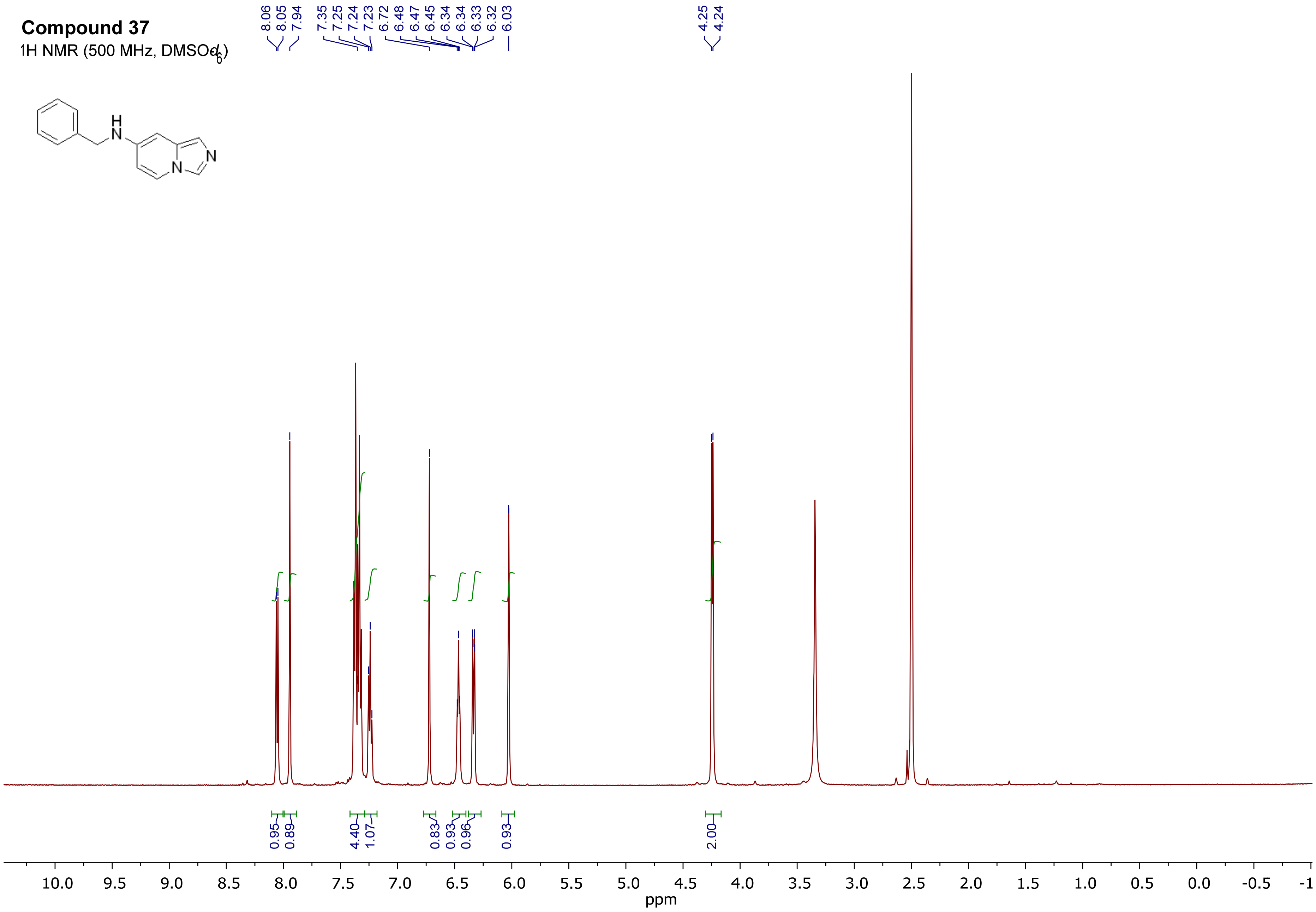

Compound 37

13C NMR (101 MHz, Chloroform-*d*)

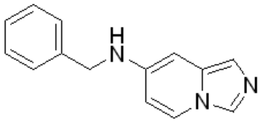

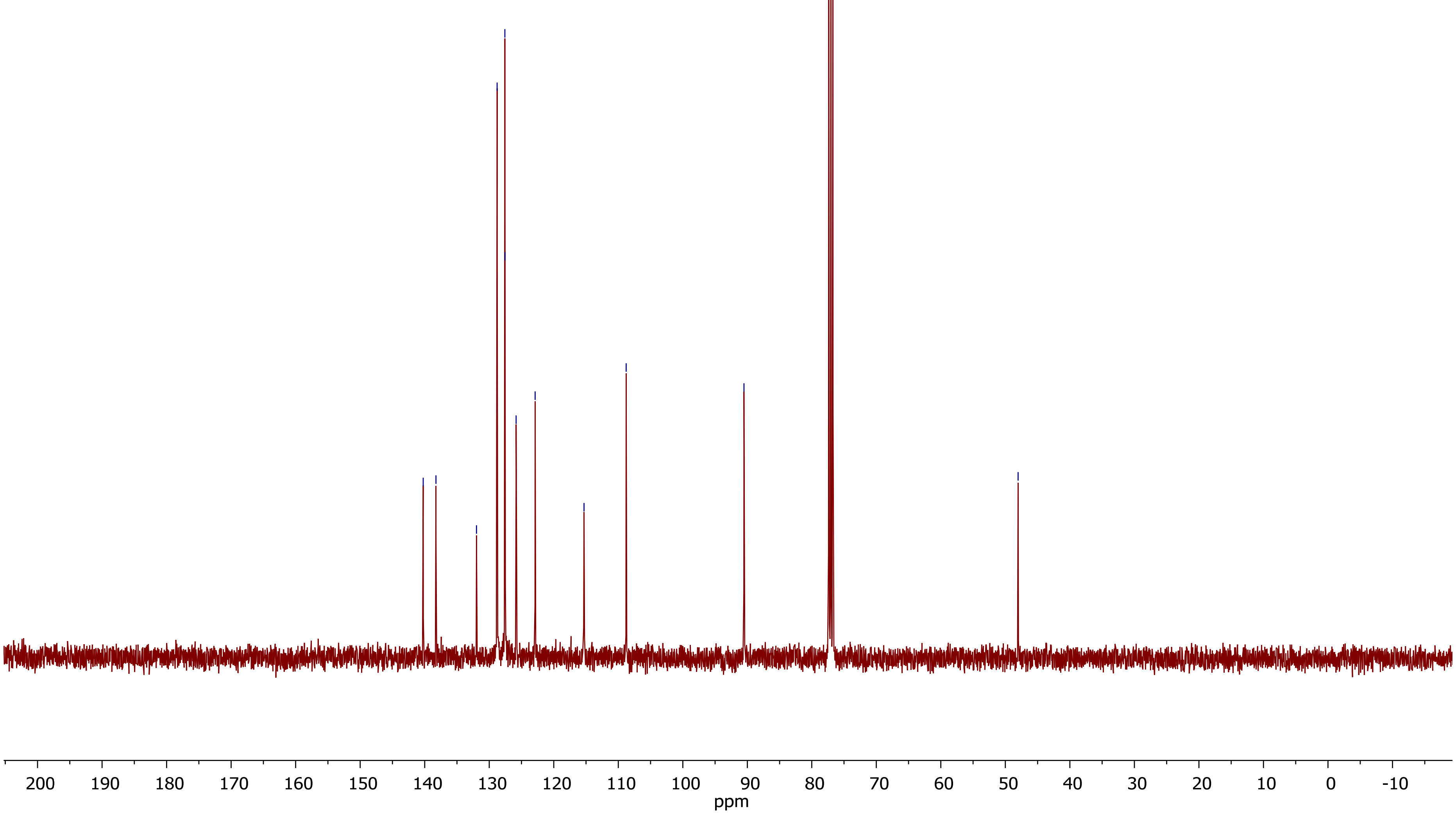

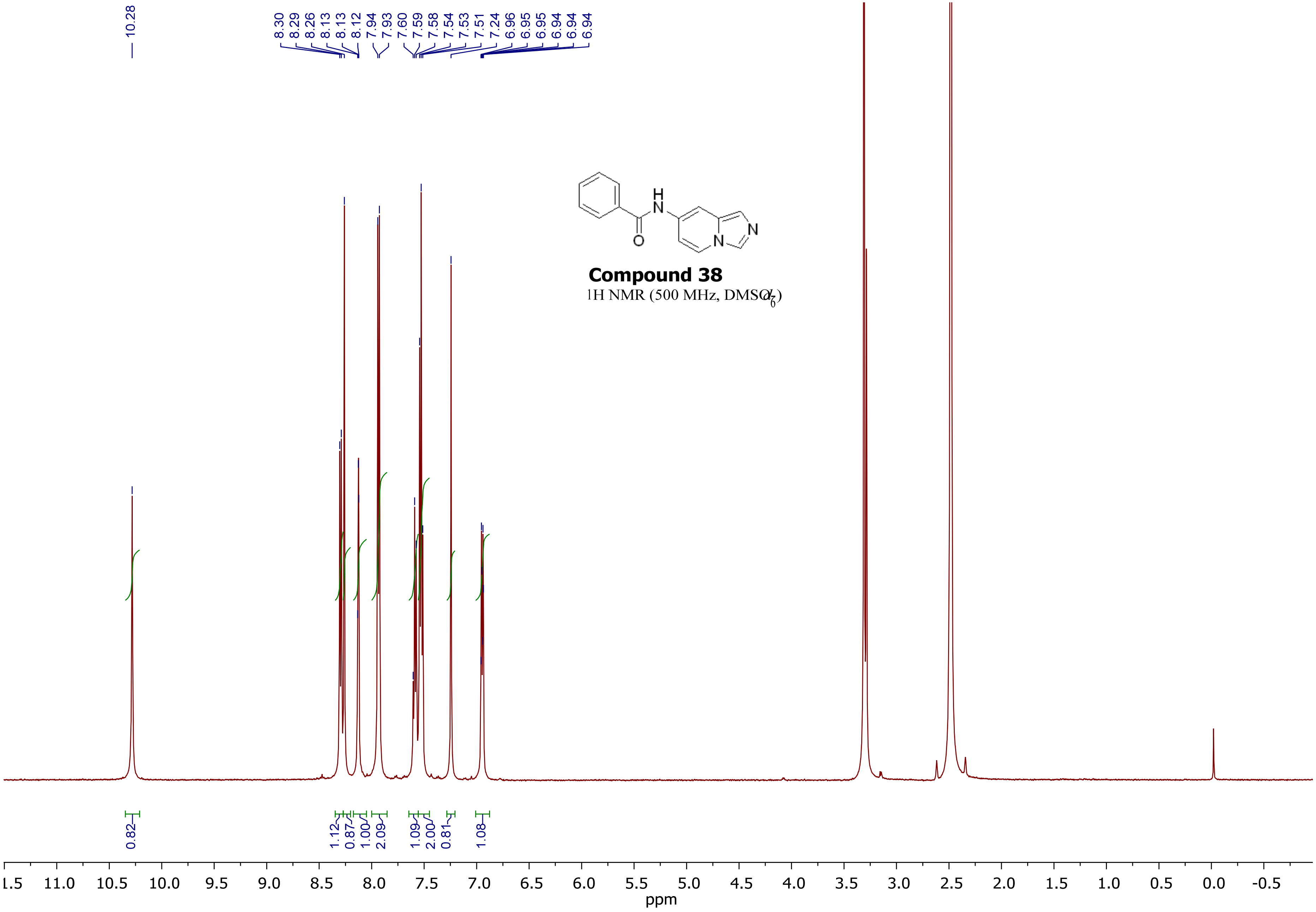

10.28
8.30 8.29 8.26 8.13 8.13 8.12 7.94 7.93 7.60 7.59 7.58 7.54 7.53 7.51 7.24 6.96 6.95 6.95 6.94 6.94 6.94
Compound 38
1H NMR (500 MHz, DMSO-d6)
0.82 1.12 0.87 1.00 2.09 1.09 2.00 0.81 1.08
ppm

Compound 38

13C NMR (151 MHz, DMSO-d_6)

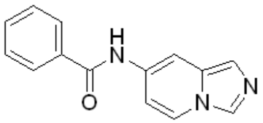

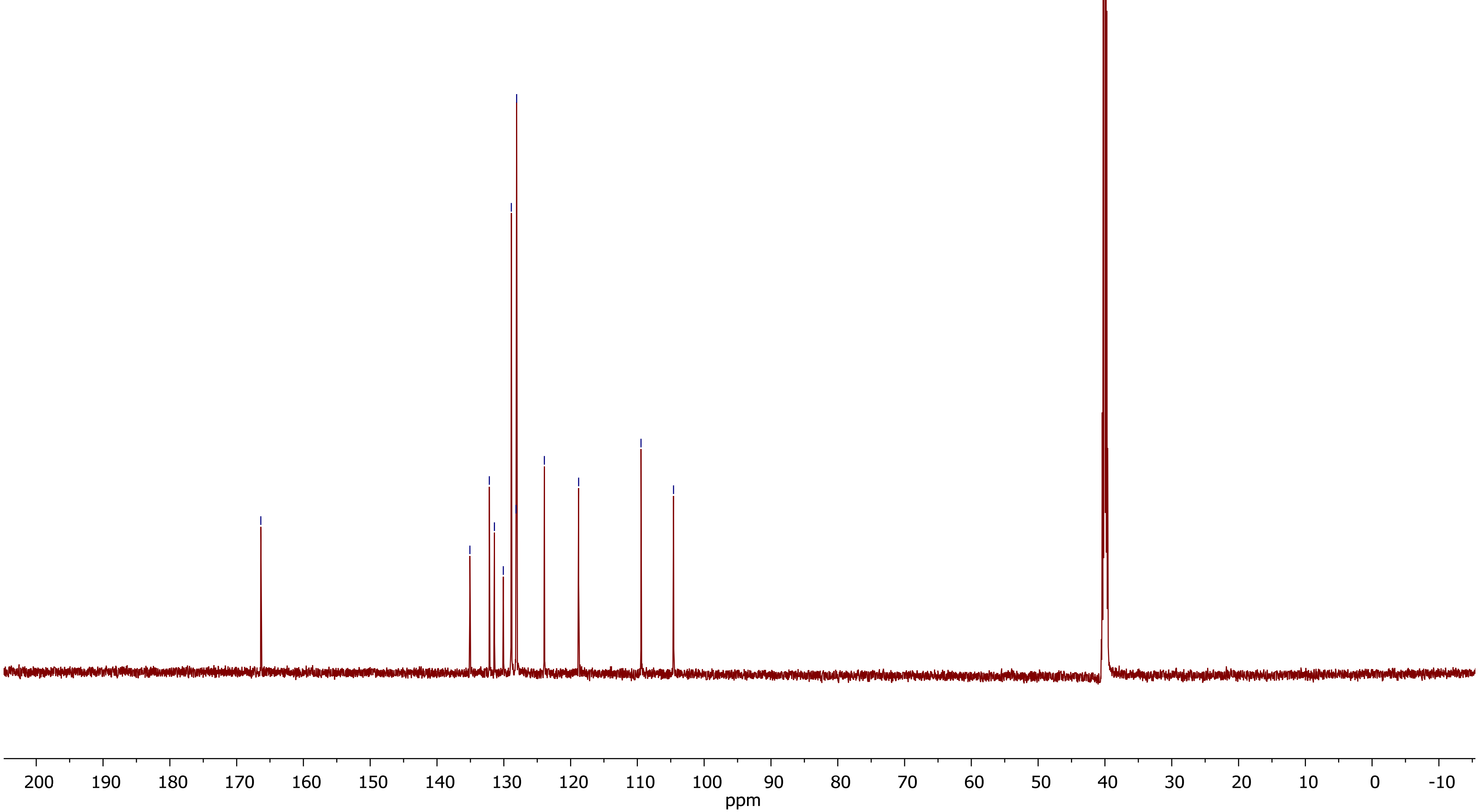

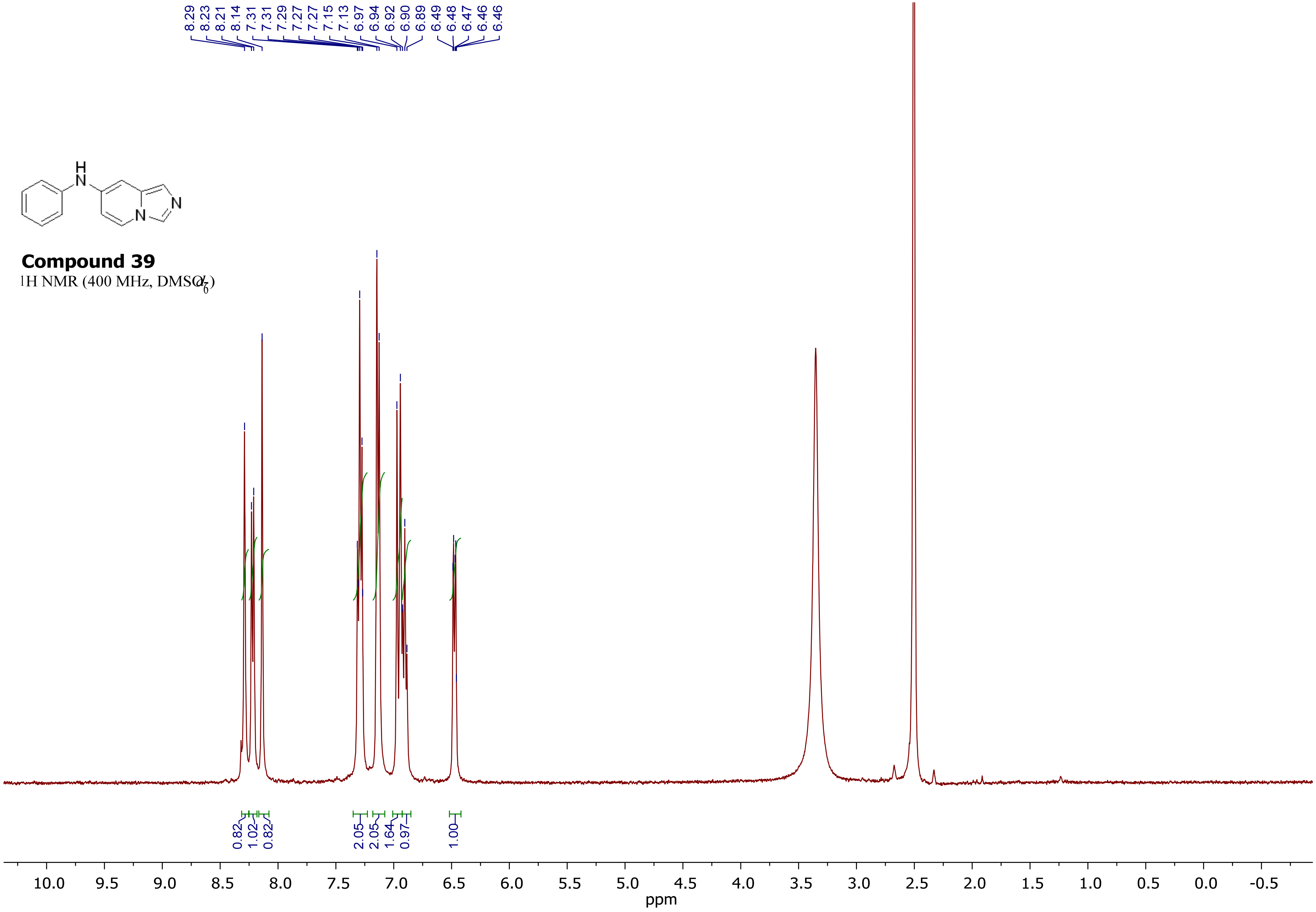

Compound 39

1H NMR (400 MHz, DMSO-d_6)

Compound 39

13C NMR (101 MHz, DMSO-d_6)

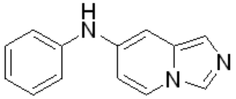

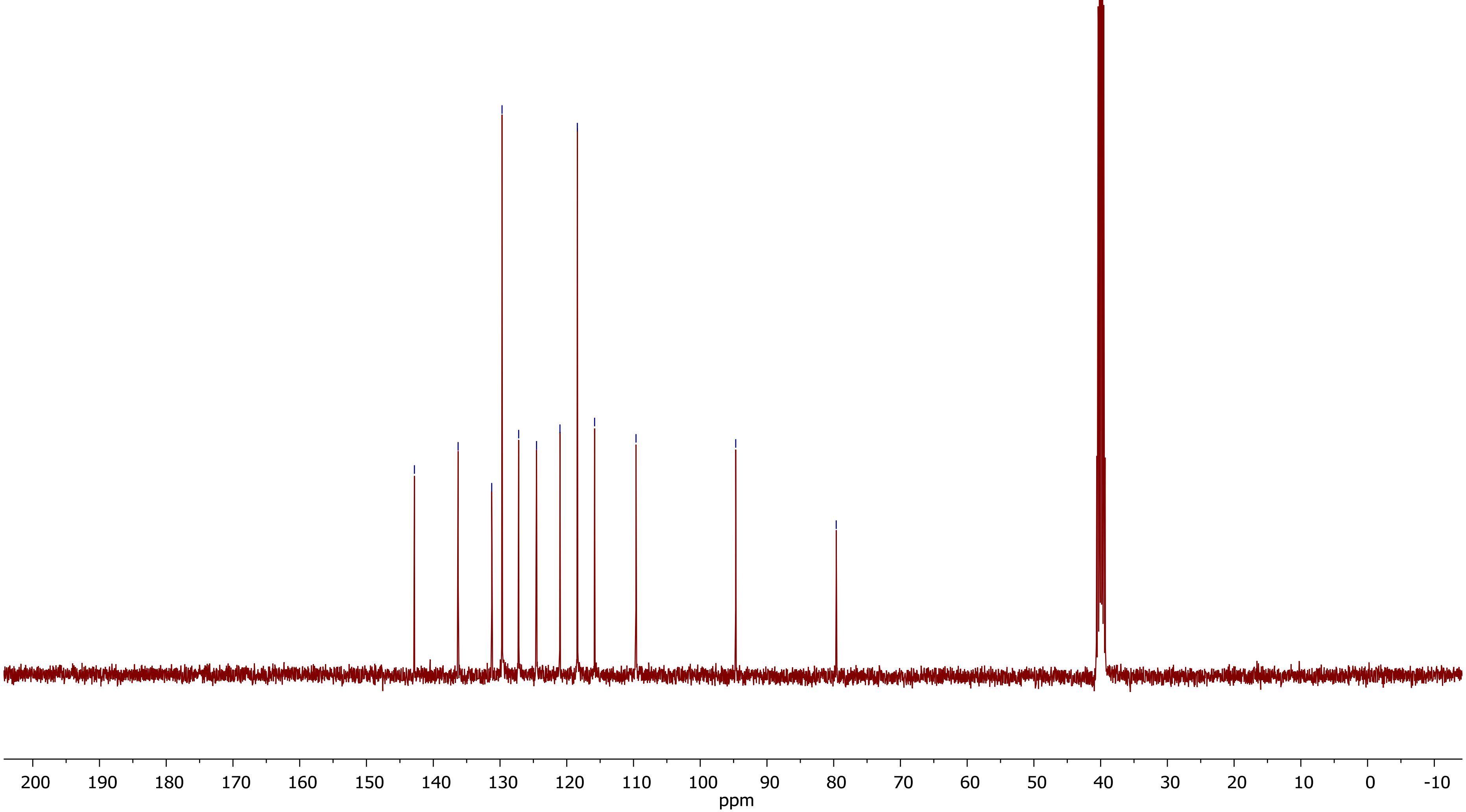

Supplement: Supplementary file 1 [file molecules-31-02339-s001.zip › molecules-4393397-supplementary.pdf]
